# An MMP-14/STAT3 feedback loop upregulates PD-L1 to drive immunosuppression and tumorigenesis in bladder cancer

**DOI:** 10.1016/j.isci.2026.118071

**Published:** 2026-10-28

**Authors:** Shuai Zhu, Lisa Köhler, Bettina Ergün, David Meierhofer, Jonas Busch, Anja Rabien

**Affiliations:** 1 Department of Urology, Hunan Provincial People’s Hospital (The 1st Affiliated Hospital of Hunan Normal University), Changsha 410000, China; 2 Department of Urology, Charité - Universitätsmedizin Berlin, Corporate Member of Freie Universität Berlin and Humboldt-Universität zu Berlin, 10117 Berlin, Germany; 3 Mass Spectrometry Facility, Max Planck Institute for Molecular Genetics, 14195 Berlin, Germany; 4 Department of Urology, Berlin Vivantes Clinic, 10967 Berlin, Germany

**Keywords:** MMP-14, PD-L1, STAT3, bladder cancer, immune escape

## Abstract

Bladder cancer is a common genitourinary malignancy in which immune checkpoint signaling contributes to treatment resistance. Matrix metalloproteinase MMP-14 degrades the extracellular matrix to facilitate cancer cell invasion, but its intracellular signaling functions remain incompletely characterized. Using TCGA data, all-human cell models, and T cell co-culture assays, we showed that MMP-14 expression correlated with clinicopathological features, negatively regulated immune processes, and was associated with regulatory T (Treg) cell enrichment. MMP-14 promoted proliferation and reduced apoptosis of bladder cancer cells and, in co-culture, suppressed T-cell-mediated killing. Mechanistically, MMP-14 was regulated by STAT3 and directly interacted with STAT3, promoting STAT3 phosphorylation at Y705 and upregulating PD-L1 expression. These results define an MMP-14/STAT3/PD-L1 axis that drives T cell dysfunction and Treg accumulation in bladder cancer, suggesting a target for overcoming immunotherapy resistance.

## Highlights

• MMP-14 promotes bladder cancer cell growth and immune evasion
• MMP-14 physically binds STAT3 to induce its Y705 phosphorylation
• A positive feedback loop between MMP-14 and STAT3 drives PD-L1 expression
• The MMP-14/STAT3 axis promotes T cell dysfunction and Treg enrichment

## Graphical abstract

**Figure:**
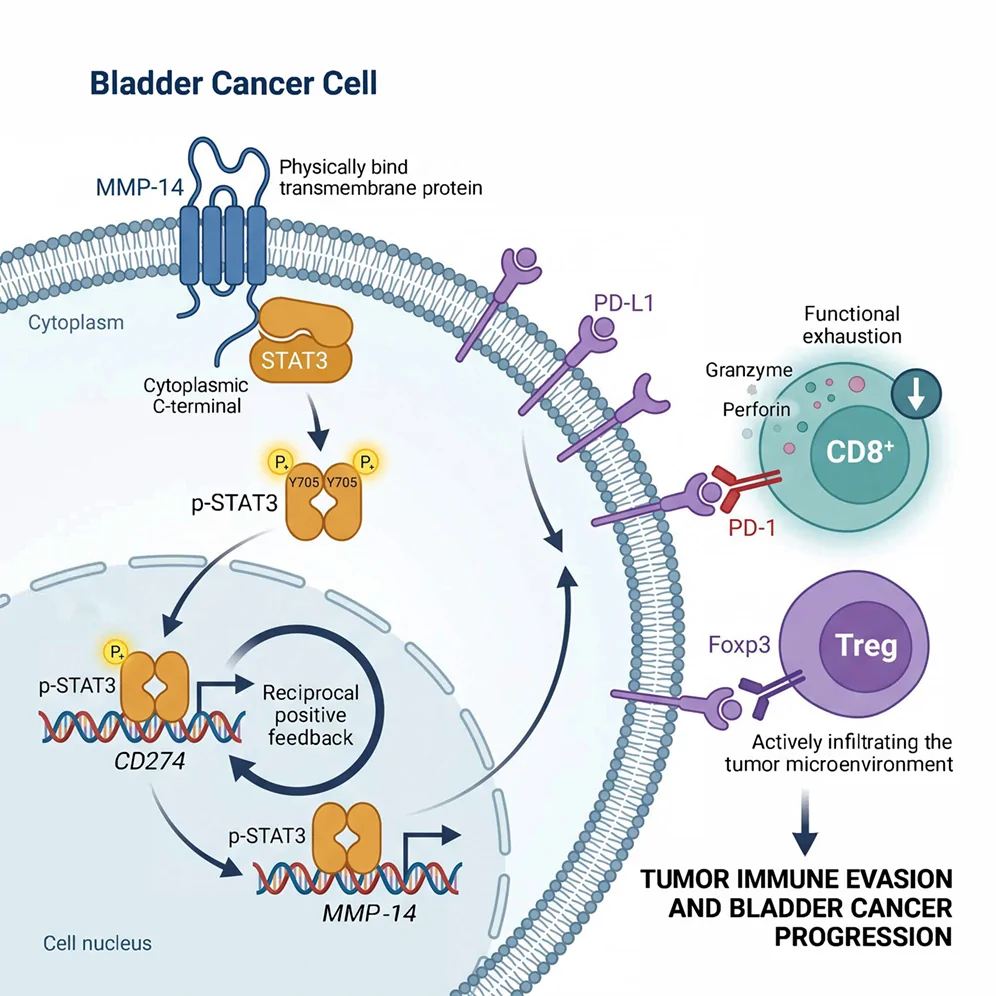

## Teaser

Biological sciences; Systems biology; Cancer systems biology; Cancer

## Introduction

Bladder cancer, usually urothelial carcinoma, represents one of the most common genitourinary malignancies. In 2025, 84870 new cases and 17420 bladder cancer-related deaths were estimated in the United States.^1^ Approximately 75% of bladder cancers are initially diagnosed as non-muscle-invasive bladder cancer (NMIBC), while the remaining 25% are muscle-invasive bladder cancer (MIBC).^2^ However, NMIBC is characterized by high recurrence and may progress to MIBC, which results in intensive monitoring and costly management.^3^ Up to 40% of patients with MIBC experience recurrence following cystectomy, and the majority of them ultimately succumb to bladder cancer.^4^ Therefore, understanding the underlying molecular mechanisms of bladder cancer can help to develop new bladder cancer treatment strategies to improve outcomes for patients with bladder cancer.

Matrix metalloprotease 14 (MMP-14, MT1-MMP) is a membrane-anchored metalloprotease classically recognized for its collagenase activity involved in extracellular matrix (ECM) degradation and cancer cell invasion.^5^ MMP-14 not only degrades the ECM itself but also promotes cancer cell secretion and activation of pro-MMP-2 and pro-MMP-9.^5^^,^^6^ Moreover, MMP-14 cleaves other functional surface proteins and integrity factors.^5^ Thus, the proteolytic activity of MMP-14 contributes to the maintenance of the cancer microenvironment and plays a pivotal role in cancer growth, migration, invasion, angiogenesis, and metastasis.^5^ Interestingly, MMP-14 also regulates cell growth and motility through nonproteolytic pathways mediated by its highly conserved 20-amino acid cytoplasmic tail.^6^ Some studies have focused on the function of MMP-14 in immunity,^5^^,^^7^^,^^8^ such as negatively regulating the inflammatory response by modulating alarmin cascades,^7^ though its specific intracellular signaling mechanisms remain largely obscured. A recent study in bladder cancer associates high expression of MMP-14 with poorer response to immune checkpoint inhibitors.^9^ However, the exact molecular network by which MMP-14 drives this profound immune resistance requires deeper elucidation.

The cells of our body are continuously under the surveillance of the immune system, which can promptly clear abnormal cells but cancer cells can express the immune checkpoint molecule programmed cell death ligand 1 (PD-L1) and block their recognition through its receptor PD-1, resulting in immune escape.^10^^,^^11^^,^^12^ PD-L1 belongs to the immunoglobulin superfamily and is, besides in cancer cells, expressed in several cell types, e.g., stromal cells of the tumor environment and antigen-presenting cells contributing to immune escape of the tumor.^13^ When PD-1 and PD-L1 interact, downstream signaling leads to rapid functional suppression of T cell proliferation, cytokine production and cytotoxic activity, while simultaneously enhancing immunosuppression by regulatory T (Treg) cells.^13^ In bladder cancer, the transcription factors c-Jun and STAT3 are reported to enhance PD-L1 expression.^13^ STAT3 is a transcription factor involved in multiple physiological processes, such as cell proliferation, apoptosis, angiogenesis, and immune response.^14^ STAT3 also plays an important role in accelerating cancer cell proliferation and resisting apoptosis,^14^ as known for bladder cancer, in which STAT3 is increased with invasiveness and able to induce drug resistance.^15^

Drug resistance is a big challenge for therapeutic interventions using immune checkpoint inhibitors. Despite their clinical relevance in numerous bladder cancer trials, many patients still exhibit primary or acquired resistance.^13^^,^^16^^,^^17^ Therefore, comprehensively dissecting the molecular regulatory mechanisms underlying immune checkpoints is crucial. The current findings propose a potential, self-sustaining positive feedback loop: MMP-14 acts as an intracellular non-proteolytic scaffold to physically interact with and activate STAT3, which in turn transcriptionally upregulates PD-L1 to orchestrate acute effector T cell functional suppression and Treg expansion. Furthermore, to rigorously validate this human-specific signaling axis and deliberately bypass the severe phylogenetic evolutionary defects inherent in murine PD-1 receptors, which lack the crucial pre-ITSM PEQ motif required for profound immunosuppression,^18^ our study strategically integrates all-human *in vitro* co-culture models with large-scale real-world clinical cohort analyses. This approach provides preliminary mechanistic insights into how the MMP-14/STAT3/PD-L1 axis governs immune evasion, offering novel theoretical foundations for combination immunotherapies in advanced bladder cancer.

## Results

### Clinicopathological characteristics and transcriptomic enrichment analysis of MMP-14 in bladder cancer

To establish the clinical significance of MMP-14 in bladder cancer, we first evaluated The Cancer Genome Atlas (TCGA) dataset. As shown in Table S1, the Cancer Genome Atlas (TCGA) data on MMP-14 expression were significantly associated with patient race, T stage, pathological stage, subtype, and radiation therapy but not with the other parameters. African, Black American and White people had greater MMP-14 expression than did Asian people. For subtype, nonpapillary cancers had markedly higher levels than papillary cancers, and patients who had received preoperative radiation therapy had significantly lower MMP-14 levels than those who did not. However, given that certain demographic and clinical subgroups within the TCGA dataset possess rather small case numbers, these association analyses should be explicitly labeled as exploratory and preliminary. The generalizability of these conclusions awaits rigorous validation in independent cohorts with larger, more balanced sample sizes. With respect to tumor stage, we detected greater MMP-14 expression in patients with T3 stage disease than in patients with T2 stage disease. Based on these significant clinical correlations, we further conducted transcriptomic analyses to uncover the broader biological networks influenced by MMP-14. We screened differentially expressed genes (DEGs) from TCGA data according to the different expression levels of MMP-14, identifying 1798 DEGs (Figure 1A; Table S2). Additionally, we found 3955 correlated genes (CGs) in TCGA data (Table S3). The 767 common DEGs and CGs displayed in a Venn diagram (Figure 1B; Table S4) were used for further analyses. Crucially, Gene Ontology (GO) enrichment analysis of biological processes revealed that these genes were also enriched in the term “negative regulation of the immune system process” (Figure 1C). Gene set enrichment analysis (GSEA) showed that these genes were significantly enriched in key immune-regulating pathways, notably PD-1 signaling and T cell receptor signaling (Figure 1D).

**Figure 1:**
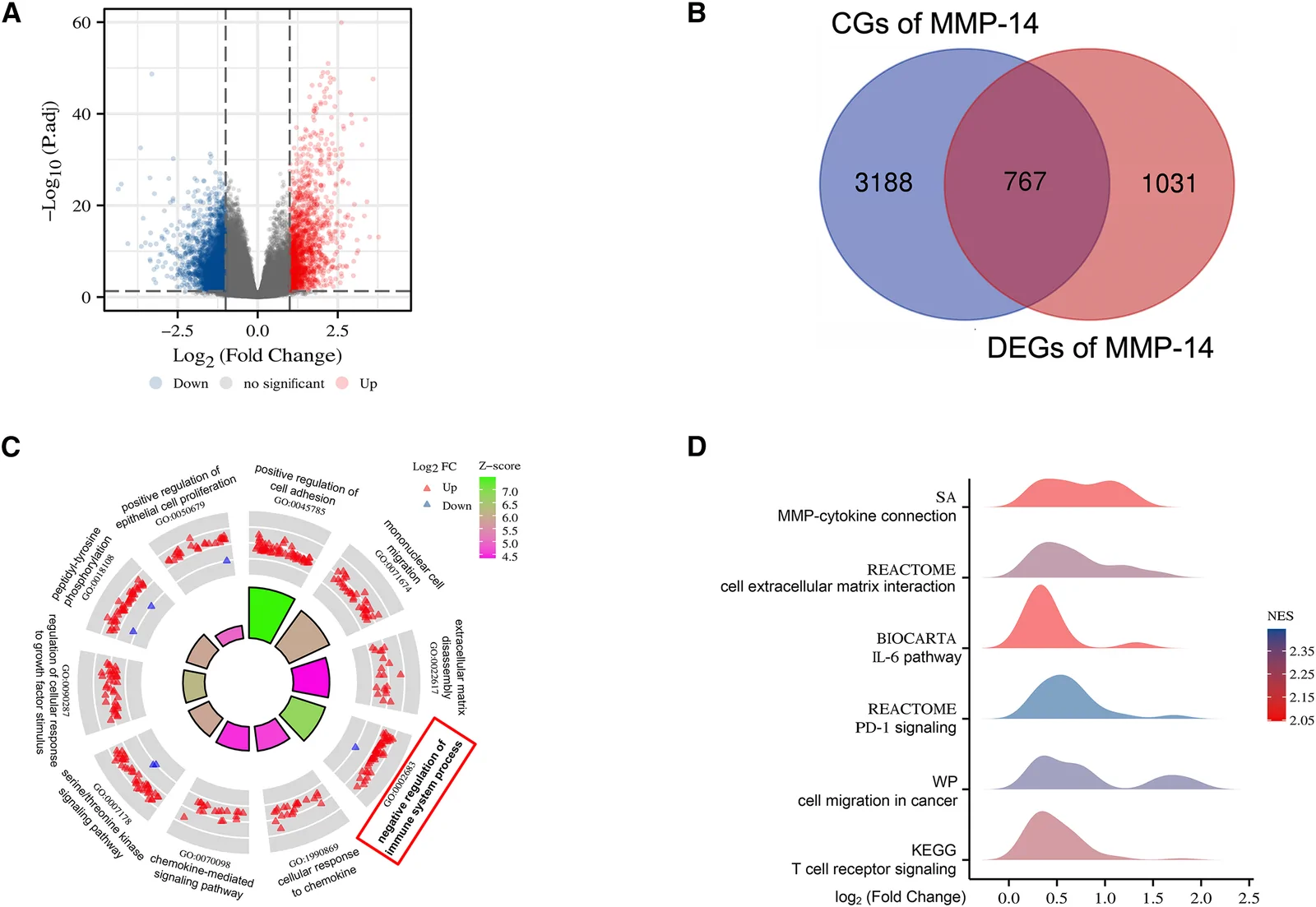
Enrichment analysis of differentially expressed genes (DEGs) and correlated genes (CGs) for MMP-14 in bladder cancer from TCGA data (A) A volcano plot was used to screen the DEGs between the high MMP-14 expression group and the low MMP-14 expression group in bladder cancer. The screening conditions were as follows: abs |log2(fold change)| > 1 and adjusted *p* value <0.05. (B) Venn diagram shows the genes common to both DEGs and CGs; an abs |correlation value| > 0.3 and a *p* value <0.05 were considered to indicate correlations. (C) Gene Ontology (GO) enrichment analysis of the main biological processes connected to MMP-14 based on the screened public genes. (D) Gene set enrichment analysis (GSEA) of the main biological functions of MMP-14 in bladder cancer. NES: normalized enrichment score, which refers to the mean of all enrichment scores from all random permutations for the gene set tested.

### Expression correlation of MMP-14 with PD-L1 and the *in vitro* anti-tumor effects of its pharmacological inhibition

Given the robust bioinformatic evidence linking MMP-14 to immunosuppression and PD-1 signaling, we subsequently assessed the direct relationship between MMP-14 and the immune checkpoint ligand PD-L1 (CD274) at the cellular level. Analyses of TCGA data proved the CD274 gene (PD-L1) to be upregulated in DEGs (Table S2; log_2_(fold change) 1.763, *p* value <0.0001) and positively correlated in CGs (Table S3; Pearson R = 0.346, *p* value <0.0001). Immunofluorescence analysis further confirmed that both MMP-14 and PD-L1 were highly expressed in HT-1376 cells, whereas their expression levels were proportionally lower in UM-UC-3, RT-4, and RT-112 cells (Figure 2A), establishing their co-expression pattern *in vitro*. To evaluate the functional consequences of targeting MMP-14, we introduced the MMP-14-specific inhibitor NSC405020. We treated the bladder cancer cell lines with different concentrations of the MMP-14-specific inhibitor NSC405020 for 48 h and determined their respective IC50 (Figure S1A). Notably, NSC405020 is a highly selective allosteric inhibitor that binds the extracellular hemopexin (PEX) domain of MMP-14; it specifically blocks PEX-dependent homodimerization and downstream protein-protein interactions without affecting the intrinsic zinc-dependent catalytic (enzymatic) activity of the enzyme. Treatment with NSC405020 inhibited the overall protein expression of MMP-14 (Figure 2B). In all cells but RT-4 cells (not significant), NSC405020 markedly downregulated PD-L1 expression as well (Figure 2B). By disrupting homodimerization-dependent functional complex formation, treatment with NSC405020 decreased the detectable active form of MMP-14 on the cell surface (Figure 2C), as well as cell viability compared to control (Figure 2D). The apoptosis index in the NSC405020 treatment group considerably increased at 48 h compared with that in the corresponding controls (Figure 2E), but cytotoxicity also increased up to this time point (Figure S1B).

**Figure 2:**
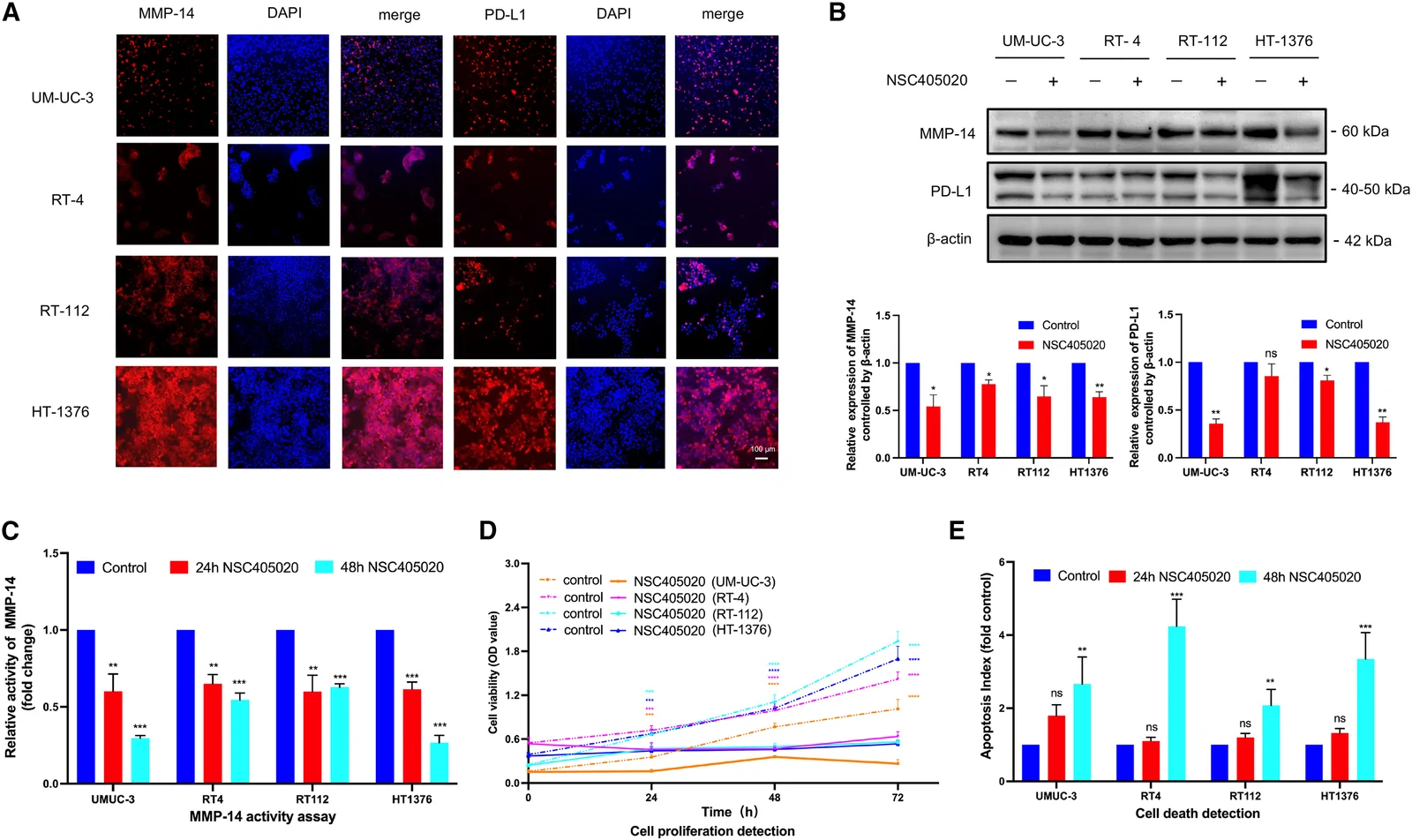
Effect of NSC405020 and its relationship to PD-L1 in bladder cancer cells (A) Immunofluorescence was used to detect the cell surface expression of MMP-14 and PD-L1 in the bladder cancer cell lines UM-UC-3, RT-4, RT-112 and HT-1376. Nuclei were stained with bisbenzimide. Scale bars, 100 μm. (B) Western blotting shows the changes in the expression of MMP-14 and PD-L1 after 24 h of treatment with NSC405020. Significant results were determined by an independent *t* test; (*n* = 3). (C) MMP-14 activity in bladder cancer cells treated with NSC405020 for 24 h or 48 h. One-way ANOVA was used to identify significant results, and Dunnett’s procedure was used for multiple comparisons (*n* = 3). (D) WST-8 assays were used to detect the effect of NSC405020 on cell proliferation after 24 h, 48 h, and 72 h of NSC405020 treatment in bladder cancer cells. Two-way ANOVA was employed to detect significant results, and Bonferroni’s multiple comparison approach was used; (*n* = 3). (E) A cell death detection assay was used to assess the apoptosis of bladder cancer cells after 24 h and 48 h of NSC405020 treatment. One-way ANOVA was used to identify significant results, and Dunnett’s procedure was used for multiple comparisons; (*n* = 3). Data are represented as mean ± SD. ns, not significant; ∗*p* < 0.05, ∗∗*p* < 0.01, and ∗∗∗*p* < 0.001.

### Genetic targeting of MMP-14 confirms its crucial role in regulating PD-L1 and driving tumorigenesis

To rule out potential off-target effects of the small molecule inhibitor and to rigorously confirm the causal link between MMP-14, PD-L1, and cell survival from a genetic perspective, we constructed stable genetically modified bladder cancer cell lines. Using HT-1376 cells, which possess high endogenous expression, we established stable MMP-14 knockdown (KD) clones. Consistent with the pharmacological inhibition data, MMP-14 knockdown directly led to a significant decrease in PD-L1 expression (Figure 3A). Additionally, while the absolute differences in 2D cell proliferation were visually modest, the cell viability of KD clones was statistically significantly lower at 96 h compared to controls (Figure 3B). In contrast, the apoptotic indices were robustly greater at 48 h and 96 h (Figure 3C), indicating a predominant effect on cell survival. Conversely, upon establishing MMP-14 overexpression (OE) models in low-expressing RT-112 cells, all OE clones not only exhibited increased MMP-14 expression but also synchronously induced the upregulation of PD-L1 (Figure 3D). Functionally, all overexpression clones displayed a modest but statistically significant increase in 2D cell viability at 96 h (Figure 3E), alongside apoptosis indices that were substantially lower than those of the control groups (Figure 3F). These orthogonal genetic data strongly support the hypothesis that MMP-14 is involved in the intrinsic regulation of PD-L1 expression levels while driving the malignant proliferation of cancer cells.

**Figure 3:**
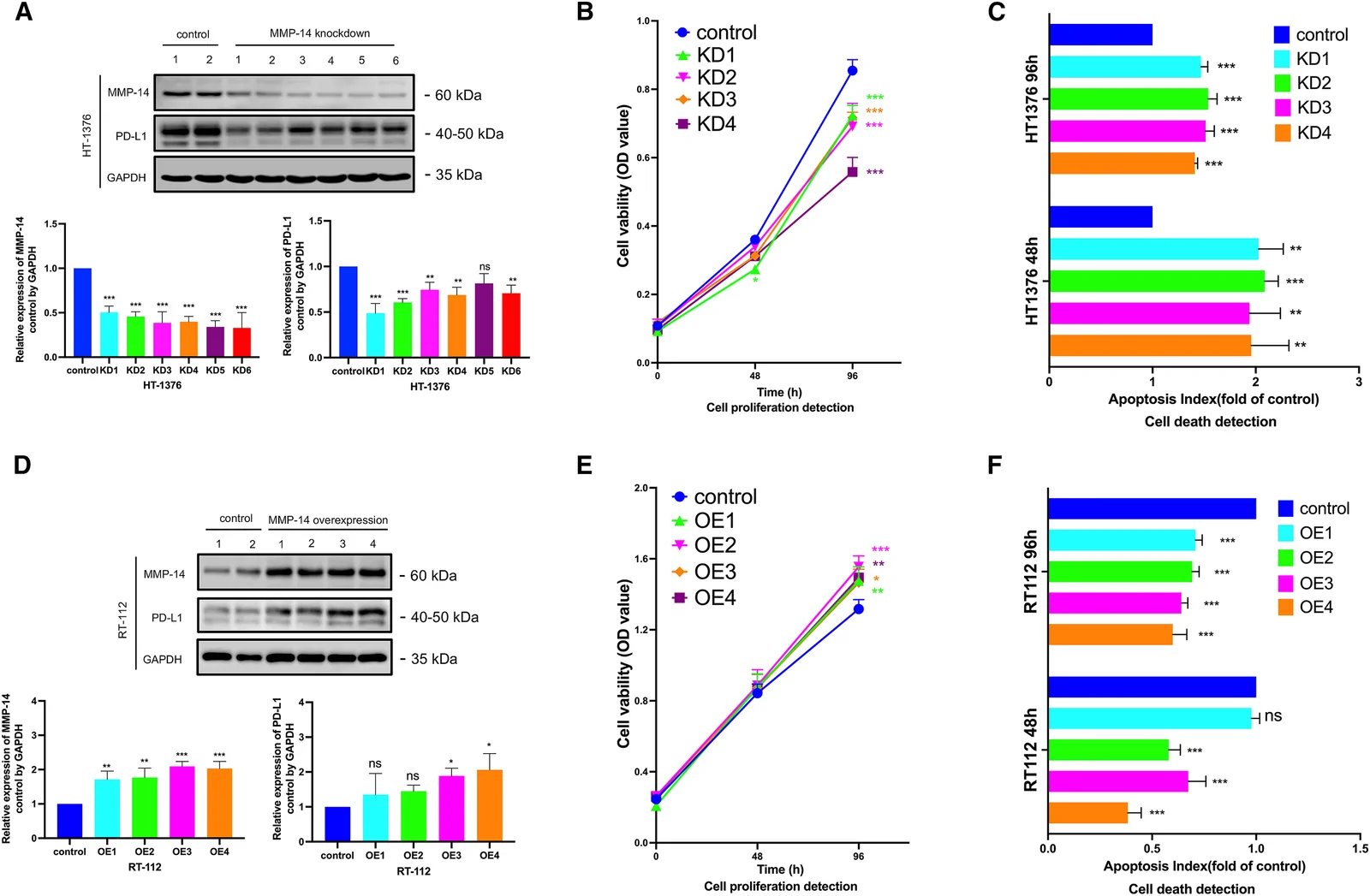
The function of MMP-14 and its relationship to PD-L1 in bladder cancer cells (A) Western blotting shows the expression levels of MMP-14 and PD-L1 in control HT-1376 cells and MMP-14 knockdown HT-1376 cells. One-way ANOVA was used to identify significant results, and Dunnett’s procedure was used for multiple comparisons. (*n* = 3). (B) WST-8 assays were used to detect cell viability after 48 h and 96 h of culture with control HT-1376 cells and MMP-14-knockdown HT-1376 cells. Two-way ANOVA was employed to detect significant results, and Bonferroni’s multiple comparison approach was used; (*n* = 3). (C) Cell death detection assays were used to assess cell apoptosis after 48 h and 96 h of culture with control HT-1376 cells and MMP-14-knockdown HT-1376 cells. Two-way ANOVA was employed to detect significant results, and Bonferroni’s multiple comparison approach was used; (*n* = 3). (D) Western blotting showing the expression levels of MMP-14 and PD-L1 in RT-112 control cells and RT-112 MMP-14-overexpressing cells. One-way ANOVA was used to identify significant results, and Dunnett’s procedure was used for multiple comparisons. (*n* = 3). (E) WST-8 assays were used to detect cell viability after 48 h and 96 h of culture with RT-112 control cells and RT-112 MMP-14-overexpressing cells. Two-way ANOVA was employed to detect significant results, and Bonferroni’s multiple comparison approach was used; (*n* = 3). (F) Cell death detection assays were used to assess cell apoptosis after 48 h and 96 h of culture with RT-112 control cells and RT-112 MMP-14-overexpressing cells. Two-way ANOVA was employed to detect significant results, and Bonferroni’s multiple comparison approach was used; (*n* = 3). Control cells were mock-transfected clones. KD: knockdown, OE: overexpression. Data are represented as mean ± SD. ns, not significant; ∗*p* < 0.05, ∗∗*p* < 0.01, and ∗∗∗*p* < 0.001.

### MMP-14 mediates effector T cell exhaustion and associates with Treg enrichment in bladder cancer

Based on the finding that MMP-14 can endogenously upregulate surface PD-L1 on cancer cells, we hypothesized that it plays a key role in remodeling the extrinsic tumor immune microenvironment (TME). Using the single-sample GSEA (ssGSEA) algorithm based on TCGA data, we found a moderate positive correlation between MMP-14 expression and the enrichment of regulatory T cells (Treg cells), the main immunosuppressive cells (Figures 4A and 4B). Furthermore, we queried the TIMER and GEPIA databases to reveal that MMP-14 expression strongly correlated not only with core Treg markers but also with inhibitory checkpoint receptors commonly associated with T cell activation and dysfunction (PD-1, CTLA4, LAG3, and TIM-3) (Table 1). To systematically validate this immune evasion hypothesis *in vitro*, we co-cultured CD3^+^ T cells with the genetically modified cancer cells (Figure 4C). We used a PI-Annexin V assay to evaluate the apoptosis of cancer cells (Figure 4D). The results showed that knocking down MMP-14 in HT-1376 cells significantly enhanced T-cell-induced cancer cell apoptosis. (Figure 4E). Furthermore, MMP-14 knockdown significantly downregulated the expression of TIM-3 and PD-1 and increased the expression levels of granzyme B and perforin in co-cultured CD3^+^ T cells. Consistently, overexpression of MMP-14 in RT-112 cells markedly reduced T-cell-induced apoptosis (Figure 4F), while LAG-3, TIM-3 and PD-1 increased and granzyme B decreased (Figure 4G).

**Figure 4:**
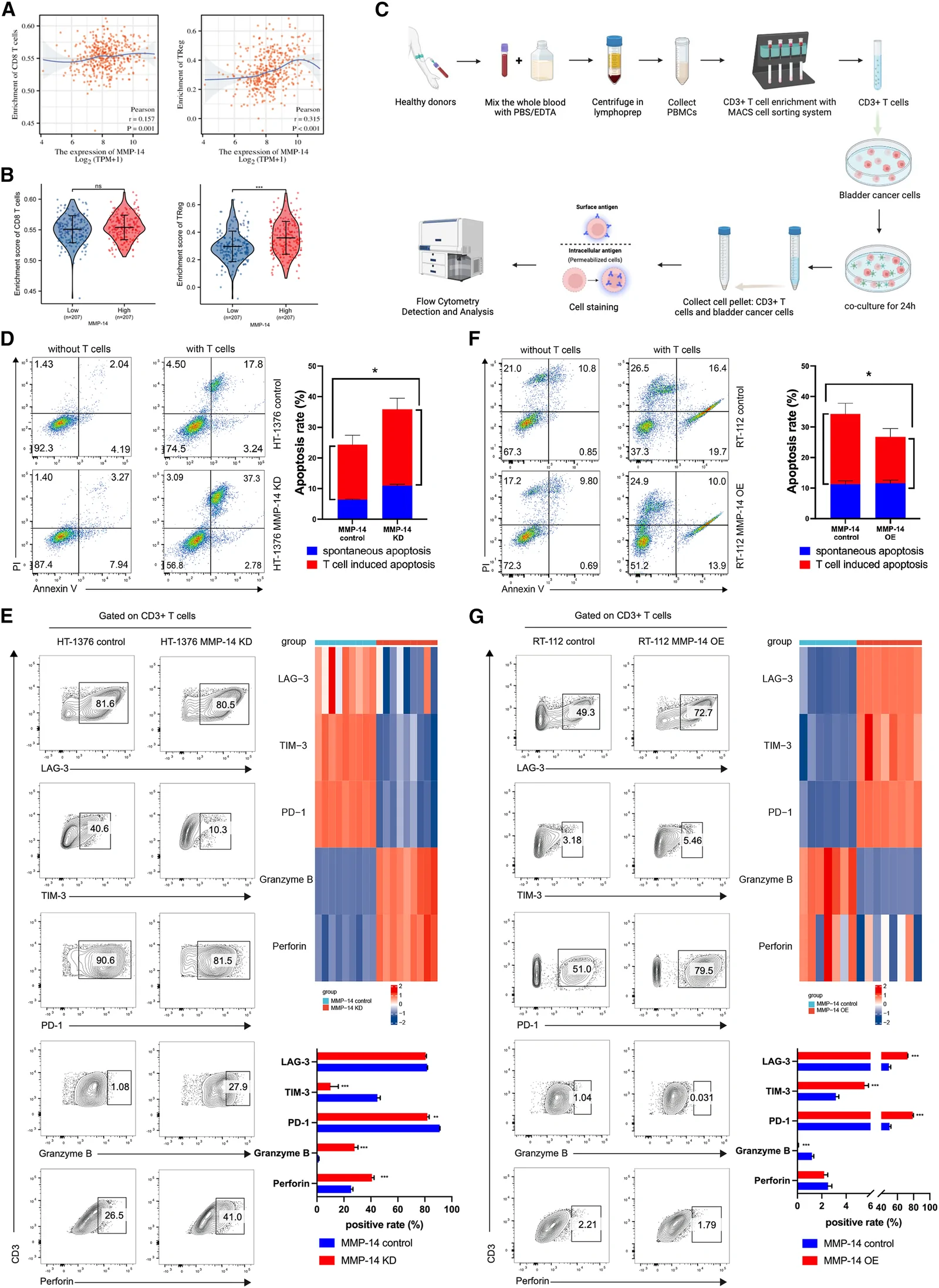
The role of MMP-14 in the immunosuppression of bladder cancer (A) Single sample gene set enrichment analysis (ssGSEA) was used to analyze the correlation between the expression level of MMP-14 and the abundance of CD8^+^ T cells and Treg cells in bladder cancer based on TCGA data. Correlation was analyzed using Pearson correlation analysis. (B) The ssGSEA algorithm was used to determine the degree of enrichment of CD8^+^ T cells and Treg cells in different MMP-14 expression groups in bladder cancer data from the TCGA database. Significant results were determined by the Mann-Whitney U test. (C) Workflow chart for enrichment of CD3^+^ T cells and coculture of bladder cancer cells and CD3^+^ T cells. (D) A PI-Annexin V assay was used to evaluate the apoptosis of cancer cells after 24 h of coculture with CD3^+^ T cells with control HT-1376 cells or MMP-14 knockdown HT-1376 cells. Significant results were determined by an independent *t* test; (*n* = 3). (E) Flow cytometry was used to detect immune markers in CD3^+^ T cells after 24 h of coculture of CD3^+^ T cells with control HT-1376 cells or MMP-14 knockdown HT-1376 cells. Significant results were determined by independent *t* test; (*n* = 3). (F) A PI-Annexin V assay was used to evaluate the apoptosis of cancer cells after 24 h of coculture of CD3^+^ T cells with RT-112 control cells or RT-112 MMP-14-overexpressing cells. Significant results were determined by an independent *t* test; (*n* = 3). (G) Flow cytometry was used to detect immune markers in CD3^+^ T cells after 24 h of coculture of CD3^+^ T cells with RT-112 control cells or RT-112 MMP-14-overexpressing cells. Significant results were determined by independent *t* test; (*n* = 3). Data are represented as mean ± SD. ns, not significant; ∗*p* < 0.05, ∗∗*p* < 0.01, and ∗∗∗*p* < 0.001.

**Table 1:** Correlation analysis between MMP-14 expression and markers of CD8^+^ T cells, Treg cells, and T cell exhaustion in bladder cancer from the TIMER and GEPIA databases

Cell type	Gene marker	TIMER		GEPIA	
		Correlation	*p* value	Correlation	*p* value
CD8^+^ T cells	CD8A	0.068	0.195	0.17	∗∗∗
	CD8B	−0.026	0.614	0.0043	0.93
Treg cells	FOXP3	0.258	∗∗∗	0.34	∗∗∗
	CD25 (IL2RA)	0.341	∗∗∗	0.31	∗∗∗
	CCR8	0.291	∗∗∗	0.31	∗∗∗
T cell exhaustion	PD-1 (PDCD1)	0.124	∗	0.11	∗
	CTLA4	0.171	∗∗∗	0.22	∗∗∗
	LAG3	0.149	∗∗	0.23	∗∗∗
	TIM-3 (HAVCR2)	0.326	∗∗∗	0.38	∗∗∗

∗, ∗∗, and ∗∗∗ indicate *p* < 0.05, *p* < 0.01, and *p* < 0.001, respectively.

### STAT3 serves as an upstream transcription factor positively regulating MMP-14 and PD-L1 expression

Having established the function of the MMP-14/PD-L1 axis in driving immune evasion, we delved into the molecular mechanisms regulating MMP-14. We used the KnockTF database and hTFtarget database for transcription factors that may regulate MMP-14. Among the 11 common transcription factors presented in the Venn diagram (Figure 5A), STAT3, MYC, JUN, FOXM1, GATA1, and BRD4 were also strongly positively correlated with MMP-14 in TCGA bladder cancer data. The correlation coefficient between STAT3 and MMP-14 was the largest, reaching 0.422 (Figure 5B). Given that STAT3 is a known transcriptional activator of PD-L1, a tripartite co-expression analysis confirmed a highly significant positive correlation among MMP-14, STAT3, and PD-L1 (Figure 5C). To experimentally verify the upstream driving role of STAT3 in this cascade, we employed STAT3-specific interventions. We treated the bladder cancer cells with different concentrations of the STAT3-specific inhibitor HO-3867 for 48 hand determined the corresponding IC50 for each cell line (Figure S2A). HO-3867 downregulated the expression levels of p-STAT3 and MMP-14 in all cell lines and of the STAT3 protein in UM-UC-3, RT-4 and HT-1376 cells. With the exception of UM-UC-3 cells, the cell lines exhibited significantly reduced PD-L1 expression (Figure 5D). The inhibitor reduced MMP-14 activity in bladder cancer cells (except for RT-112 cells, Figure 5E) and inhibited cell proliferation (Figure 5F). Consistently, HO-3867 markedly increased the apoptosis index compared with the control, except in RT-4 cells at 24 h (Figure 5G). Cytotoxicity assays revealed the cytotoxic effects of HO-3867 (Figure S2B). To verify that STAT3 regulates MMP-14 in bladder cancer, we used the STAT3-specific activator colivelin to increase phosphorylation of STAT3. The expression levels of (p-)STAT3, MMP-14, and PD-L1 in all cell lines were detected by Western blotting, which showed that colivelin significantly increased the phosphorylation of STAT3 and, interestingly, the expression levels of MMP-14 and PD-L1, too (Figure 5H), thereby confirming the upstream positive regulatory position of STAT3 over MMP-14.

**Figure 5:**
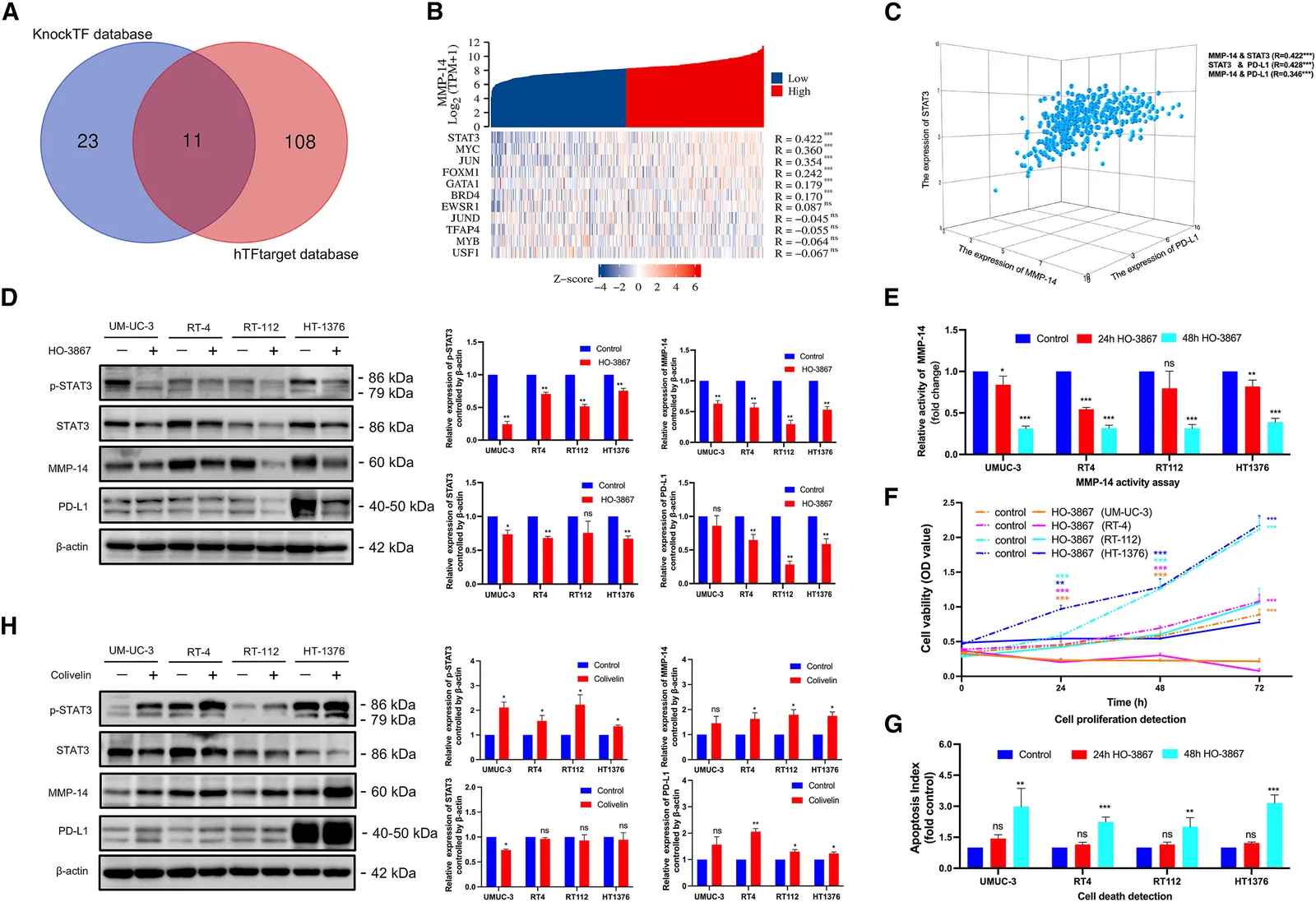
The function of STAT3 in the context of MMP-14 and PD-L1 in bladder cancer cells (A) Venn diagram of the screened public transcription factors of MMP-14 in the KnockTF database and hTFtarget database. (B) Expression heatmap shows the coexpression of MMP-14 and public transcription factors in the TCGA database. Correlation was analyzed using Pearson’s correlation analysis. (C) The 3D scatterplot correlating MMP-14, STAT3, and PD-L1 as correlated genes (CGs) in the TCGA database. Correlation was analyzed by Pearson correlation analysis. (D) Western blotting was used to detect changes in the expression of p-STAT3, STAT3, MMP-14, and PD-L1 in bladder cancer cells after 24 h of treatment with HO-3867. STAT3 ranged from 86 kDa (STAT3α, the predominant form) to 79 kDa (STAT3β). Significant results were determined by an independent *t* test; (*n* = 3). (E) MMP-14 activity assays were used to detect the MMP-14 activity of bladder cancer cells after the cells were treated with HO-3867 for 24 h or 48 h. One-way ANOVA was used to identify significant results, and Dunnett’s procedure was used for multiple comparisons. (*n* = 3). (F) WST-8 assays were used to measure cell viability after 24 h, 48 h, and 72 h of HO-3867 treatment. Two-way ANOVA was employed to detect significant results, and Bonferroni’s multiple comparison approach was used; (*n* = 3). (G) A cell death detection assay was used to assess the apoptosis of bladder cancer cells after 24 h and 48 h of HO-3867 treatment. Two-way ANOVA was employed to detect significant results, and Bonferroni’s multiple comparison approach was used; (*n* = 3). (H) Western blotting was used to detect changes in the expression of p-STAT3, STAT3, MMP-14, and PD-L1 in bladder cancer cells after 2 h of colivelin treatment. The cells were precultured with serum-free medium 24 h before colivelin treatment. One-way ANOVA was used to identify significant results, and Dunnett’s procedure was used for multiple comparisons. (*n* = 3). Data are represented as mean ± SD. ns, not significant; ∗*p* < 0.05, ∗∗*p* < 0.01, and ∗∗∗*p* < 0.001.

### MMP-14 induces STAT3 phosphorylation and activation through direct physical interaction

Although the above results confirmed STAT3 as an upstream regulator of MMP-14, pathway enrichment analysis suggested that MMP-14 is concurrently involved in modulating downstream JAK-STAT signaling (Figure S3), hinting at a reciprocal feedback mechanism. Strikingly, a human phosphorylated kinase antibody array revealed that pharmacological inhibition of MMP-14 caused a precipitous drop in STAT3 phosphorylation (S727 and Y705 sites) in HT-1376 cells (Figure 6A; Table S5). Further studies will be needed to validate these data but phosphorylation at Y705 was also shown by the Y705-specific phospho-STAT3 antibody we used in our blots (Figures 5, 6, and 7; key resources table). Western blotting showed that NSC405020 decreased the expression levels of p-STAT3 and STAT3 in bladder cancer cells (Figure 6B). The expression levels of p-STAT3 and STAT3 were also markedly lower in the MMP-14 KD clones than in the control (Figure 6C), whereas MMP-14 overexpression significantly increased (p-)STAT3 in OE3/OE4, and STAT3 in all clones except OE2 (Figure 6D). To explore the mechanism by which MMP-14 regulates PD-L1 and STAT3, we used coimmunoprecipitation and coimmunoprecipitation combined with silver staining (Figure S4, potentially relevant bands were excised) to identify proteins that bound to MMP-14 in HT-1376 cells by mass spectrometry. We applied the following exclusion criteria to potential MMP-14 binding partners: negative difference between MMP-14 IP and control IP; contaminating proteins; structural proteins, e.g., keratins, ribosomal proteins, histones, actins, and myosins; unique peptides ≤2; and targets occurring in each of the 6 gel bands. Tables S6 and S7 contain the datasets, and Figure 6E presents the relevant proteins of the three whole-lysate precipitation experiments. Interesting proteins were selected and verified using coimmunoprecipitation and Western blotting (Figure S5). The results showed that MMP-14 interacted with E-cadherin (CDH1), β-catenin (CTNNB1), integrin β4 (ITGB4), insulin-like growth factor 2 mRNA-binding protein 3 (IMP2/IGF2BP3), and Y-box binding protein (YBX1) in HT-1376 cells. Of these, only β-catenin has been shown to directly bind MMP-14 in MDCK normal kidney cells of a dog.^19^ Notably, we did not observe any direct interaction of MMP-14 with PD-L1 in HT-1376 cells (Figure 6F), but we did observe this interaction with STAT3 (Figures 6E and 6G). Because STAT3 is strictly an intracellular transcription factor and the catalytic domain of MMP-14 resides exclusively in the extracellular space, this direct physical interaction is topologically required to occur via non-proteolytic protein-protein interactions mediated by the 20-amino acid intracellular cytoplasmic tail of MMP-14. We used coimmunoprecipitation with an MMP-14 antibody to verify that MMP-14 could interact with STAT3 in HT-1376 and RT-112 cells (Figure 6G). Similarly, using a STAT3 antibody for precipitation, we also found that STAT3 could interact with MMP-14 in both cell lines (Figure 6H). STAT3 bound to MMP-14 included phosphorylated STAT3 as well, shown for HT-1376 by use of the Y705-specific phospho-STAT3 antibody (Figure S5B), suggesting a potential structural basis by which MMP-14 might maintain STAT3 activity.

**Figure 6:**
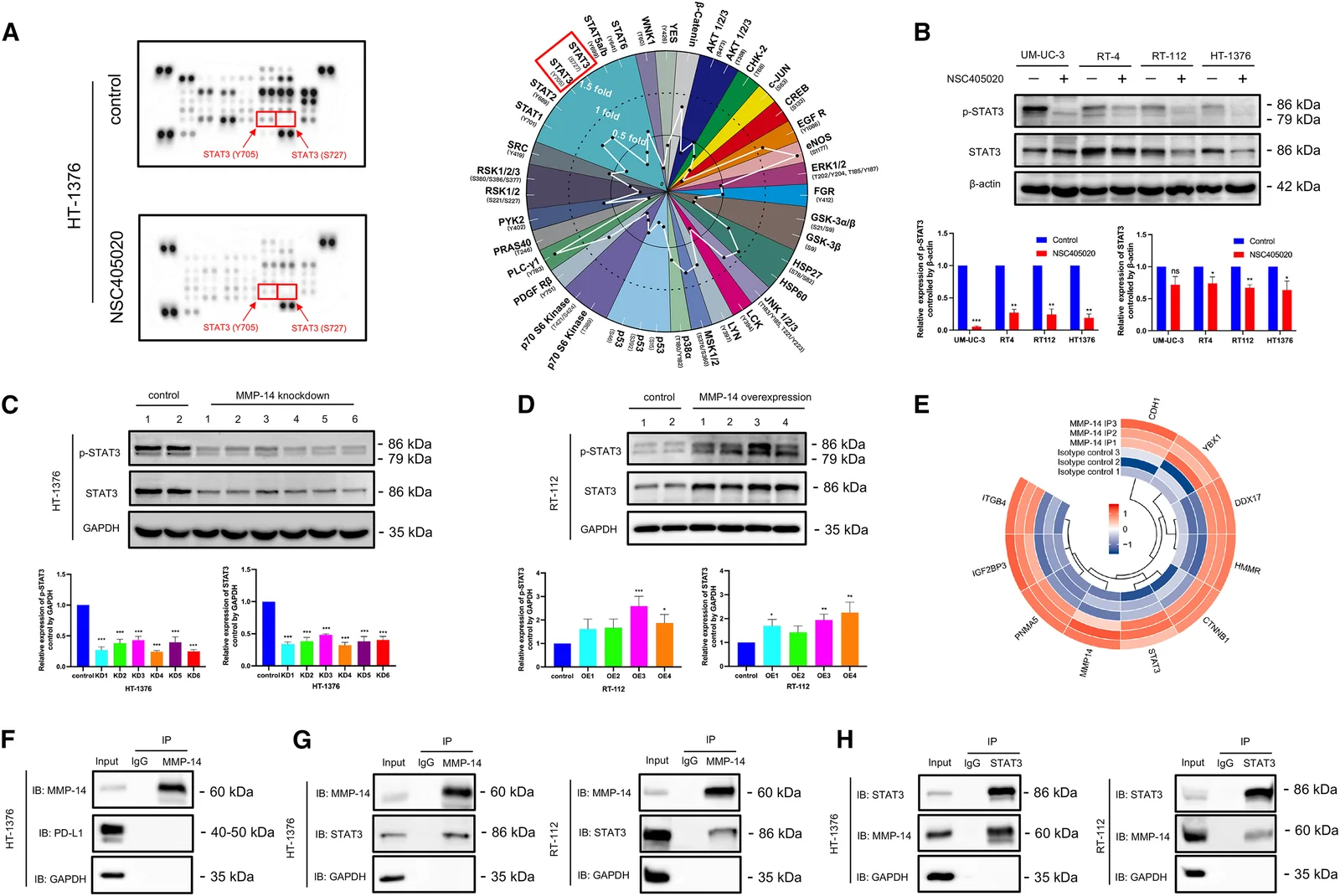
The regulation of STAT3 by MMP-14 in bladder cancer (A) A human phosphorylated kinase antibody array was used to detect changes in the expression of phosphorylated kinases after HT-1376 cells were treated with NSC405020 for 24 h. Fold changes relative to the control are shown in the radar chart. (B) Western blotting was used to detect changes in the expression of p-STAT3 and STAT3 after treatment of bladder cancer cells with NSC405020 for 24 h. Significant results were determined by an independent *t* test; (*n* = 3). (C) Western blotting showing the expression levels of p-STAT3 and STAT3 in control HT-1376 cells and MMP-14 knockdown HT-1376 cells. One-way ANOVA was used to identify significant results, and Dunnett’s procedure was used for multiple comparisons. (*n* = 3). (D) Western blotting showing the expression levels of p-STAT3 and STAT3 in RT-112 control cells and RT-112 MMP-14-overexpressing cells. One-way ANOVA was used to identify significant results, and Dunnett’s procedure was used for multiple comparisons. (*n* = 3). (E) Circular heatmap showing proteins that may directly interact with MMP-14 according to mass spectrometry analysis of 3 HT-1376 cell coimmunoprecipitation experiments. (F) Coimmunoprecipitation with an MMP-14 antibody and Western blotting with an anti-PD-L1 antibody did not detect any direct interaction between MMP-14 and PD-L1 in HT-1376 cells. (G) Coimmunoprecipitation with an MMP-14 antibody and Western blotting were used to verify that MMP-14 could directly interact with STAT3 in RT-112 and HT-1376 cells. (H) Vice versa, coimmunoprecipitation with a STAT3 antibody and Western blotting were used to verify that STAT3 could directly interact with MMP-14 in RT-112 and HT-1376 cells. Data are represented as mean ± SD. ns, not significant; ∗*p* < 0.05, ∗∗*p* < 0.01, and ∗∗∗*p* < 0.001.

**Figure 7:**
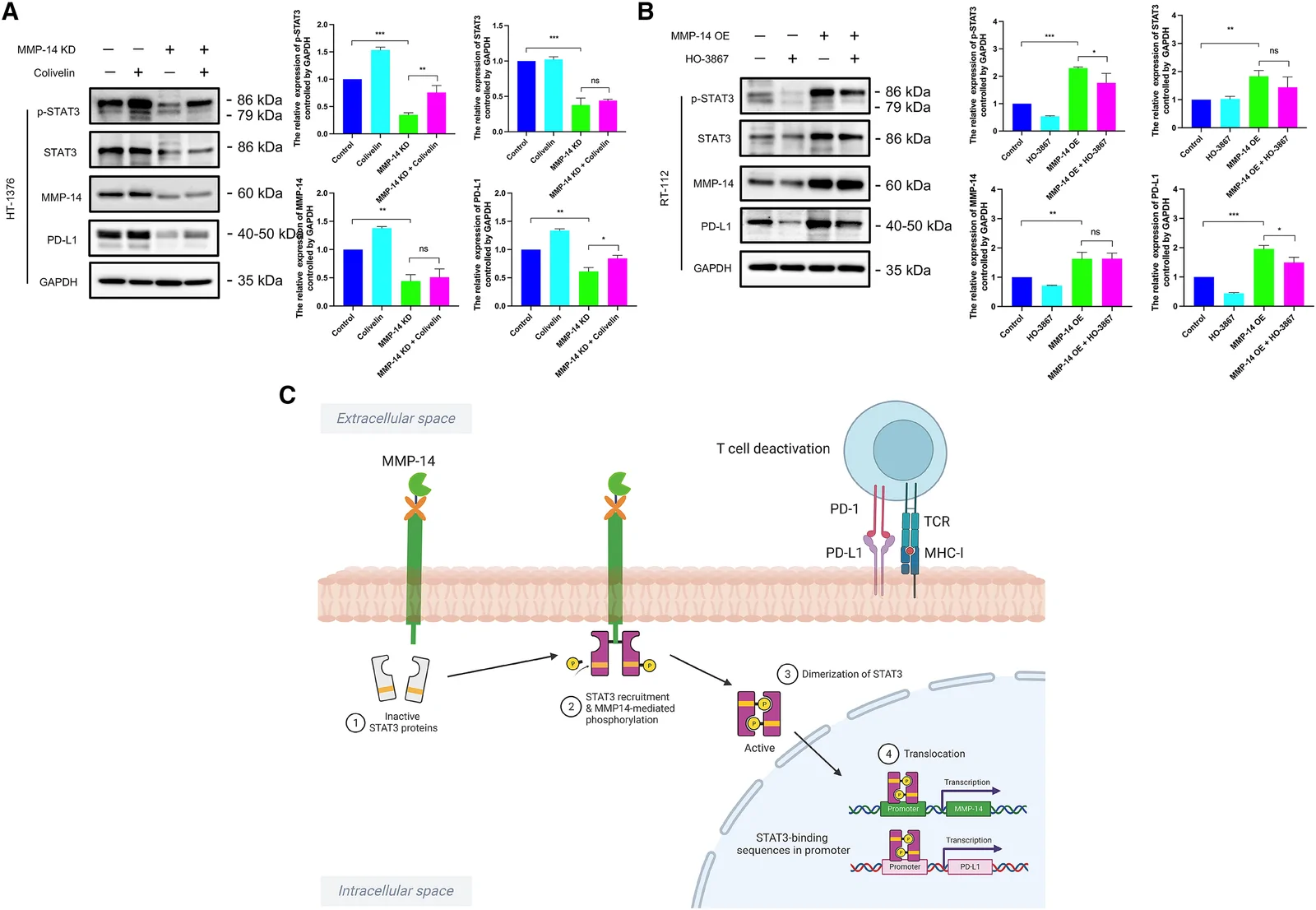
Possible connections between MMP-14, STAT3 and PD-L1 in bladder cancer (A) Western blotting showing the expression levels of p-STAT3, STAT3, MMP-14 and PD-L1 in HT-1376 control cells and in HT-1376 MMP-14 knockdown cells with or without colivelin treatment. One-way ANOVA was used to identify significant results, and Dunnett’s procedure was used for multiple comparisons. (*n* = 3). (B) Western blotting was used to detect the expression levels of p-STAT3, STAT3, MMP-14, and PD-L1 in RT-112 control cells and RT-112 MMP-14-overexpressing cells with or without HO-3867 treatment. GAPDH served as a control. One-way ANOVA was used to identify significant results, and Dunnett’s procedure was used for multiple comparisons. (*n* = 3). (C) A model for the regulatory mechanism among MMP-14, STAT3, and PD-L1 in bladder cancer cells. Data are represented as mean ± SD. ns, not significant; ∗*p* < 0.05, ∗∗*p* < 0.01, and ∗∗∗*p* < 0.001.

### The MMP-14/STAT3 positive feedback loop drives a mechanistic model of PD-L1-mediated immune evasion

To further investigate the hypothesis that MMP-14 modulates PD-L1 expression by regulating STAT3 activity, we designed critical signal rescue experiments. Introducing the STAT3 activator colivelin into HT-1376 MMP-14 KD cells successfully bypassed the MMP-14 deficiency, partially rescuing the expression of p-STAT3 and PD-L1 without affecting the baseline levels of MMP-14 (Figure 7A). In a reverse approach, applying the STAT3 inhibitor HO-3867 to RT-112 MMP-14 OE cells partially reversed the high levels of p-STAT3 and PD-L1 artificially induced by MMP-14 overexpression (Figure 7B). Based on our systematic findings spanning from clinical data to molecular interactions, we propose a novel mechanistic model for cancer immune evasion: MMP-14 and STAT3 physically interact intracellularly to maintain and promote STAT3 phosphorylation; this continuously activates STAT3, acting as a transcription factor, reciprocally drives the transcription of both MMP-14 itself and the key immune checkpoint PD-L1. It is hypothesized that this closed positive feedback loop not only promotes the malignant proliferation of bladder cancer cells but could also contribute to an immunosuppressive barrier by inducing acute functional suppression in the microenvironment (Figure 7C).

## Discussion

The current investigation elucidates a critical and previously undercharacterized role for MMP-14 in bladder cancer progression and immune evasion. Rather than functioning solely as an extracellular matrix (ECM) remodeling enzyme, the data suggest that MMP-14 acts as a central signaling hub. The findings define a tripartite mechanism: firstly, MMP-14 drives tumor proliferation and apoptosis evasion; secondly, it orchestrates immunosuppression and PD-L1 regulation; what’s more, these processes are intrinsically governed by a self-sustaining positive feedback loop involving direct interaction with STAT3.

### MMP-14 drives tumorigenesis and apoptosis evasion in bladder cancer cells

Although MMP-14 is classically recognized to dictate 3D invasive growth rather than robust 2D proliferation,^20^ it was observed that targeted inhibition with NSC405020 or stable knockdown of MMP-14 modestly slowed 2D proliferation while profoundly increasing apoptosis of bladder cancer cells, with reciprocal effects observed in MMP-14-overexpressing clones. This phenomenon is likely mediated by the circumvention of collagen-induced apoptosis. In standard physiological states, intact type I collagen binds to the Discoidin Domain Receptor 1 (DDR1), which subsequently triggers BIK-mediated apoptosis to restrict abnormal cellular growth.^20^^,^^21^ High levels of MT1-MMP rapidly degrade the structural integrity of this collagen lattice, preventing proper DDR1 activation.^20^ It is hypothesized that this structural ablation may silence the apoptotic cascade, potentially facilitating tumor cell proliferative capacity.

### MMP-14 orchestrates immunosuppression and PD-L1 regulation in bladder cancer cells

A central finding of this study is the potent immunosuppressive capability of MMP-14. Our results demonstrated that the MMP-14/STAT3/PD-L1 axis exerts profound, differential effects across distinct T cell subpopulations, establishing a multipronged mechanism of immune evasion. In the CD8^+^ cytotoxic T cell compartment, the STAT3-driven upregulation of PD-L1 directly engages PD-1 to induce rapid functional suppression, reducing the expression of critical cytotoxic proteins, which may override potential activating effects of MMP-14-mediated CD100 shedding.^22^ Concurrently, this axis aggressively shapes the CD4^+^ compartment. Tumor-expressed PD-L1 actively promotes the conversion of naive CD4^+^ T cells into induced Tregs (iTregs) by dampening the Akt/mTOR signaling cascade.^23^ Furthermore, MMP-14 directly augments Treg expansion through the proteolytic cleavage of the latency-associated peptide (LAP), releasing active TGF-β from the integrin αvβ8 complex.^24^ Thus, the observed weakening of T cell killing in our co-culture system is the result of a synergistic microenvironment where profound CD8^+^ functional suppression is enforced by rapid, MMP-14/PD-L1-driven Treg amplification and conversion.^23^^,^^24^ It is imperative to contextualize these findings within the broader tumor immune microenvironment (TIME). High MMP-14 expression is also linked to the recruitment of intensely immunosuppressive M2-type tumor-associated macrophages (TAMs),^25^ and its proteolytic cleavage of surface receptors like DR6 can severely hinder dendritic cell maturation.^26^ Thus, the novel intracellular cascade operates in synergy with its extracellular functions.

### A non-proteolytic positive feedback mechanism between MMP-14 and STAT3

The mechanisms governing the co-regulation of MMP-14 and PD-L1 are anchored by STAT3. Vice versa, our study showed that STAT3 was a downstream target of MMP-14 in bladder cancer cells.^27^^,^^28^ Because the allosteric inhibitor NSC405020—which exclusively targets the PEX domain to abolish homodimerization without disrupting intrinsic catalytic activity—effectively reduced p-STAT3 levels, we conclude that the regulation of STAT3 relies strictly on non-proteolytic protein-protein interactions rather than enzymatic cleavage.^29^ Furthermore, due to the topological segregation of the extracellular catalytic domain and intracellular STAT3, our co-immunoprecipitation data demonstrating a direct physical interaction between MMP-14 and (phospho-)STAT3 indicates that this mechanism might operate via the 20-amino acid cytoplasmic tail of MMP-14.^27^^,^^30^ However, since MMP-14 is a metalloprotease lacking an intrinsic kinase domain, it cannot directly catalyze STAT3 Y705 phosphorylation. We hypothesize that the short cytoplasmic tail of MMP-14 functions as a non-proteolytic molecular scaffold that recruits a non-receptor tyrosine kinase—such as Src or JAK2—into the complex.^28^^,^^31^ Spatially, this robust interaction likely occurs on the cytoplasmic surface of early endosomes following rapid MMP-14 internalization, creating a dynamic signaling platform.^32^ By bringing STAT3 into spatial proximity with recruited kinases like Src or JAK2, the MMP-14 scaffold facilitates specific Y705 phosphorylation. Once phosphorylated, p-STAT3 dimerizes and translocates to the nucleus to drive the transcription of CD274 (PD-L1), resulting in T cell functional suppression.^33^^,^^34^^,^^35^ Concurrently, p-STAT3 acts as a direct transcription factor for MMP-14 itself.^36^ This generates an aggressive, self-amplifying positive feedback loop: baseline MMP-14 structurally facilitates STAT3 phosphorylation^35^^,^^36^; active p-STAT3 drives the expression of both PD-L1 and further MMP-14; and the elevated membrane density of MMP-14 perpetuates continuous STAT3 activation.

While our primary mechanistic focus highlighted STAT3 due to the definitive physical interaction with MMP-14 established in our assays, it is imperative to acknowledge that the human phospho-kinase array illuminated a much broader signaling cascade. Specifically, precipitous decreases were observed in the phosphorylation of CREB (S133), PRAS40 (T246), WNK1 (T60), RSK1/2 (S221/S227), GSK-3β (S9), and p70 S6 Kinase upon MMP-14 inhibition. These extensively downregulated pathways are profoundly intertwined with the global architecture of tumor immunosuppression; for instance, hyperactivation of GSK-3β (indicated by reduced S9 phosphorylation) precipitates the β-TrCP-mediated proteasomal degradation of PD-L1,^37^ while the dephosphorylation of PRAS40/p70 S6K delineates a severe blockade of mTORC1-mediated ribosomal translation of PD-L1 mRNA.^38^ Therefore, MMP-14 inhibition dismantles bladder cancer immune evasion not through a singular pathway, but rather by forcing a catastrophic, multi-tiered collapse of PD-L1 synthesis via redundant transcriptional, translational, and proteolytic degradation mechanisms.

These very important findings establish MMP-14 as a critical mediator of immune evasion. Importantly, while our current study delineates the molecular establishment of this axis, the functional restoration of T cell cytotoxicity through downstream interventions presents a compelling clinical avenue. Based on the mechanistic architecture we identified, the application of STAT3 inhibitors (such as HO-3867) or PD-L1 neutralizing antibodies in MMP-14-overexpressing environments possesses the strong theoretical and practical potential to break this positive feedback loop and effectively restore CD8^+^ T cell cytotoxicity, as corroborated by recent functional rescue models targeting the STAT3/PD-L1 axis.^35^^,^^39^ This opens a new and highly promising field of research to combine immune checkpoint blockade with targeted MMP-14 or STAT3 inhibitors for advanced bladder cancer therapy.^13^^,^^35^

### Limitations of the study

There are several limitations to our study that warrant explicit discussion. First, while our bioinformatic analytical strategy applying the intersection of DEGs and CGs minimized false-positive rates, standard correlation metrics are sensitive primarily to linear dependencies and may overlook biologically important non-linear relationships.^40^^,^^41^ Additionally, because some TCGA subgroups were small, our clinical associations are exploratory. However, the reliability of our conclusions is profoundly reinforced by recent independent clinical cohort studies. Wang et al. and Zhang et al. recently validated through comprehensive immunohistochemistry on totally independent real-world bladder cancer cohorts (*n* = 113 and *n* = 68, respectively) that high MMP-14 protein expression is significantly associated with advanced muscle-invasive stages, poor prognosis, and a markedly reduced response to immunotherapy.^9^^,^^42^ Furthermore, Civriz et al. corroborated the significant spatial correlation between STAT3 and PD-L1 positivity in clinical tissue specimens.^43^

Second, our functional conclusions were drawn from human *in vitro* co-culture experiments. We deliberately refrained from deploying murine models (such as MB49 or BBN-induced models) due to profound species-specific evolutionary divergences. Human PD-1 possesses a lineage-specific pre-ITSM PEQ motif—absent in rodents—that strongly recruits Shp2 to drive T cell exhaustion, meaning murine models severely underestimate human immunosuppression.^18^ Furthermore, the widely used MB49 syngeneic model naturally exhibits a loss of the Y chromosome (LOY), driving profound baseline CD8^+^ T cell exhaustion and creating an extreme, artificial immune evasion phenotype.^44^

Third, our evaluation of immune killing utilized bulk CD3^+^ T cells; this might dilute the measurement of specific CD8^+^ cytotoxic capacity and complicate the interpretation of exhaustion markers across CD4^+^, CD8^+^ and Treg subsets.^45^

Finally, our current study relied on the acute pharmacological modulation of STAT3 using HO-3867 and colivelin. While the target specificity of these small molecules has been robustly validated (e.g., phenotypic rescue via STAT3 cDNA overexpression and functional ablation via dominant-negative constructs), complementing our pharmacological data with parallel transient STAT3 genetic knockdown directly in our specific bladder cancer models is essential to completely exclude potential non-specific cytotoxicity.^46^^,^^47^ Future validation of this MMP-14/STAT3 axis should prioritize advanced 3D patient-derived organoids (PDOs) co-cultured with autologous tumor-infiltrating lymphocytes (TILs) or multiplexed spatial transcriptomics on human bladder cancer tissues.

In conclusion, disrupting the MMP-14/STAT3 axis may have multifaceted clinical benefits. *In vitro* analyses suggest that targeted inhibition attenuates proliferative signaling and downregulates PD-L1. Furthermore, bioinformatic data raise the hypothesis that MMP-14 may influence Treg enrichment, although direct confirmation of TGF-β activation requires *in vivo* investigation. MMP-14 thus emerges as a highly promising target to reverse immune resistance and enhance the efficacy of combined immunotherapies in advanced bladder cancer. Furthermore, our discovery of putative MMP-14 binding partners, including E-cadherin, integrin beta-4, IGF2BP3, YBX1 and beta-catenin, provides the basis for novel research approaches.

## Resource availability

### Lead contact

Further information and requests for resources and reagents should be directed to and will be fulfilled by the lead contact, Shuai Zhu (shuaizhu.med@qq.com).

### Materials availability

This study did not generate new unique reagents.

### Data and code availability

• This paper analyzes existing, publicly available data (TCGA-BLCA), accessible at the Genomic Data Commons: https://portal.gdc.cancer.gov. All other data reported in this paper will be shared by the lead contact upon reasonable request.
• This paper does not report original code.
• Any additional information required to reanalyze the data reported in this paper is available from the lead contact upon reasonable request.

## Acknowledgments

The results shown here are in part based upon data generated by the TCGA Research Network: https://www.cancer.gov/tcga. We thank the investigators and research groups of the TCGA data sets who participated in the collection of specimens and shared the information publicly. Many thanks also to the Flow Cytometry Core Facility of Charité - Universitätsmedizin Berlin for providing us with flow cytometry equipment and to Beata Lukaszewska-McGreal for technical assistance in mass spectrometry. This study received financial support from the Foundation for Urological Research Berlin (S.Z., L.K.) and the 10.13039/501100004543China Scholarship Council (S.Z., no. 202006370028).

## Author contributions

Conceptualization, S.Z. and A.R.; methodology, S.Z., L.K., and A.R.; validation, S.Z.; formal analysis, S.Z., B.E., and D.M.; investigation, S.Z., L.K., B.E., and D.M.; data curation, S.Z. and D.M.; writing – original draft, S.Z.; writing – review and editing, D.M., J.B., and A.R.; visualization, S.Z. and L.K.; resources, A.R.; supervision, J.B. and A.R.; project administration, A.R.; funding acquisition, S.Z. and A.R. All the authors have read and approved the final manuscript.

## Declaration of interests

The authors declare no competing interests.

## Declaration of generative AI and AI-assisted technologies in the manuscript preparation process

During the preparation of this work, the authors used ChatGPT to improve readability and check grammar; the authors reviewed and edited the content as needed and take full responsibility for the content of the publication.

## STAR★Methods

### Key resources table

**Table:** 

REAGENT or RESOURCE	SOURCE	IDENTIFIER
**Antibodies**		

Rabbit monoclonal anti-MMP-14 (clone EP1264Y)	Abcam	Cat#ab51074; RRID: AB_881234
Rabbit monoclonal anti-PD-L1 (clone E1L3N)	Cell Signaling Technology	Cat#13684; RRID: AB_2687655
Rabbit monoclonal anti-phospho-STAT3 (Tyr705) (clone D3A7)	Cell Signaling Technology	Cat#9145; RRID: AB_2491009
Mouse monoclonal anti-STAT3 (clone 124H6)	Cell Signaling Technology	Cat#9139; RRID: AB_331757
Rabbit monoclonal anti-E-cadherin (clone 24E10)	Cell Signaling Technology	Cat#3195; RRID: AB_2291471
Rabbit monoclonal anti-β-catenin (clone D10A8)	Cell Signaling Technology	Cat#8480; RRID: AB_11127855
Rabbit monoclonal anti-integrin β4 (clone D8P6C)	Cell Signaling Technology	Cat#14803; RRID: AB_2798620
Rabbit monoclonal anti-IGF2BP3/IMP3 (clone D6U2N)	Cell Signaling Technology	Cat#57145; RRID:AB_2799525
Rabbit monoclonal anti-YB1 (clone D2A11)	Cell Signaling Technology	Cat#9744; RRID: AB_11178953
Rabbit monoclonal anti-GATA3 (clone D13C9)	Cell Signaling Technology	Cat#5852; RRID: AB_10835690
Rabbit monoclonal anti-β-actin (clone D6A8)	Cell Signaling Technology	Cat#8457; RRID: AB_10950489
Rabbit monoclonal anti-GAPDH (clone D16H11)	Cell Signaling Technology	Cat#5174; RRID: AB_10622025
Rabbit (DA1E) mAb IgG XP isotype control	Cell Signaling Technology	Cat#3900; RRID: AB_1550038
Mouse (E5Y6Q) mAb IgG2a isotype control	Cell Signaling Technology	Cat#61656; RRID: AB_2799613
Anti-rabbit IgG, HRP-linked antibody	Cell Signaling Technology	Cat#7074; RRID: AB_2099233
Anti-mouse IgG, HRP-linked antibody	Cell Signaling Technology	Cat#7076; RRID: AB_330924
Goat anti-rabbit IgG (H + L), Alexa Fluor 594	Invitrogen	Cat#A-11072; RRID: AB_2534116
APC/Cyanine7 anti-human CD3 (clone HIT3a)	BioLegend	Cat#300318; RRID: AB_314056
PE anti-human CD279 (PD-1) (clone NAT105)	BioLegend	Cat#367404; RRID: AB_2616740
APC anti-human CD366 (TIM-3) (clone A18087E)	BioLegend	Cat#364804; RRID: AB_2910409
Brilliant Violet 711 anti-human CD223 (LAG-3) (clone 11C3C65)	BioLegend	Cat#369320; RRID: AB_2716125
PE/Cyanine7 anti-human perforin (clone dG9)	BioLegend	Cat#308126; RRID: AB_2572049
Pacific Blue anti-human/mouse granzyme B (clone GB11)	BioLegend	Cat#515408; RRID: AB_2562196
Functional grade anti-human CD3 (clone OKT3)	eBioscience/Invitrogen	Cat#16-0037-85; RRID: AB_468855
Ultra-LEAF purified anti-human CD28 (clone CD28.2)	BioLegend	Cat#302934; RRID: AB_11148949

**Biological samples**		

Fresh human peripheral blood from healthy volunteer donors (for PBMC isolation and CD3^+^ T cell enrichment)	This paper (healthy volunteer donors)	N/A

**Chemicals, peptides, and recombinant proteins**		

HO-3867 (STAT3 inhibitor)	Selleck Chemicals	Cat#S7501; CAS: 1172133-28-6
NSC405020 (MMP-14 inhibitor)	Selleck Chemicals	Cat#S8072; CAS: 7497-07-6
Colivelin (STAT3 activator)	Tocris	Cat#3945; CAS: 867021-83-8
Recombinant human IL-2 (premium grade)	Miltenyi Biotec	Cat#130-097-748
Recombinant human IL-7	CellGenix	Cat#1410-050
Recombinant human IL-15	CellGenix	Cat#1413-50
G418 sulfate (Geneticin)	Thermo Fisher Scientific	Cat#10131027
Bisbenzimide (Hoechst 33258)	Merck	Cat#B2261

**Critical commercial assays**		

Colorimetric Cell Viability Kit I (WST-8)	PromoKine	Cat#PK-CA705-CK04
Cell Death Detection ELISA PLUS	Roche	Cat#11774425001
Cytotoxicity Detection Kit PLUS (LDH)	Roche	Cat#04744926001
Human MMP-14 activity assay kit	QuickZyme	Cat#QZBMMP-14H
Human Phospho-Kinase Array Kit	R&D Systems	Cat#ARY003C
Annexin V-FITC Apoptosis Detection Kit	Sigma-Aldrich	Cat#APOAF
BD Cytofix/Cytoperm Fixation/Permeabilization Kit	BD Biosciences	Cat#554714
Pierce Crosslink Magnetic IP/Co-IP Kit	Thermo Fisher Scientific	Cat#88805
Pierce MS-Compatible Magnetic IP Kit	Thermo Fisher Scientific	Cat#90409
Pierce Silver Stain Kit	Thermo Fisher Scientific	Cat#24612
Lymphoprep density gradient medium	STEMCELL Technologies	Cat#07801
SepMate-50 (IVD)	STEMCELL Technologies	Cat#85450
CD3 MicroBeads, human	Miltenyi Biotec	Cat#130-050-101
Protease inhibitor cocktail	Cell Signaling Technology	Cat#5871
PhosSTOP phosphatase inhibitor cocktail	Roche	Cat#04906837001
Enhanced chemiluminescence (ECL) reagent	Merck	Cat#WBLUF0100

**Deposited data**		

TCGA-BLCA transcriptomic and clinical data	The Cancer Genome Atlas (NCI)	https://portal.gdc.cancer.gov/
Supplemental data (Tables S1, S2, S3, S4, S5, S6, and S7)	This paper	Document S1; Tables S2, S3, S4, S5, and S7 as separate supplemental information

**Experimental models: Cell lines**		

Human: RT-4 urinary bladder transitional carcinoma cell line	ATCC	Cat#HTB-2; RRID: CVCL_0036
Human: UM-UC-3 urinary bladder transitional carcinoma cell line	ATCC	Cat#CRL-1749; RRID: CVCL_1783
Human: HT-1376 urinary bladder transitional carcinoma cell line	ATCC	Cat#CRL-1472; RRID: CVCL_1292
Human: RT-112 urinary bladder transitional carcinoma cell line	DSMZ	ACC 418; RRID: CVCL_1670
Human: HT-1376 cells with stable MMP-14 knockdown (clones generated with HCP302442-CG04-3-10-GC)	This paper	N/A
Human: RT-112 cells stably overexpressing MMP-14 (clones generated with EX-A6431-M61-GC)	This paper	N/A

**Recombinant DNA**		

pEZ-M61-MMP14 overexpression plasmid (EX-A6431-M61-GC)	GeneCopoeia (via Biocat)	Cat#EX-A6431-M61-GC
pReceiver-M61 empty control vector (EX-NEG-M61-GC)	GeneCopoeia (via Biocat)	Cat#EX-NEG-M61-GC
pCRISPR-CG04 sgRNA/Cas9 plasmids targeting MMP-14 (HCP302442-CG04-3-10-GC)	GeneCopoeia (via Biocat)	Cat#HCP302442-CG04-3-10-GC
pCRISPR-CG04 scramble sgRNA control (CCPCTR01-CG04-10-GC)	GeneCopoeia (via Biocat)	Cat#CCPCTR01-CG04-10-GC

**Software and algorithms**		

ImageJ	NIH	https://imagej.nih.gov/ij/
FlowJo v10.4	FlowJo LLC	https://www.flowjo.com
BD FACSDiva v8.0	BD Biosciences	https://www.bdbiosciences.com
SPSS v26.0	IBM	https://www.ibm.com/products/spss-statistics
R v3.6.3	R Foundation	https://www.r-project.org
GSVA (ssGSEA)	Bioconductor	https://bioconductor.org/packages/release/bioc/html/GSVA.html
GSEA	Broad Institute	https://www.gsea-msigdb.org/gsea/index.jsp
TIMER	TIMER web server	https://cistrome.shinyapps.io/timer/
GEPIA	GEPIA web server	https://gepia.cancer-pku.cn/
KnockTF	KnockTF database	https://www.licpathway.net/KnockTF/
hTFtarget	hTFtarget database	https://bioinfo.life.hust.edu.cn/hTFtarget/

**Other**		

EndoFectin Max transfection reagent	GeneCopoeia (via Biocat)	Cat#EF013-GC
BD LSRFortessa cell analyzer	BD Biosciences	RRID:SCR_018655
Fixable viability dye (Live/Dead)	Thermo Fisher Scientific	Cat# L23105
Fluorescence mounting medium	Dako	Cat#F4680
8-well RS glass slides	Thermo Fisher Scientific	Cat#154534

### Experimental model and study participant details

#### Cell lines

Human urinary bladder transitional carcinoma cell lines were obtained from the American Type Culture Collection (ATCC; RT-4, Cat#HTB-2; UM-UC-3, Cat#CRL-1749, both male; HT-1376, Cat#CRL-1472, female) and from the German Collection of Microorganisms and Cell Cultures (DSMZ; RT-112, ACC 418, female). RT-4 and RT-112 cells were cultured in RPMI medium (Gibco/Invitrogen, USA). UM-UC-3 and HT-1376 cells were cultured in MEM Eagle's medium (Biochrom, Germany) with supplements. All cells got 10% fetal bovine serum (20% for HT-1376, Biochrom), penicillin (100 U/mL), streptomycin (100 μg/mL, both Applichem, Germany) and were grown at 37°C and 5% CO_2_. All cell lines were recently authenticated and tested negative for mycoplasma by Multiplexion (Germany).

#### Human peripheral blood samples

Fresh human peripheral blood from healthy volunteer donors was used for the isolation of peripheral blood mononuclear cells (PBMCs) and the enrichment of CD3^+^ T cells, as detailed in the method details. Donor sex and age were not recorded and were therefore not analyzed as biological variables in this study.

#### Ethics statement

Our study exclusively used publicly available, de-identified TCGA data according to TCGA's policies. TCGA data are in accordance with the Declaration of Helsinki. Its use for secondary research does not require additional Institutional Review Board or ethics committee approval.

### Method details

#### Association of MMP-14 mRNA expression with clinicopathological features in bladder cancer

We used RNAseq data of 414 bladder cancer patients in level 3 HTSeq-Fragments Per Kilobase per Million format from TCGA, converted to transcripts per million reads and log2-transformed. We applied a chi-square test to explore associations between the clinicopathological characteristics of patients and MMP-14 expression, divided into low and high according to median. Survminer [version 0.4.9] and ggplot2 [version 3.3.3] were used for visualization.

#### Screening of MMP-14-related genes and enrichment analysis

Based on the differential expression level of MMP-14 in bladder cancer from TCGA data, DESeq2 was applied for screening DEGs using the Wilcoxon rank-sum test and the Benjamini–Hochberg procedure.^48^^,^^49^ The filtering criteria were an abs|log2(fold change)|>1 and an adjusted *p* value < 0.05. The corresponding CGs were screened by Pearson correlation analysis, |correlation coefficient|>0.3 and adjusted *p* value < 0.05. The clusterProfiler package [version 3.14.3] was used to perform functional enrichment analyses of TCGA data. FDR<0.25 and adjusted *p* value < 0.05 indicated statistical significance.

#### Analysis of MMP-14 transcription factors

The common transcription factors of MMP-14 between the Knock TF database and hTFtarget database were screened using a Venn diagram.^50^^,^^51^ TCGA data were analyzed for correlation between MMP-14 and the transcription factors, ggplot2 [version 3.3.3] was used for visualization.

#### Immune infiltration analysis

We used single-sample GSEA and the Gene Set Variation Analysis (GSVA) package [version 1.34.0] to determine the correlation of MMP-14 expression with the abundance of CD8^+^ T cells and Treg cells in bladder cancer.^52^^,^^53^

#### Correlation analysis of MMP-14 with immune cell markers

After correcting for tumor purity, the TIMER database was used for correlation analysis of MMP-14 with markers of CD8^+^ T-cells, Treg cells, and T cell exhaustion in bladder cancer tissues. The GEPIA database was used to analyze the associations of MMP-14 with these markers. Pearson correlation analysis was used.

#### Inhibitors and activators

The STAT3-specific inhibitor HO-3867 (Selleckchem, USA, Cat#S7501), and the MMP-14-specific inhibitor NSC405020 (Selleckchem, USA, Cat#S8072), which specifically targets the hemopexin (PEX) domain, were dissolved in DMSO, which was diluted >1:10,000 in final concentrations. The STAT3-specific activator colivelin (TOCRIS, USA, Cat#3945) was dissolved in 20% ethanol and used at 0.5 μM.

#### Western blot

The cells were lysed in 50 mM Tris pH 6.8, 1% SDS, 1 mM EDTA, protease inhibitor cocktail (Cell Signaling Technology, USA, Cat#5871) and phosphatase inhibitor (PhosSTOP, Roche, USA, Cat#04906837001) and sonicated. Fifteen μg of protein were separated using a 10% SDS‒polyacrylamide gel. PVDF membranes were incubated with primary antibodies overnight at 4°C. After incubation with secondary antibodies (anti-rabbit IgG, HRP-linked, Cat#7074 and anti-mouse IgG, HRP-linked, Cat#7076, Cell Signaling Technology), proteins were visualized using enhanced chemiluminescence reagent (WBLUF0100, Merck, USA). The optical density was determined with ImageJ, expression was normalized to beta-actin or GAPDH. Antibody details are provided in the key resources table.

#### Immunofluorescence

Cells cultured for 48 h on 8-well RS glass slides (Thermo Scientific, USA, Cat#154534) were fixed with 3% paraformaldehyde and incubated with primary antibodies at 4°C overnight. After incubation with a secondary antibody (goat anti-rabbit IgG (H + L), Alexa Fluor 594, Invitrogen, USA, Cat#A-11072), cell nuclei were stained with bisbenzimide (4 μg/mL, Merck, Germany, Cat#B2261). Cells were covered with fluorescence mounting medium (Dako, Germany, Cat#F4680), Antibody details are provided in the key resources table.

#### Detection of the half-maximal inhibitory concentration (IC50) of the inhibitors

One day after seeding in 96-well plates, different concentrations of HO-3867 or NSC405020 were applied to the cells for 48 h. Cell viability (five replicates each) was determined using the Colorimetric Cell Viability Kit I (WST-8, PromoKine, France, Cat#PK-CA705-CK04), the IC50 value was calculated in a dose‒response fitting curve.

#### Cell proliferation assay

WST-8 cell proliferation assays were performed according to the manufacturer’s instructions. Briefly, cell viability (five replicates each) was determined at IC50 concentration up to three days. Ten μL of WST-8 solution was added to each well, and after incubation for 2 h, the absorbance was measured.

#### Cell cytotoxicity assay

Cell cytotoxicity was measured (three replicates each) using the Cytotoxicity Detection Kit Plus (Roche, Germany, Cat#04744926001) according to the manufacturer’s protocol. In the experimental group, the IC50 inhibitor concentration was added, high control got cell lysis buffer and low control normal cell culture medium. Detection was carried out up to 48 h.

#### MMP-14 activity assay

MMP-14 activity was detected with an MMP-14 activity kit (QuickZyme, Netherlands, Cat#QZBMMP-14H) according to the user guide. Sample extracts of cells cultured with IC50 of inhibitor (three replicates each) were collected at 24 h and 48 h. After coating a microplate with MMP-14 antibody and blocking, extracts were incubated for 24 h, absorbance was measured. MMP-14 activity was calculated according to the standard curve and normalized to that of the control.

#### Establishment of cells stably overexpressing MMP-14

Transfection of RT-112 cells in 6-well plates was performed according to the instructions of Biocat (Germany) using the transfection promoter EndoFectin Max transfection reagent (EF013-GC), the MMP-14 overexpression plasmid (pEZ-M61, EX-A6431-M61-GC) and the corresponding empty control vector (pReceiver-M61 CT, EX-NEG-M61-GC). After optimization, 2.5 μg of plasmid and 7.5 μL of EndoFectin Max transfection reagent were preincubated and added to each well at 40-50% cell density. Twenty-four hours later, transfected cells were selected using 500 μg/mL G418 Sulfate (Thermo Fisher, USA, Cat#10131027). After 14 days, four surviving clones expressing cytofluorescence were expanded and maintained in culture with 400 μg/mL geneticin.

#### Generation of stable MMP-14-knockdown cells

Similar to MMP-14 overexpression, transfection of HT-1376 cells was performed according to the instructions of Biocat using three sgRNA/Cas9 clones targeting MMP-14 (pCRISPR-CG04, HCP302442-CG04-3-10-GC) and a scramble sgRNA control (pCRISPR-CG04, CCPCTR01-CG04-10-GC). After transfection with 2.5 μg of plasmid and 10 μL of EndoFectin Max transfection reagent, cells were selected using 500 μg/mL G418 Sulfate. Twenty-five days later, six surviving clones were expanded and maintained in culture with 500 μg/mL geneticin. Only 10b clones out of the 3 MMP-14 target plasmids were able to grow.

#### Cell death detection assay

The Cell Death Detection ELISA PLUS (Roche, Germany, Cat#11774425001) was used to measure apoptosis. Briefly, three replicates each in 96-well plates were collected with lysis buffer after 24 and 48 h with IC50 inhibitor or after 48 and 96 h for transfected cells. Following incubation with immunoreagent for 2 h and staining by ABTS solution, the absorbance was measured at 405 nm. The apoptosis index was calculated: apoptosis index = absorbance of sample/absorbance of control.

#### PBMC isolation and T cell enrichment

Lymphoprep (Stemcell Technology, Germany, Cat#07801) was used to isolate fresh human peripheral blood mononuclear cells (PBMCs). Fifteen milliliters of Lymphoprep were added to the lower layer of SepMate-50 (IVD) (Stemcell Technology, Cat#85450), and the upper layer was filled with 1:1 diluted human blood. After centrifugation, PBMCs were isolated, washed and counted. Magnetic cell separation technique (MACS) was used to enrich CD3^+^ T cells. PBMCs resuspended in MACS buffer and CD3 MicroBeads (Miltenyi Biotec, USA, Cat#130-050-101) were incubated for 15 min at 4°C, washed, and CD3-positive T cells were separated via a 20 μm preseparation filter and an MS column with a magnetic stand.

#### T cell activation and culture

Twenty-four-well plates were coated with CD3 (1 μg/mL, Invitrogen, USA, Cat#16-0037-85) and CD28 (1 μg/mL, BioLegend, USA, Cat#302934), in which 1 × 10^6^ T cells per well were cultured with CTL medium (44.5% CLICK’s medium, 44.5% Advanced RPMI medium, 10% FBS, 1% L-glutamine) supplemented with IL-2 (100 IU/mL, Miltenyi Biotec, USA, Cat#130-097-748), IL-7 (10 ng/mL, CellGenix, Germany, Cat#1410-050), and IL-15 (5 ng/mL, CellGenix, Cat#1413-50). Medium was changed every two days.

#### Coculture and flow cytometry

Stimulated CD3^+^ T cells were cocultured with HT-1376, RT-112, a corresponding KD or OE clone for 24 h (T cells: cancer cells = 10:1) for flow cytometry. CD3 staining and FSC-A (or SSC-W) were used to distinguish between cancer cells and T cells. An Annexin V-FITC apoptosis detection kit (Sigma‒Aldrich, Germany, Cat#APOAF) was used for cancer cell apoptosis according to the manufacturer’s instructions. Surface staining (for PD-1, TIM-3, and LAG-3) and intracellular staining (for perforin and granzyme B) of T cells was performed using the BD Cytofix/Cytoperm Fixation/Permeabilization Kit (BD Biosciences, USA, Cat#554714) according to the manufacturer’s instructions. Flow cytometry was carried out using the BD LSRFortessa Cell Analyzer and DIVA8 (BD Biosciences), data were analyzed by FlowJo (version 10.4). The hierarchical gating strategy utilized FSC-A/SSC-A for initial morphological isolation, followed by doublet exclusion (FSC-H/FSC-W) and the use of a fixable viability dye (Live/Dead) to restrict analysis strictly to live CD3^+^ singlets and prevent non-specific antibody binding artifacts. The proportion of positive cells was quantified, with thresholds for PD-1+, TIM-3+, and LAG-3+ populations strictly established using Fluorescence Minus One (FMO) and isotype controls.

#### Antibody array

The expression levels of 37 human phosphokinases in HT-1376 cells were measured with a Human Phospho-Kinase Array Kit (R&D Systems, ARY003C, USA) according to the instructions. Briefly, HT-1376 control cells or cells treated with NSC405020 at IC50 for 24 h were lysed, 500 μg protein each was added to the membranes and incubated at 4°C overnight, followed by incubation with detection antibody cocktail/streptavidin-HRP and chemiluminescence imaging. Detection by ImageJ used the reference point to normalize the loading amount, and the fold change for gray values was: fold change = (NSC405020/NSC405020 reference point)/(control/control reference point).

#### Coimmunoprecipitation

A Pierce Crosslink Magnetic IP/Co-IP Kit (Thermo Scientific, USA, Cat#88805) was used to perform protein coimmunoprecipitation from HT-1376 and RT-112 cells according to the manufacturer’s instructions. Briefly, 5 μL beads and 10 μg of MMP-14 antibody, STAT3 antibody, rabbit IgG XP or mouse IgG2a isotype control antibody (key resources table) were coupled and crosslinked. One milligram of protein lysate was incubated with antibody-crosslinked beads for 4 h at 4°C. After elution, Western blotting was used to detect the precipitated proteins.

#### Silver staining

Silver staining was carried out after coimmunoprecipitation and protein gel electrophoresis with a Pierce Silver Stain Kit (Thermo Scientific, Cat#24612) according to the manufacturer's instructions. Briefly, the gel was fixed, sensitized, and stained in Stain Working Solution. After development until bands appeared, five percent acetic acid stopped the process. *n* = 1.

#### LC-MS/MS instrument settings and data analysis

Coimmunoprecipitation for mass spectrometry (*n* = 3 MMP-14 versus control for total co-IP proteins and *n* = 1 for silver stain to excise bands) was performed using the Pierce MS-Compatible Magnetic IP Kit (90409, Thermo Scientific) following the manufacturer’s instructions. In brief, the cells were lysed, and 1 mg of protein was incubated with 10 μg of MMP-14 antibody or rabbit IgG XP isotype control antibody overnight at 4°C. Antibody details are given in the key resources table. The antigen/antibody complex was bound to Protein A/G magnetic beads for 1 h at room temperature before being subjected to tryptic digestion on the beads or electrophoresis/silver staining. Prominent bands from one sample were cut from a silver-stained gel with the following approximate molecular weights: (1) 45 kDa, (2) 50-55 kDa, (3) 55 kDa, (4) 60-65 kDa, (5) 70 kDa, and (6) 90 kDa and were tryptically in-gel digested,^54^ followed by mass spectrometry. All coimmunoprecipitates were subjected to complete mass spectrometric analysis. LC‒MS/MS was carried out by nanoflow reverse-phase liquid chromatography (Dionex Ultimate 3000, Thermo Scientific) coupled online to a Q Exactive HF Orbitrap mass spectrometer (Thermo Scientific), as reported previously.^55^ LC separation was performed using a PicoFrit analytical column (75 μm ID × 50 cm long, 15 μm Tip ID; New Objectives, Woburn, MA) packed in house with a 3 μm C18 resin (Reprosil-AQ Pur, Dr. Maisch, Ammerbuch, Germany). Peptides were eluted using a gradient from 3.8 to 38% solvent B in solvent A over 120 min at 266 nL per minute flow rate. Solvent A was 0.1% formic acid, and solvent B was 79.9% acetonitrile, 20% H_2_O, and 0.1% formic acid. Nanoelectrospray was generated by applying 3.5 kV. A cycle of one full Fourier transformation scan mass spectrum (300−1750 m/z, resolution of 60,000 at m/z 200, automatic gain control (AGC) target 1 × 10^6^) was followed by 12 data-dependent MS/MS scans (resolution of 30,000, AGC target 5 × 10^5^) with a normalized collision energy of 25 eV. To avoid repeated sequencing of the same peptides, a dynamic exclusion window of 30 s was used. The raw MS data were processed with MaxQuant software (v2.2.0.0) and searched against the human proteome database UniProtKB: UP000005640 with 81,791 protein entries released in March 2023. The parameters of the MaxQuant database were a false discovery rate (FDR) of 0.01 for proteins and peptides, a minimum peptide length of seven amino acids, a first search mass tolerance for peptides of 20 ppm, and a main search tolerance of 4.5 ppm. A maximum of two missed cleavages were allowed for the tryptic digest. Cysteine carbamidomethylation was set as a fixed modification, while N-terminal acetylation and methionine oxidation were set as variable modifications.

### Quantification and statistical analysis

Experiments were performed three times with UM-UC-3, RT-4, RT-112 and HT-1376 cells unless otherwise stated; n represents the number of independent experiments, and results are given as mean ± SD. The data were statistically analyzed using SPSS 26.0 or GraphPad Prism 9.0 software and tested for normality by the Shapiro-Wilk test. The Mann-Whitney U test or Wilcoxon signed-rank test was used for nonparametric analyses, and parametric tests were performed using the Student test. Comparison among multiple groups was performed using one-way ANOVA or two-way ANOVA, and Bonferroni or Dunnett post hoc tests for correction. Association analysis of categorical variables was carried out by Chi-square test or Fisher’s exact test. Correlation of continuous variables was analyzed by Pearson or Spearman correlation analysis. Two-sided *p* < 0.05 was considered to indicate statistical significance. In the figures, statistical significance is indicated as follows: ns, not significant; ∗*p* < 0.05; ∗∗*p* < 0.01; ∗∗∗*p* < 0.001.

SPSS software (version 26.0) and R software (version 3.6.3) were used for statistical analysis. Western blot band intensities were quantified with ImageJ, and flow cytometry data were analyzed with FlowJo (version 10.4). Statistical details of the bioinformatic analyses, including the screening thresholds for DEGs and correlated genes, the ssGSEA algorithm, and the TIMER/GEPIA correlation analyses, are provided in the methods and in the figure legends.

### Additional resources

The Cancer Genome Atlas (TCGA) Research Network data used for all bioinformatic analyses: https://portal.gdc.cancer.gov/

TIMER (immune cell marker correlation analysis): https://cistrome.shinyapps.io/timer/

GEPIA (gene expression correlation analysis): https://gepia.cancer-pku.cn/

KnockTF (transcription factor target screening): https://www.licpathway.net/KnockTF/

hTFtarget (transcription factor target screening): https://bioinfo.life.hust.edu.cn/hTFtarget/.

## Supplemental information

Document S1. Figures S1–S6 and Tables S1

Table S2. List of genes from TCGA data to select MMP-14-associated differentially expressed genes, related to Figure 1

Table S3. List of genes from TCGA data to select MMP-14-correlated genes, related to Figure 1

Table S4. List of genes from TCGA data to select common MMP-14-associated differentially expressed genes and MMP-14-correlated genes, related to Figure 1

Table S5. List of human phosphorylated kinase antibody arrays, related to Figure 6

Table S6. Mass spectrometry data of 3 MMP-14 coimmunoprecipitates, related to Figure 6

Table S7. Mass spectrometry data of 6 gel fragments from MMP-14 coimmunoprecipitation followed by silver staining, related to Figure 6
