# Supplementary material for: An MMP-14/STAT3 feedback loop upregulates PD-L1 to drive immunosuppression and tumorigenesis in bladder cancer
Source: iScience. 2026 Oct 28;29(11):118071. doi: 10.1016/j.isci.2026.118071 (PMC13738172; doi:10.1016/j.isci.2026.118071)
Supplement: Supplementary file 1 [file mmc1.pdf]

iScience, Volume *29*

Supplemental information

An MMP-14/STAT3 feedback loop upregulates PD-L1 to drive immunosuppression and tumorigenesis in bladder cancer

Shuai Zhu, Lisa Köhler, Bettina Ergün, David Meierhofer, Jonas Busch, and Anja Rabien

Supplementary Material

Fig. S1 Proliferation and cytotoxicity of MMP-14-inhibited bladder cancer cell lines.

Fig. S2 Proliferation and cytotoxicity of STAT3-inhibited bladder cancer cell lines.

Fig. S3 KEGG pathway enrichment analysis of the MMP-14 TCGA data to explore the possible signaling pathways related to common DEGs and MMP-14-related genes in bladder cancer.

Fig. S4 Selected MMP-14-associated proteins from coimmunoprecipitation.

Fig. S5 Western blot verification of MMP-14-associated proteins in HT-1376 cells identified by mass spectrometry.

Fig. S6 Original Western blot

Table S1 Correlations between MMP-14 expression and clinicopathological characteristics of patients with bladder cancer (TCGA data)

Excel tables in separate files:

Table S2 List of genes from TCGA data to select MMP-14-associated differentially expressed genes.

Table S3 List of genes from TCGA data to select MMP-14-correlated genes.

Table S4 List of genes from TCGA data to select common MMP-14-associated differentially expressed genes and MMP-14-correlated genes.

Table S5 List of human phosphorylated kinase antibody arrays.

Table S6 Mass spectrometry data of three MMP-14 coimmunoprecipitates.

Table S7 Mass spectrometry data of six gel fragments from MMP-14 coimmunoprecipitation followed by silver staining.

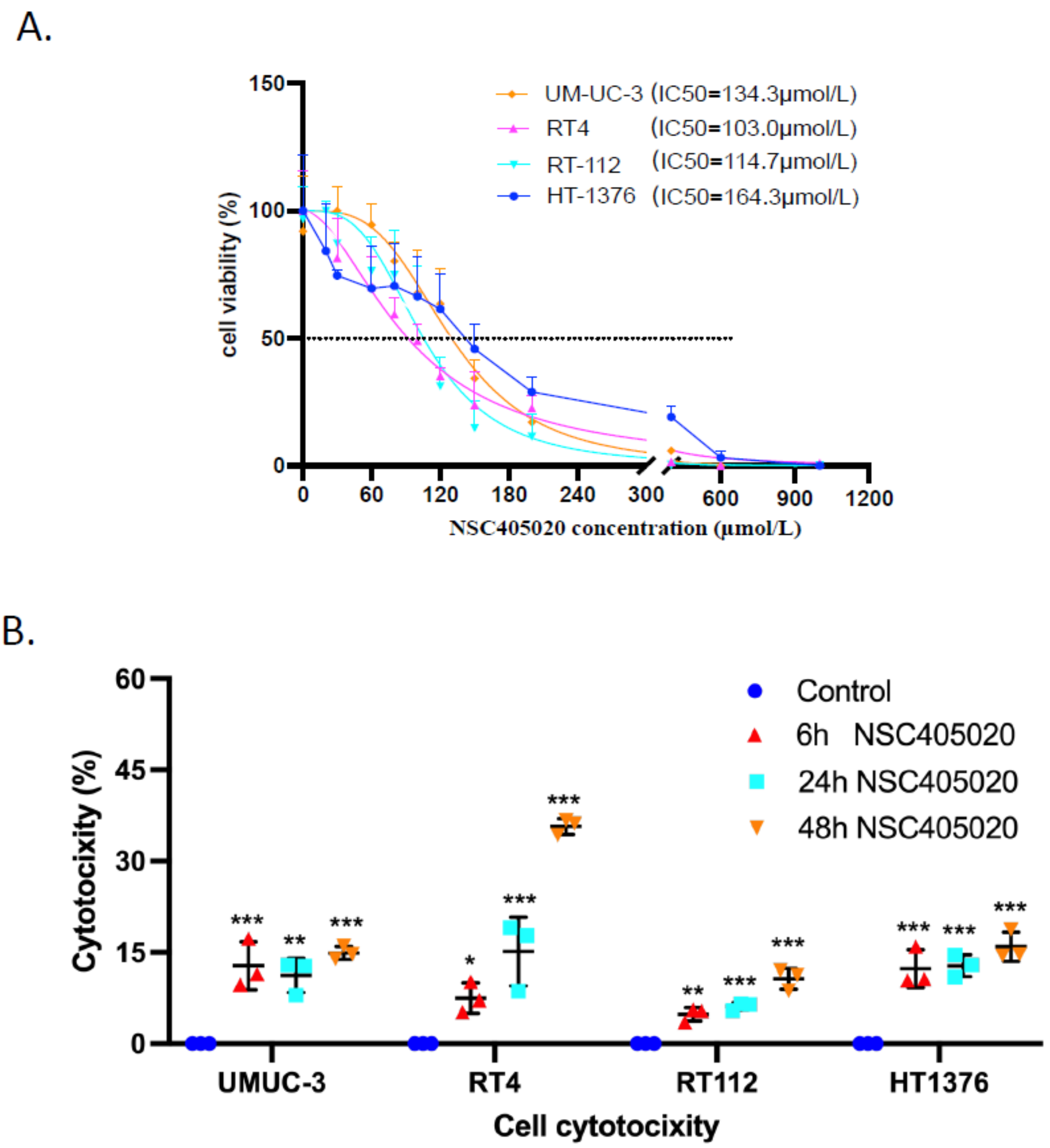

Fig. S1 Proliferation and cytotoxicity of MMP-14-inhibited bladder cancer cell lines.
A: A WST-8 assay was used to determine the 50% inhibitory concentration (IC 50) of NSC405020 in four bladder cancer cell lines. B: LDH assays were used to detect drug-induced cytotoxicity in bladder cancer cells after treatment with NSC405020 for 6 h, 24 h or 48 h. One-way ANOVA was used to identify significant results, and Dunnett's procedure was used for multiple comparisons; (n=3). Data are represented as mean ± SD. ns, not significant; *$p < 0.05$, **$p < 0.01$, ***$p < 0.001$.

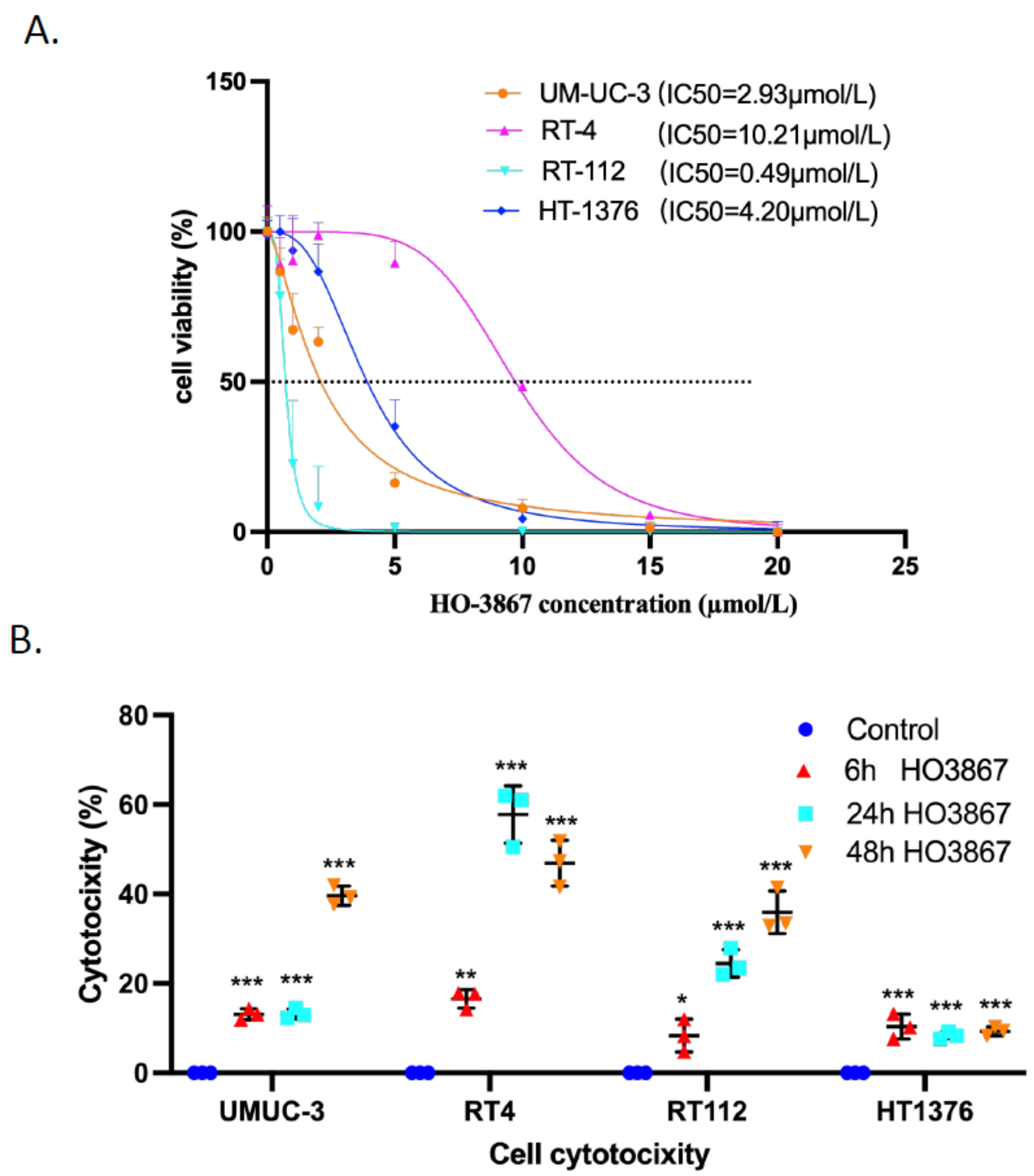

Fig. S2 Proliferation and cytotoxicity of STAT3-inhibited bladder cancer cell lines.
A: A WST-8 assay was used to determine the 50% inhibitory concentration (IC 50) of HO-3867 in four bladder cancer cell lines. B: LDH assays were used to detect drug-induced cytotoxicity in bladder cancer cells after treatment with HO-3867 for 6 h, 24 h or 48 h. One-way ANOVA was used to identify significant results, and Dunnett's procedure was used for multiple comparisons; (n=3). Data are represented as mean ± SD. ns, not significant; *$p < 0.05$, **$p < 0.01$, ***$p < 0.001$.

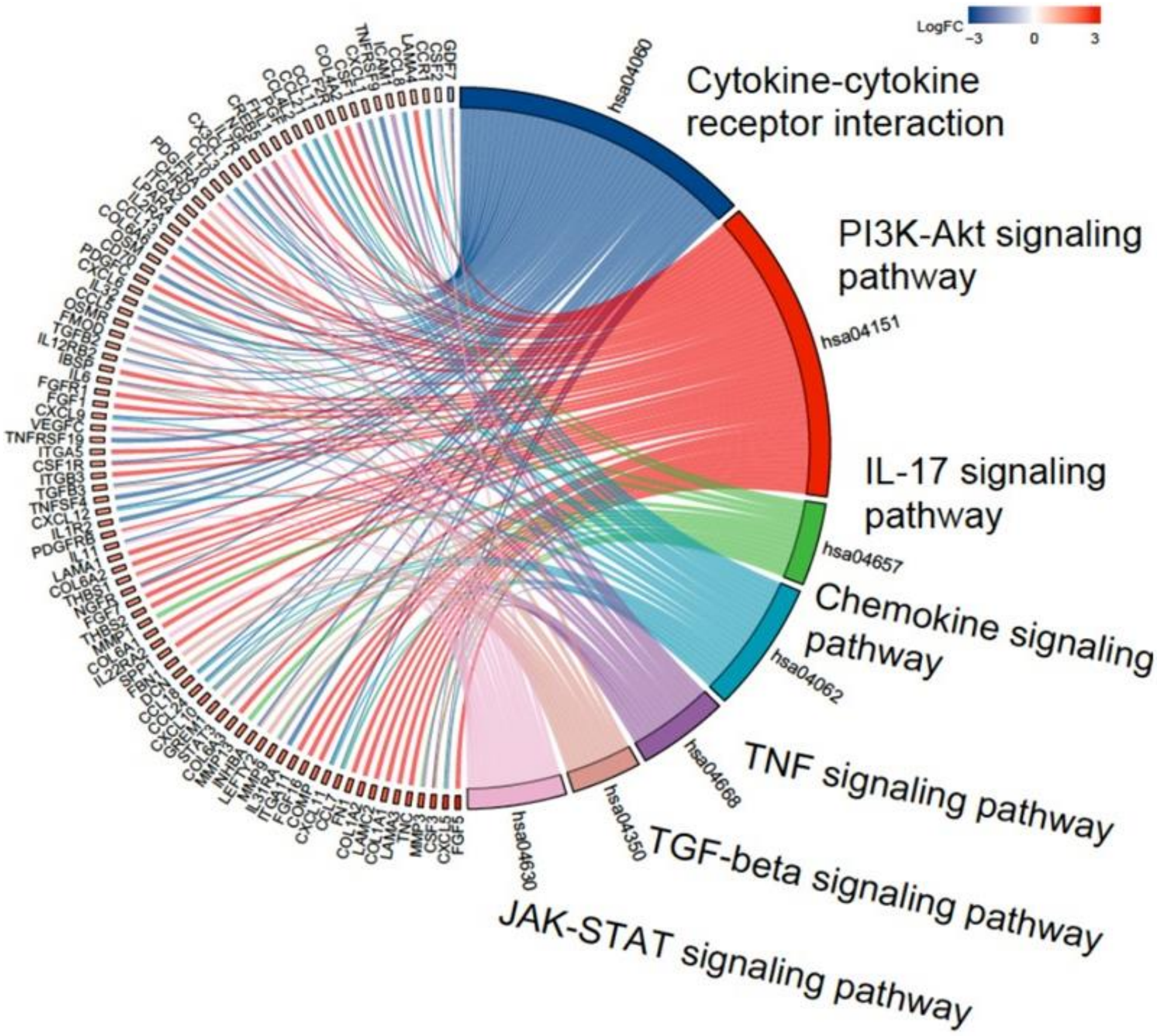

Fig. S3 KEGG pathway enrichment analysis of the MMP-14 TCGA data to explore the possible signaling pathways related to common DEGs and MMP-14-related genes in bladder cancer.

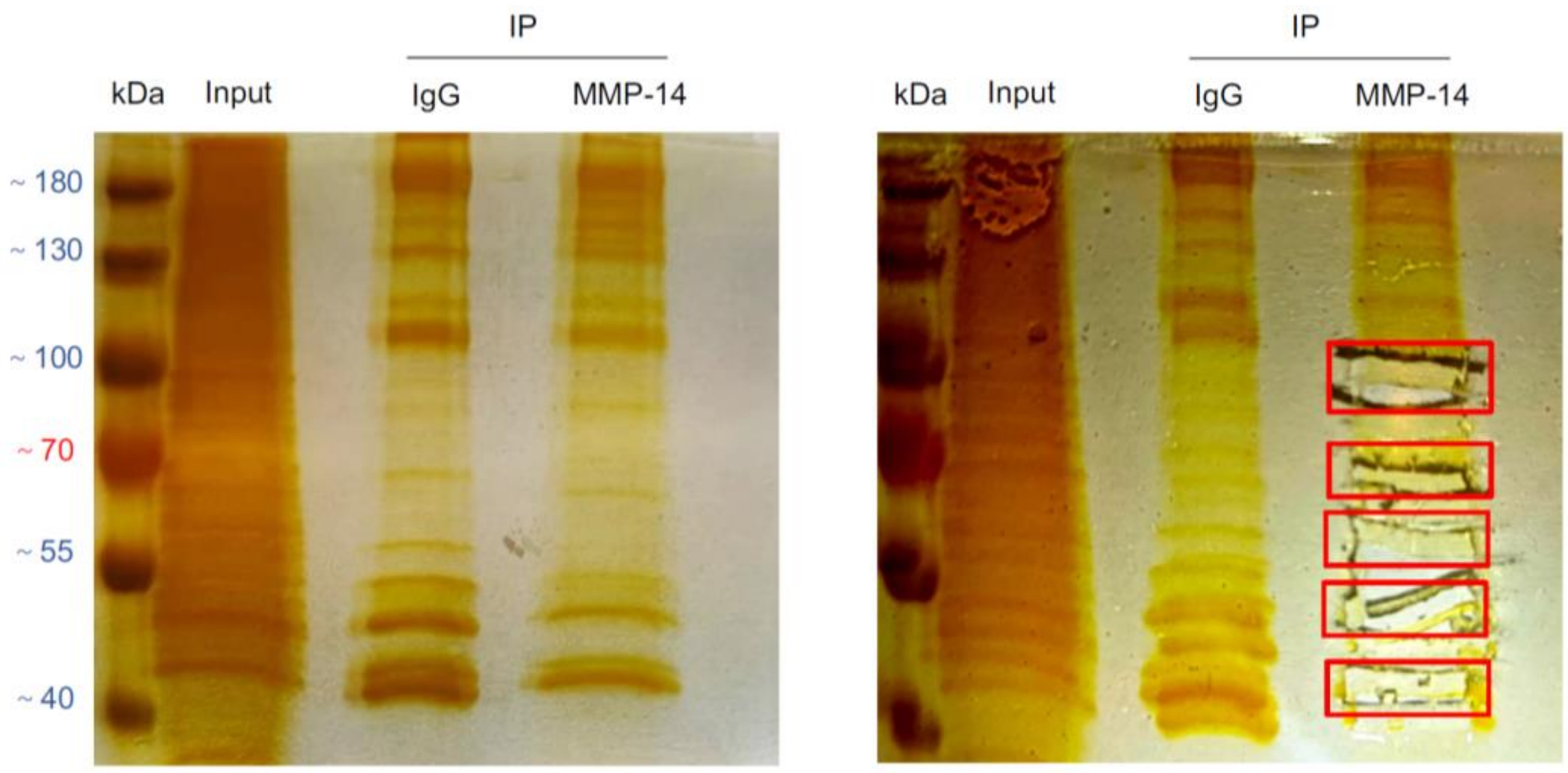

Fig. S4 Selected MMP-14-associated proteins from coimmunoprecipitation.
Silver staining after MMP-14 coimmunoprecipitation was used to show proteins to which MMP-14 may bind in HT-1376 cells. Gel bands of approximately 45 kDa, 50-55 kDa, 55 kDa, 60-65 kDa, 70 kDa and 90 kDa were cut out assuming PD-L1, MMP-14 and STAT3 inclusion. Each gel fragment was processed for mass spectrometry. The 50-55 kDa and 55 kDa gel bands were close together, so their cut-out locations are shown together in one red box.

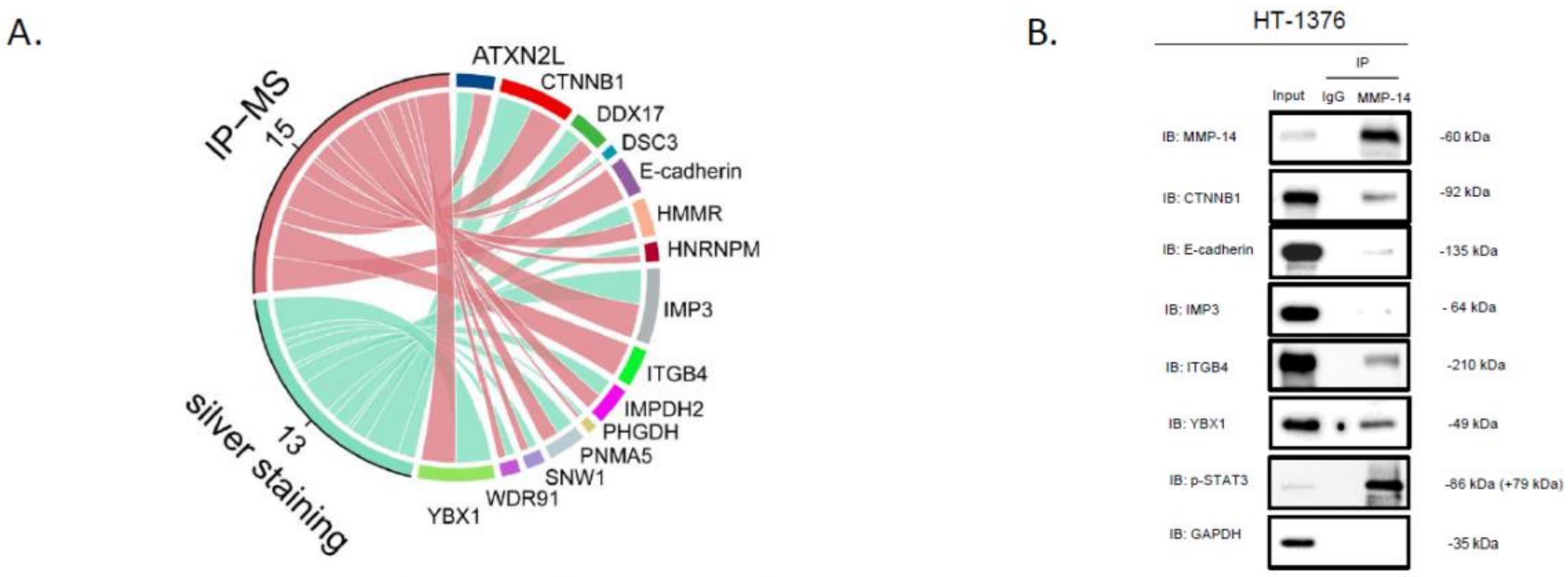

Table of protein information identified by mass spectrometry

Gene	Protein	Molecular weight	Internal relevant information
ATXN2L	ataxin 2-like protein	113 kDa	many splice variants, not much known, neurodegenerative disorders
CDH1	E-cadherin	97 kDa	many splice variants, tumor/invasion suppressor, neg. prognostic marker for bladder cancer
CTNNB1	catenin beta-1	77 kDa	many splice variants, direct interaction with MMP-14 known, but not in bladder cancer
DDX17	DEAD-box helicase 17	80 kDa	many splice variants, tumor promoting, metastasis, EMT
DSC3	desmocollin 3	100 kDa	cell-cell adhesion, cadherin family
HMMR	hyaluronan mediated motility receptor	84 kDa	RHAMM, CD168, cell motility, tumors, metastasis
HNRNPM	heterogeneous nuclear ribonucleoprotein M	78 kDa	associated to bladder clear cell adenocarcinoma (GeneCards)
IGF2BP3	insulin like growth factor 2 mRNA binding protein 3, IMP3	64 kDa	invasion, tumorigenicity, JAK/STAT signaling
ITGB4	integrin beta-4	202 kDa	many splice variants, tumorigenesis, transmembrane protein
IMPDH2	inosine monophosphate dehydrogenase 2	53 - 57 kDa	splice variants, guanine nucleotide synthesis, neoplasms, innate immune system
PHGDH	phosphoglycerate dehydrogenase	56 kDa	many splice variants, L-serine synthesis
PNMA5	paraneoplastic antigen-like protein 5	50 kDa	many splice variants, tumor promoting, metastasis
SNW1	SNW domain-containing protein 1	43 - 65 kDa	many splice variants, RNA helicase, transcriptional processes, tumor
WDR91	WD40 repeat-containing protein 91	42 - 77 kDa	splice variants, neg. PI3K regulator, endosomes
YBX1	Y-box binding protein 1	36 - 42 kDa	splice variants, transcription factor, activates NF-kappaB, tumor promoting

Fig. S5 Western blot verification of MMP-14-associated proteins in HT-1376 cells identified by mass spectrometry.

Selected potentially relevant proteins from mass spectrometry (circular chart and table below) were coimmunoprecipitated using an MMP-14 antibody and analyzed by Western blotting. MMP-14 bound to β-catenin (CTNNB1), E-cadherin (CDH1), insulin-like growth factor 2 mRNA-binding protein 3 (IMP3/IGF2BP3), integrin β4 (ITGB4), Y-box binding protein (YBX1), and also to p-STAT3. GAPDH served as control for the input lysate. Notably, the high-molecular-weight proteins E-cadherin and integrin β4 were not included in the silver-stained bands up to approximately 90 kDa. Some protein molecular weights strikingly differed between mass spec and the blots, probably due e.g. to modifications, behavior in the gel or isoforms. The blots, however, showed the molecular weights given by the antibody companies. Antibody details are given in Table S5.

Original Western blots for Figure 2B, cell lines -/+ NSC405020

Marker: PageRuler Prestained #26616 Thermo Scientific

1	Marker
2	UM-UC-3/47 control
3	UM-UC-3/47 NSC
4	RT-4/41 control
5	RT-4/41 NSC
6	RT-112/45 control
7	RT-112/45 NSC
8	HT-1376/39 control
9	HT-1376/39 NSC

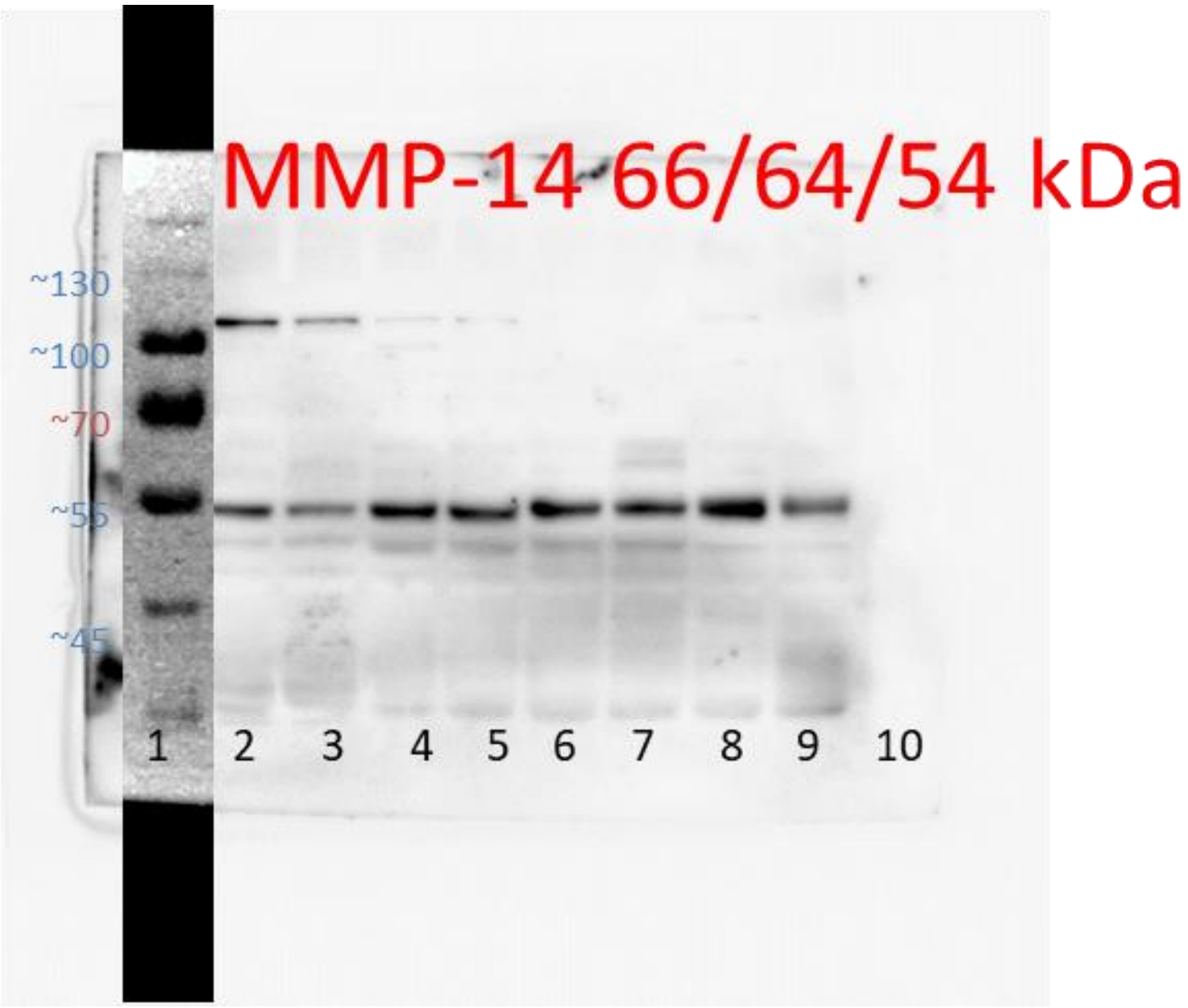

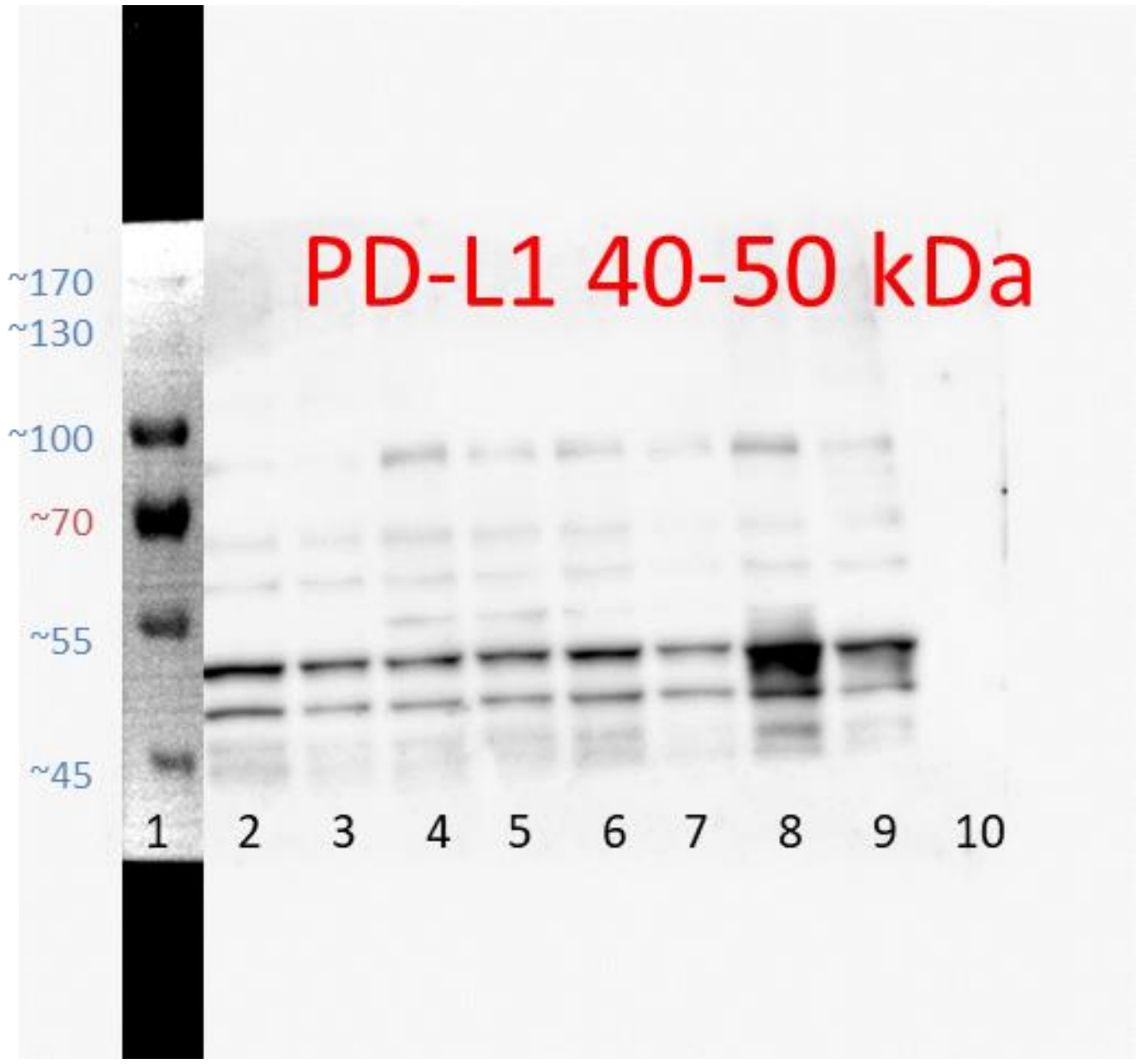

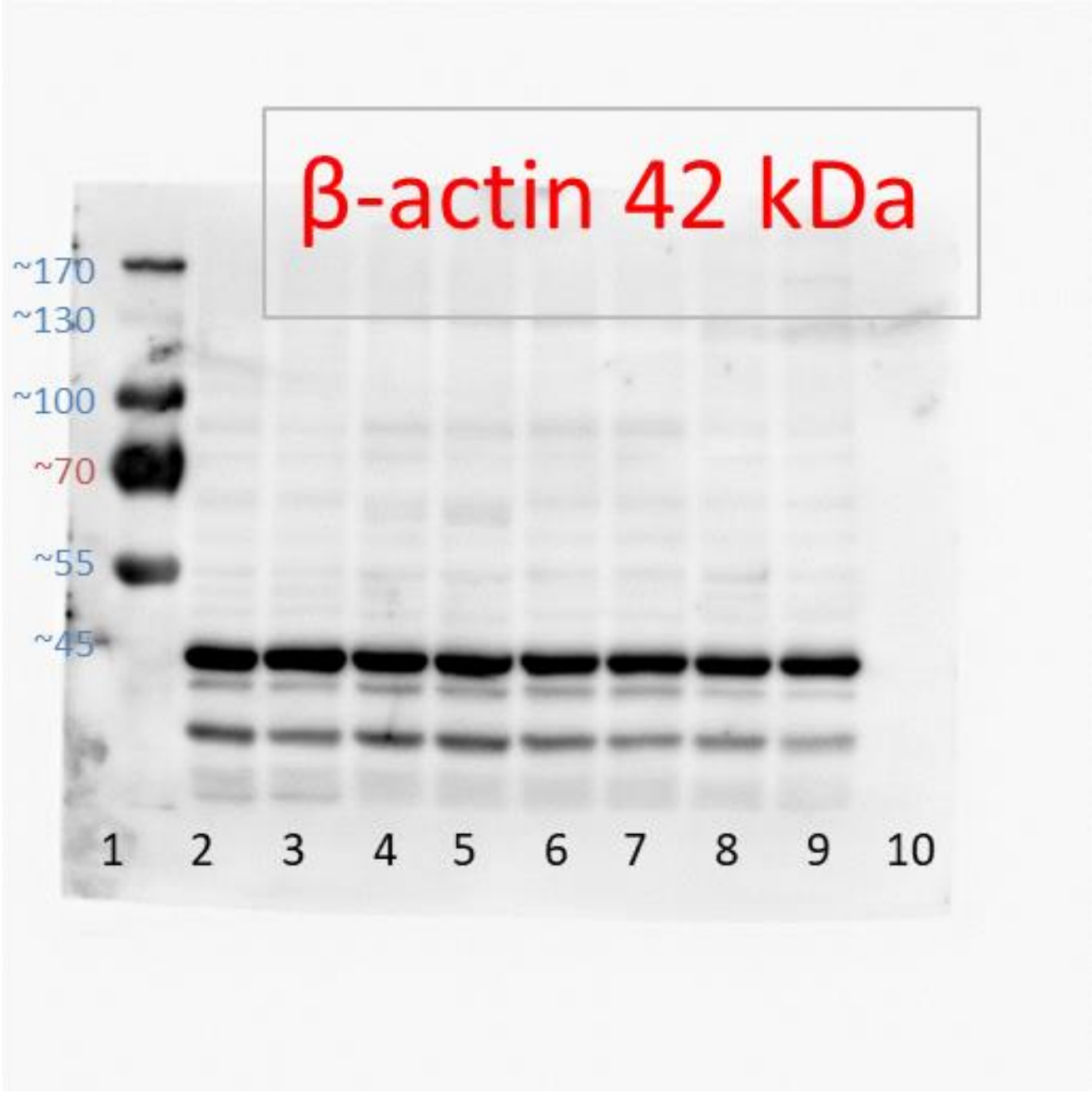

Original Western blots for Figure 3A, HT-1376 MMP-14 KD

Marker: PageRuler Prestained #26616 Thermo Scientific

1	Marker
2	HT control 1.2 3p
3	HT control 2.3 3p
4	HT knockdown 10b 1.1 3p
5	HT knockdown 10b 3.1 3p
6	HT knockdown 10b 3.2 3p
7	HT knockdown 10b 3.3 3p
8	HT knockdown 10b 3.4 3p
9	HT knockdown 10b 4.1 3p

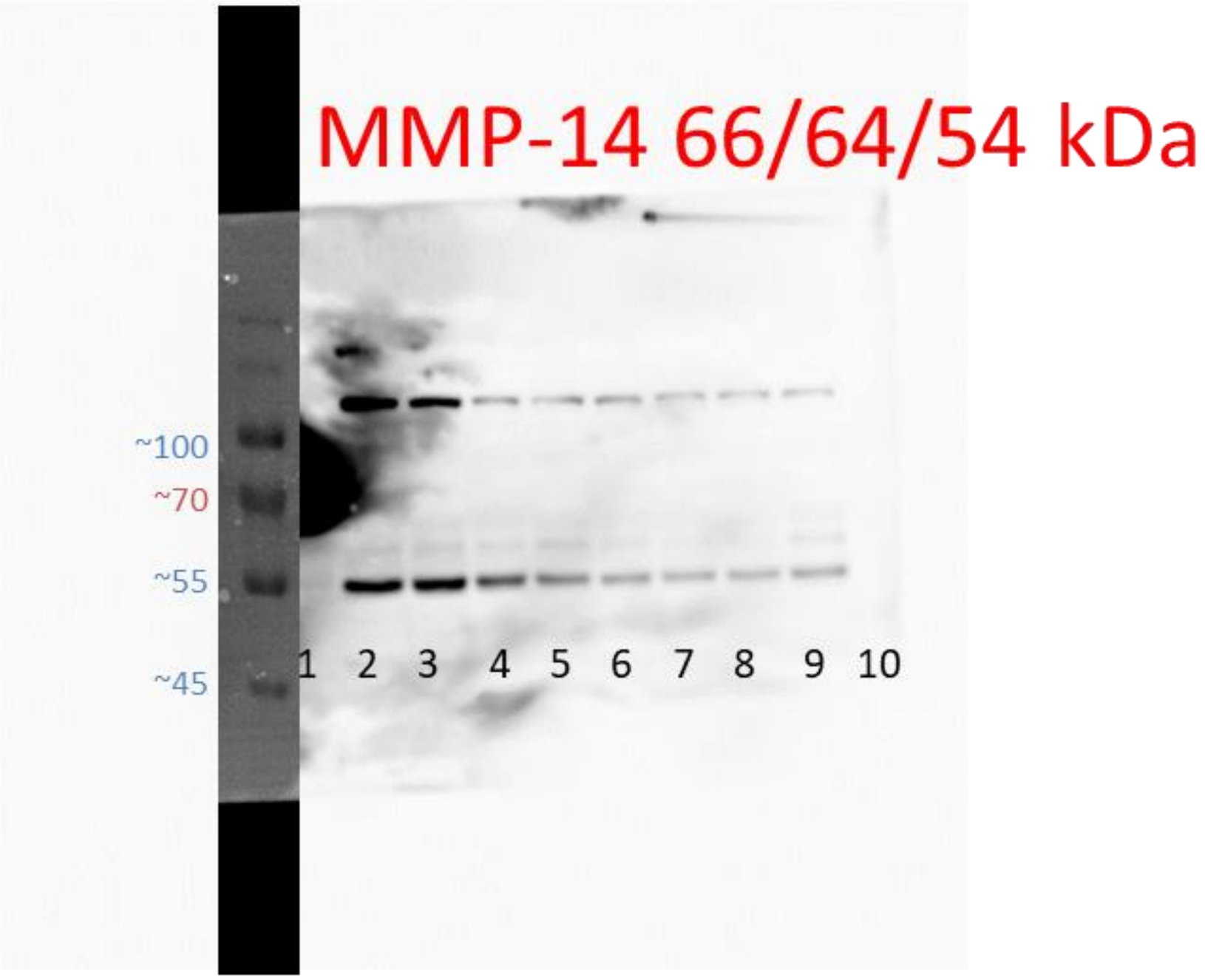

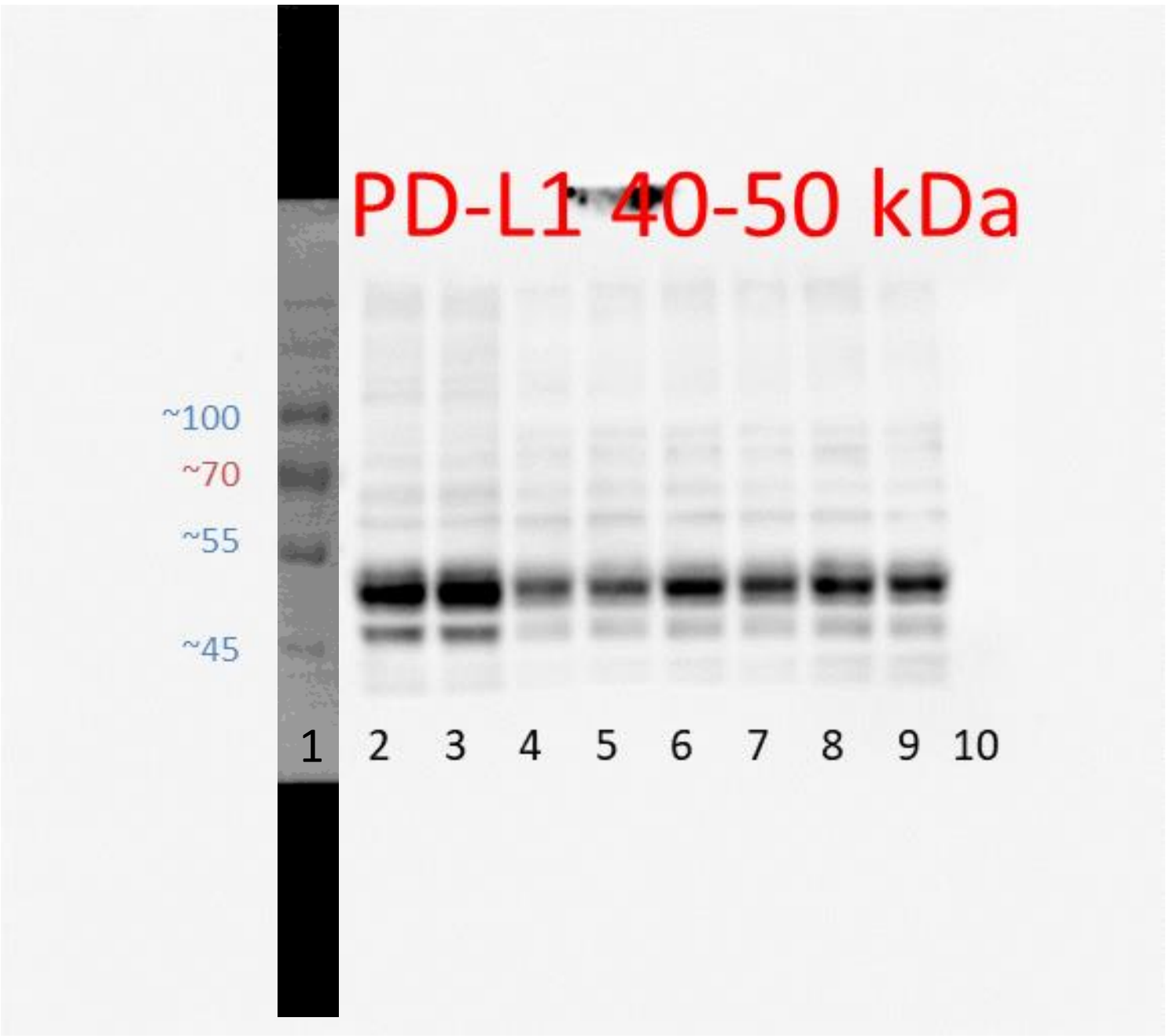

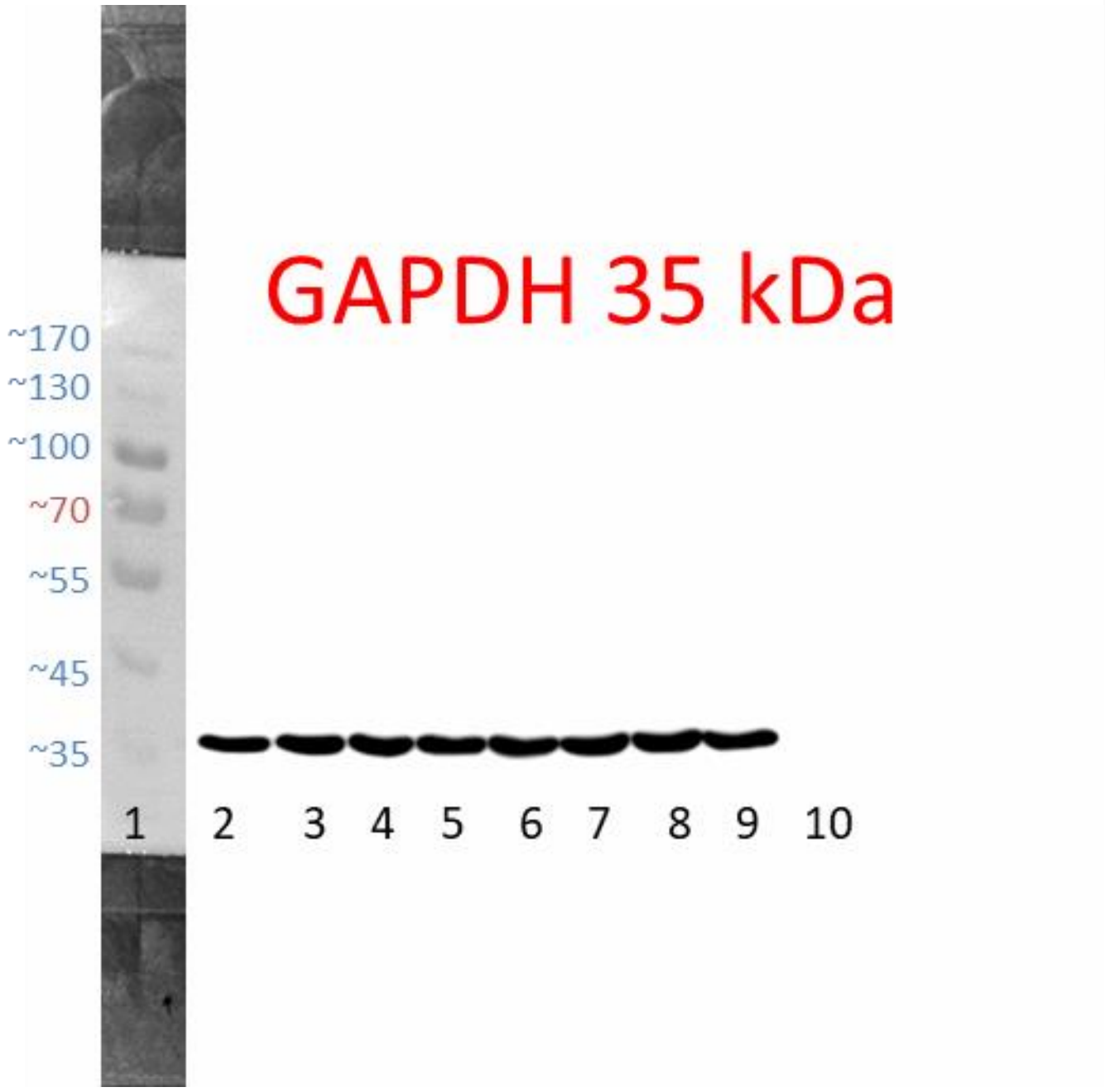

Original Western blots for Figure 3D, RT-112 MMP-14 OE

Marker: PageRuler Prestained #26616 Thermo Scientific

1	Marker	
2	RT-112/vector 1 4p	
3	RT-112/vector 2 4p	
4	RT-112/OE 1 4p	
5	RT-112/OE 2 4p	
6	RT-112/OE 3 4p	
7	RT-112/OE 4 4p	

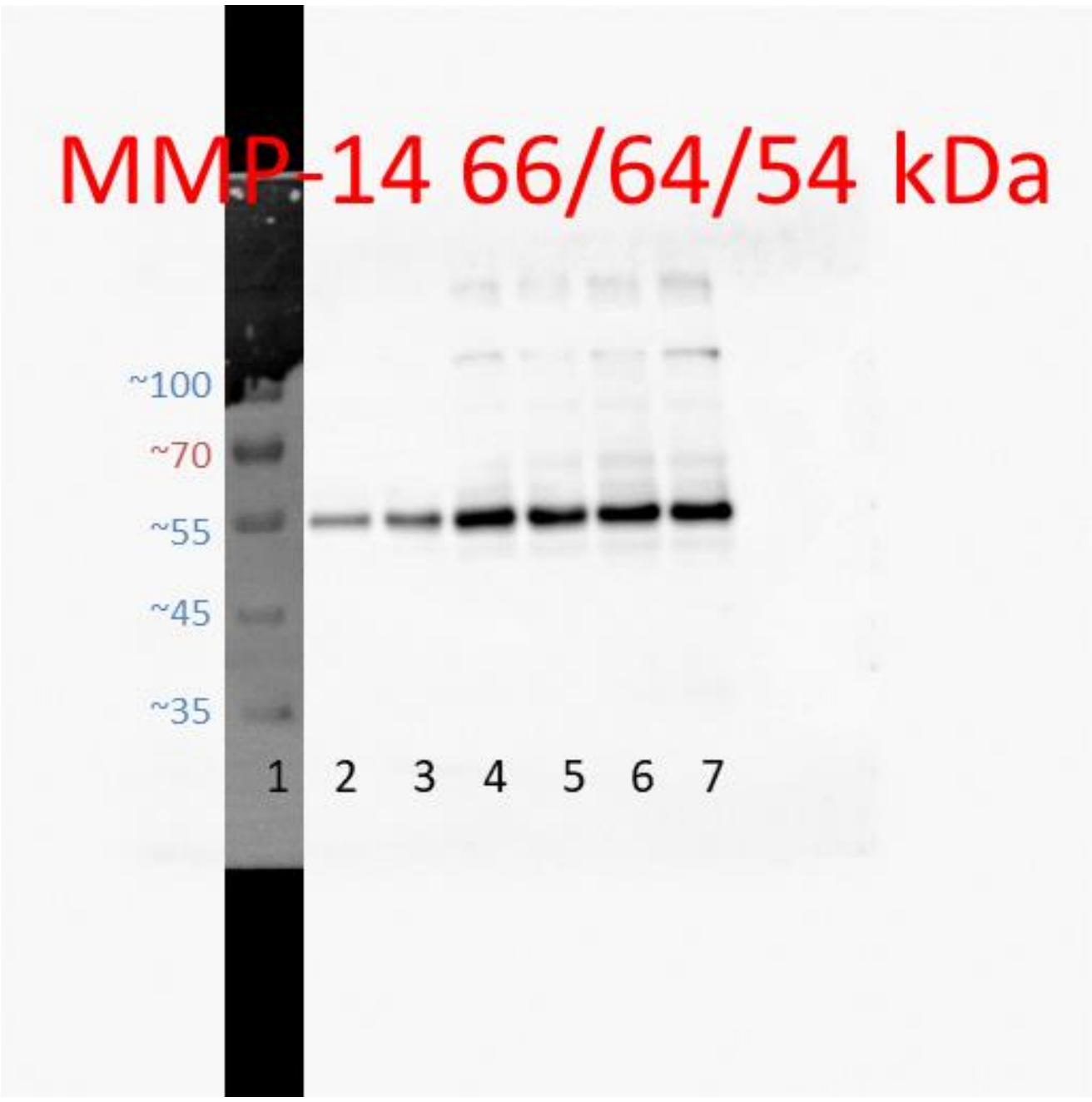

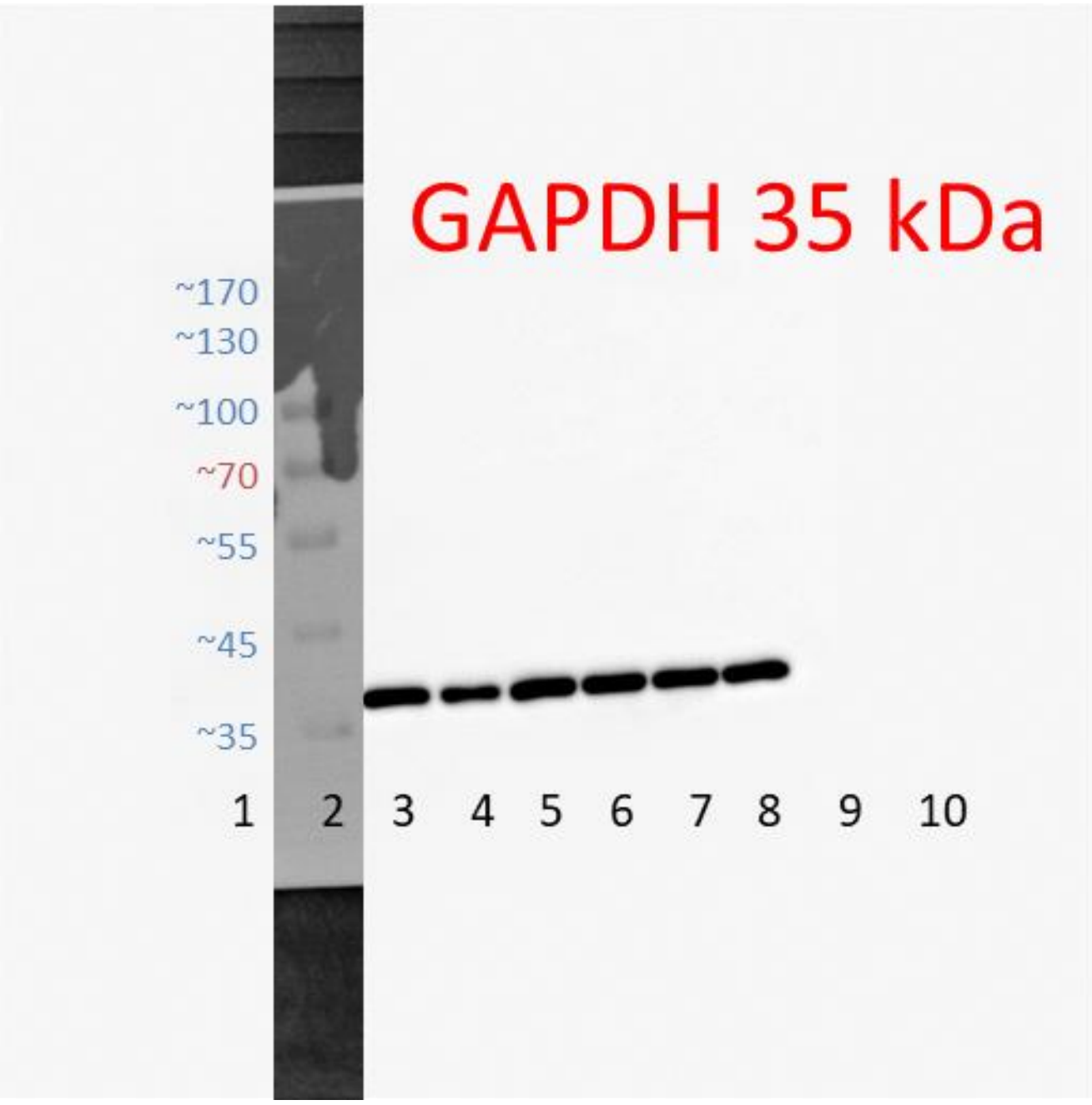

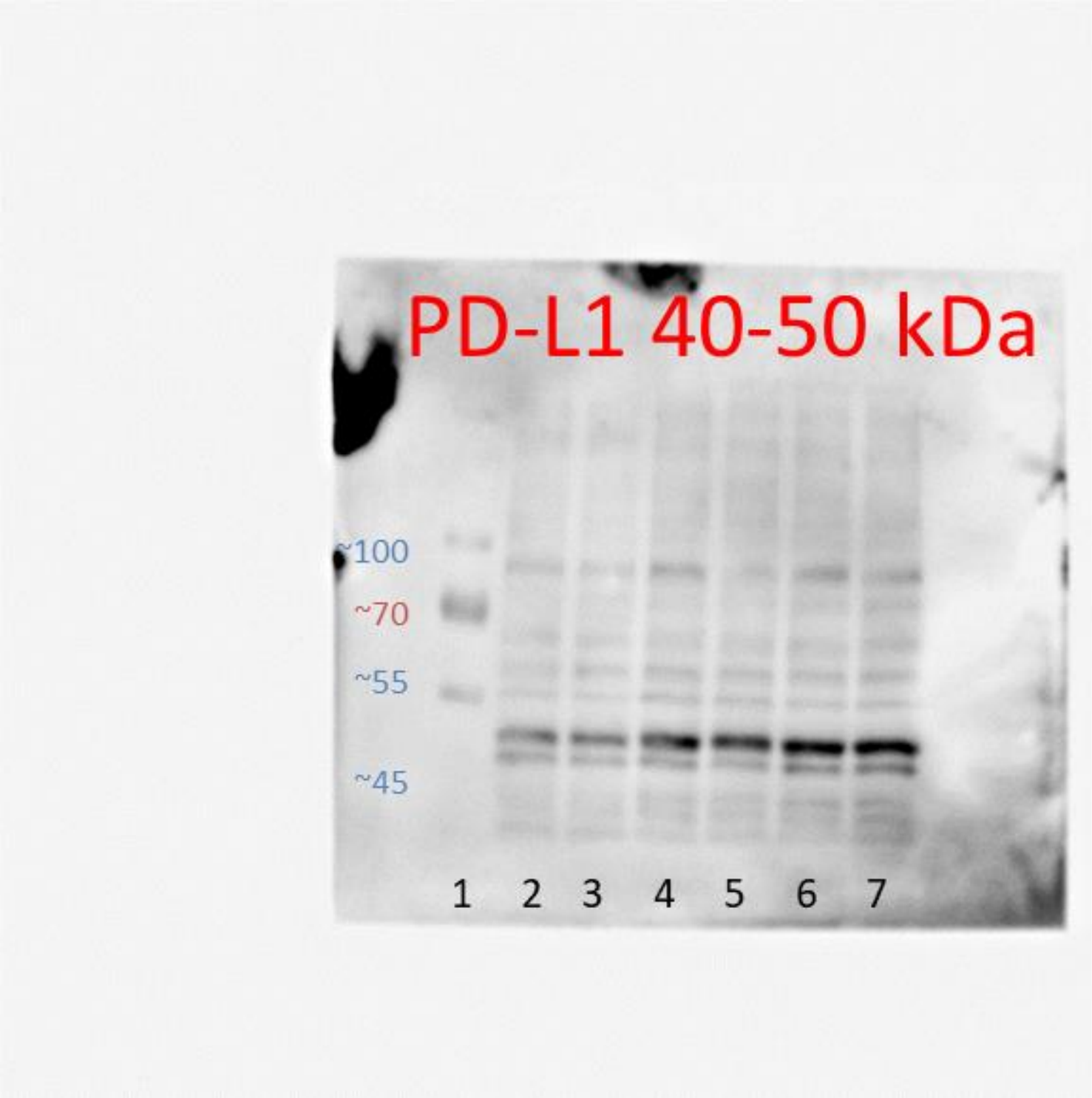

Original Western blots for Figure 5D, cell lines -/+ HO-3867

Marker: PageRuler Prestained #26616 Thermo Scientific

1	Marker
2	UM-UC-3/49 control
3	UM-UC-3/49 HO
4	RT-4/43 control
5	RT-4/43 HO
6	RT-112/47 control
7	RT-112/47 HO
8	HT-1376/41 control
9	HT-1376/41 HO

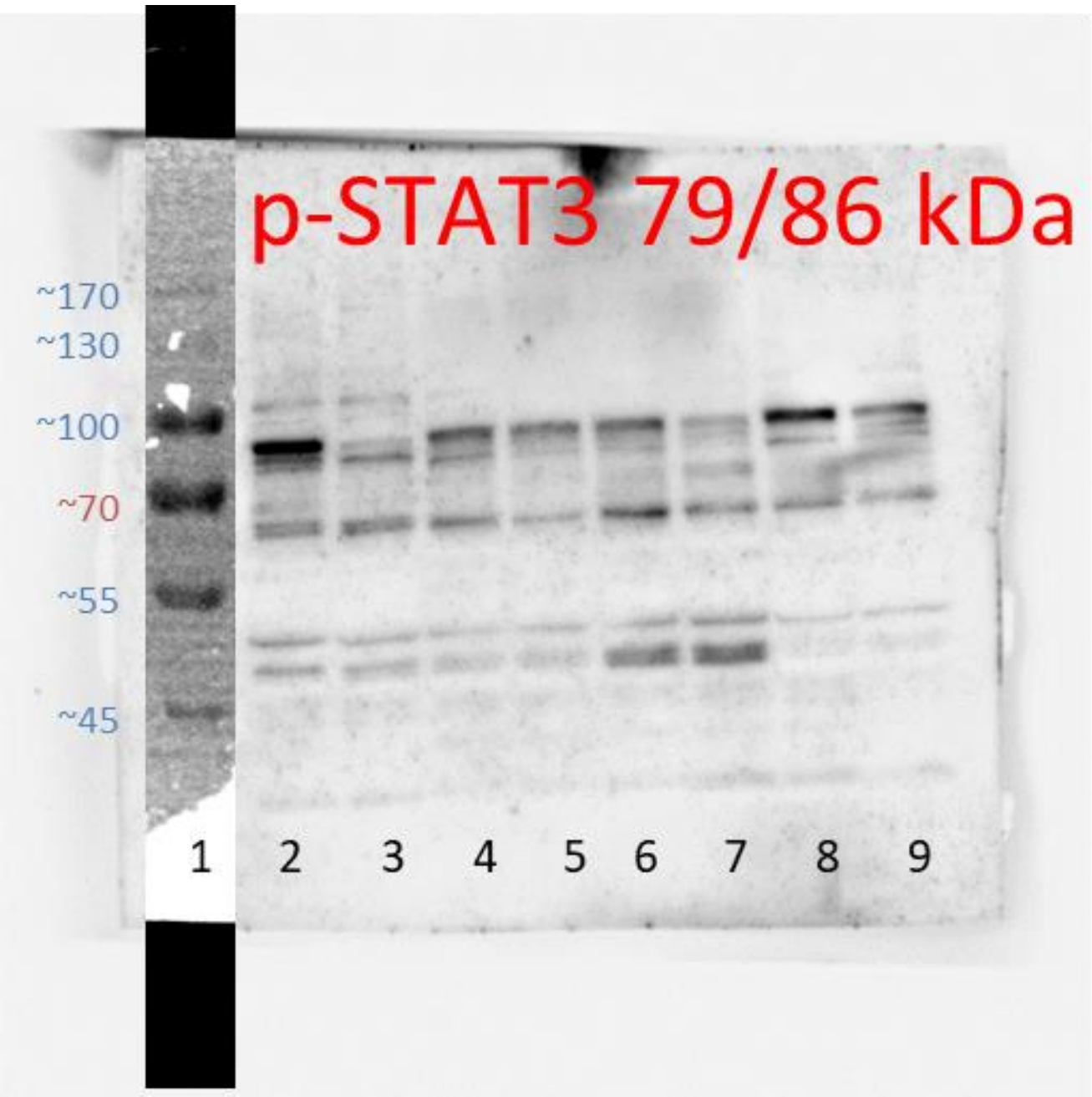

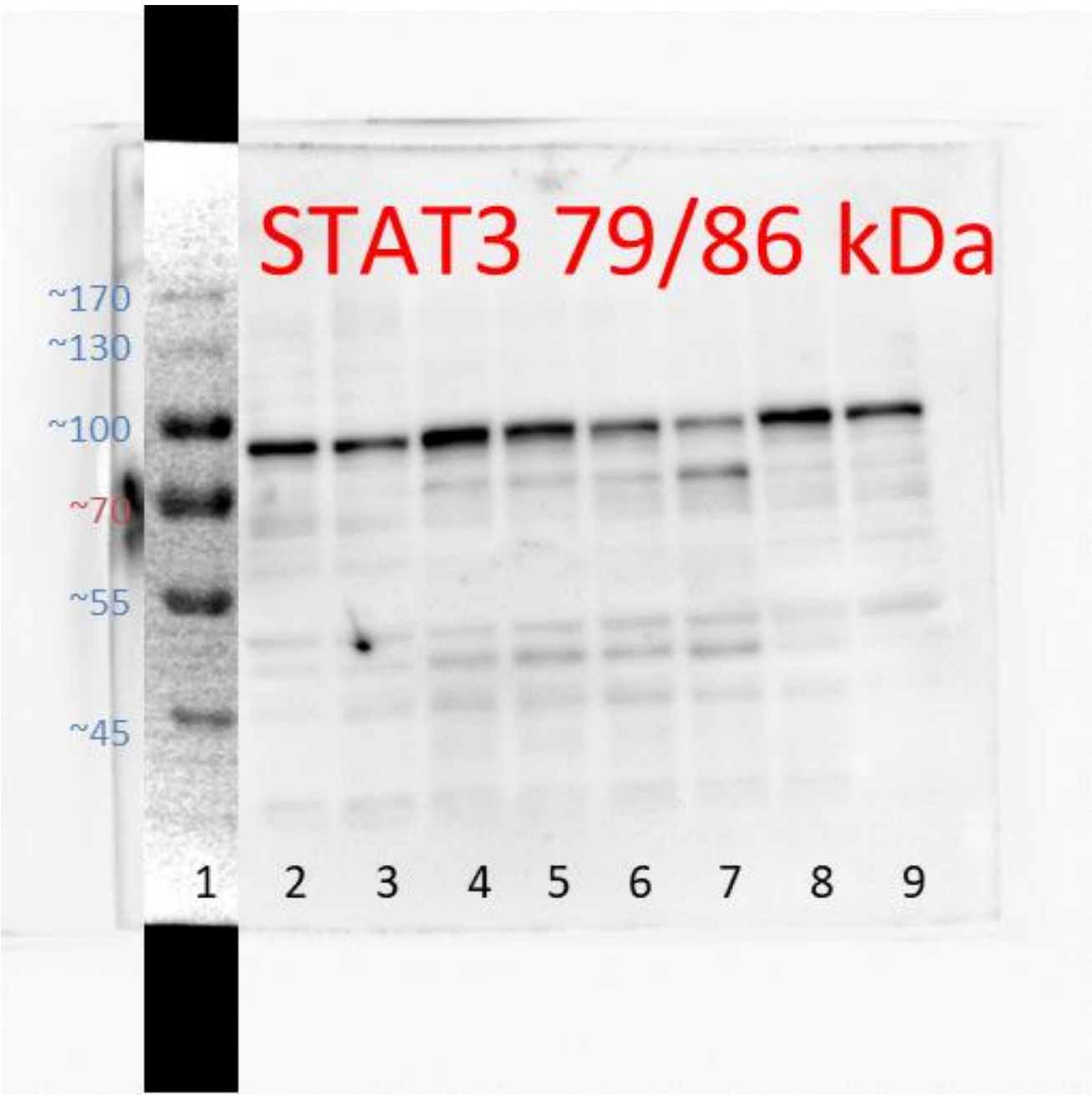

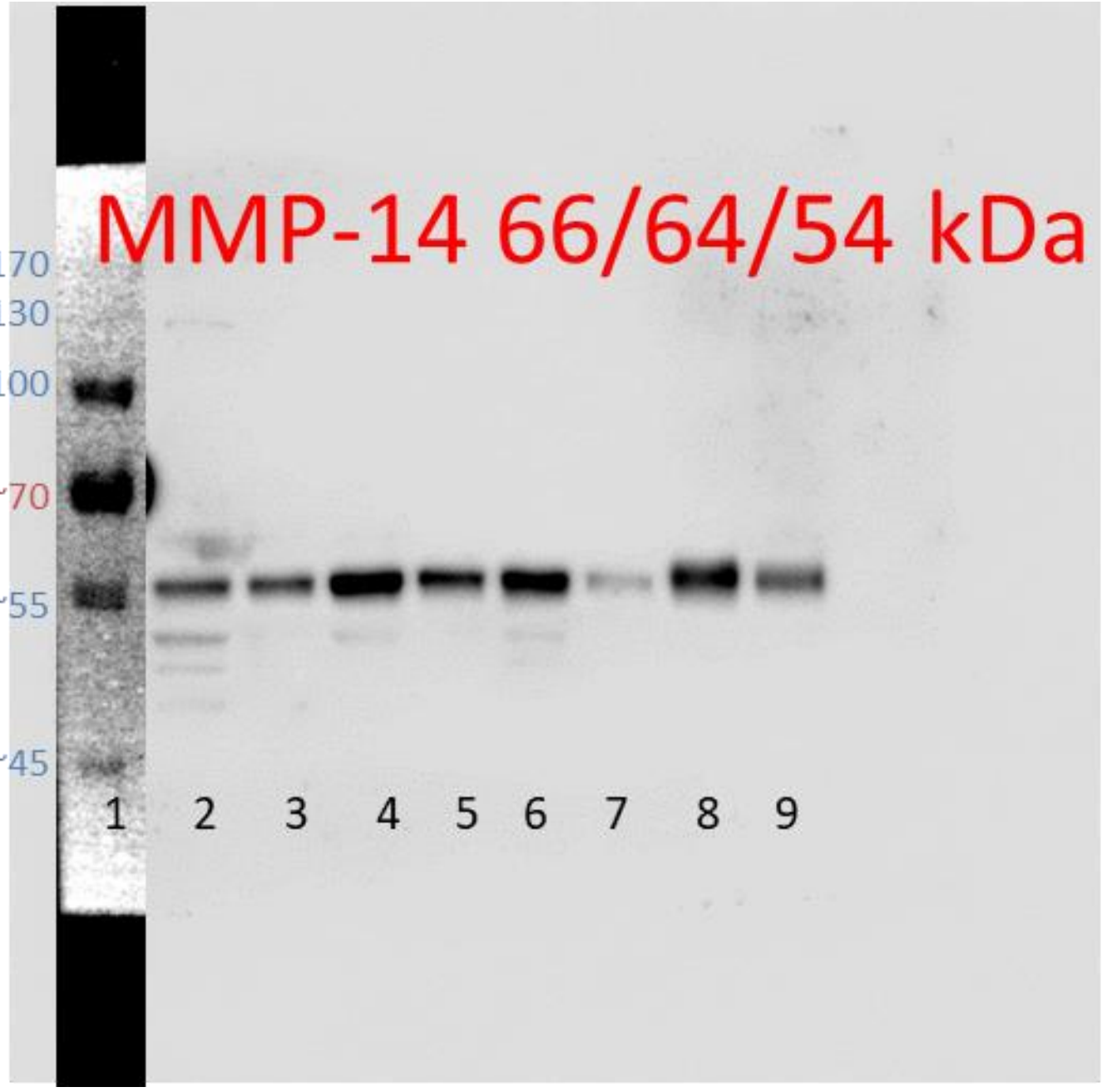

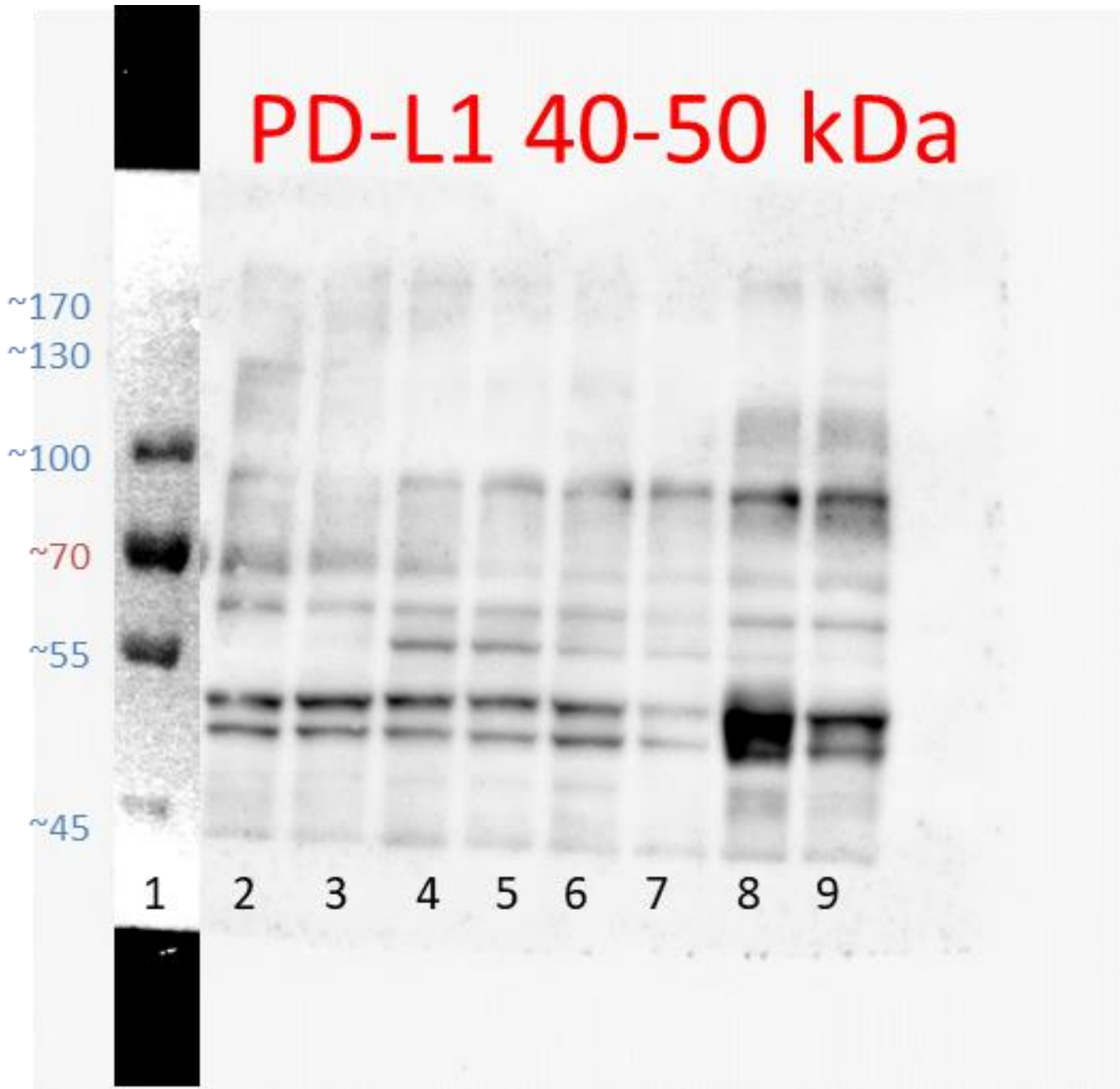

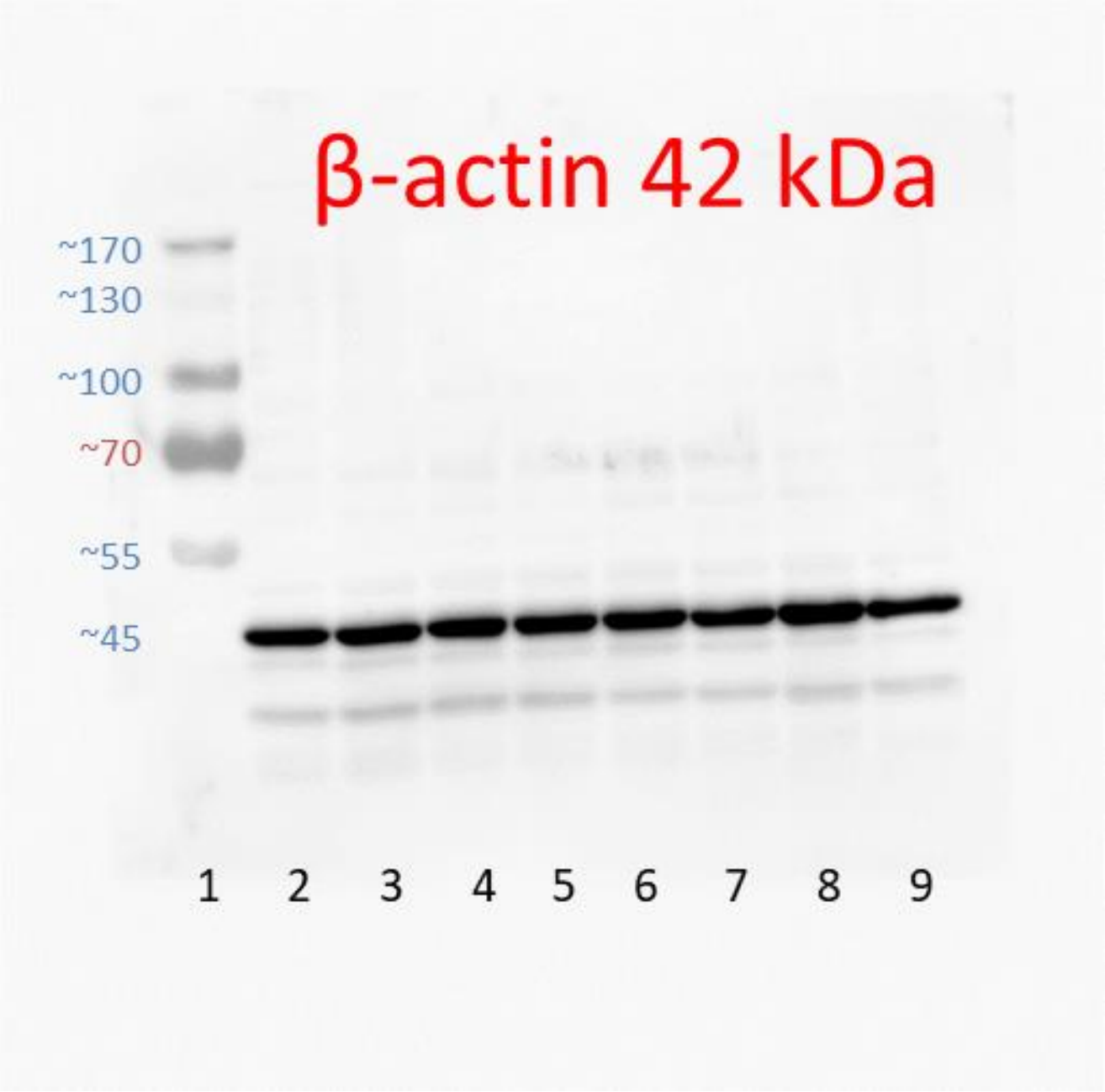

Original Western blots for Figure 5H, cell lines -/+ colivelin

Marker: PageRuler Prestained #26616 Thermo Scientific

1	Marker
2	UM-UC-3/57 control
3	UM-UC-3/57 colivelin
4	RT-4/ 42 control
5	RT-4/42 colivelin
6	RT-112/52 control
7	RT-112/52 colivelin
8	HT-1376/49 control
9	HT-1376/49 colivelin

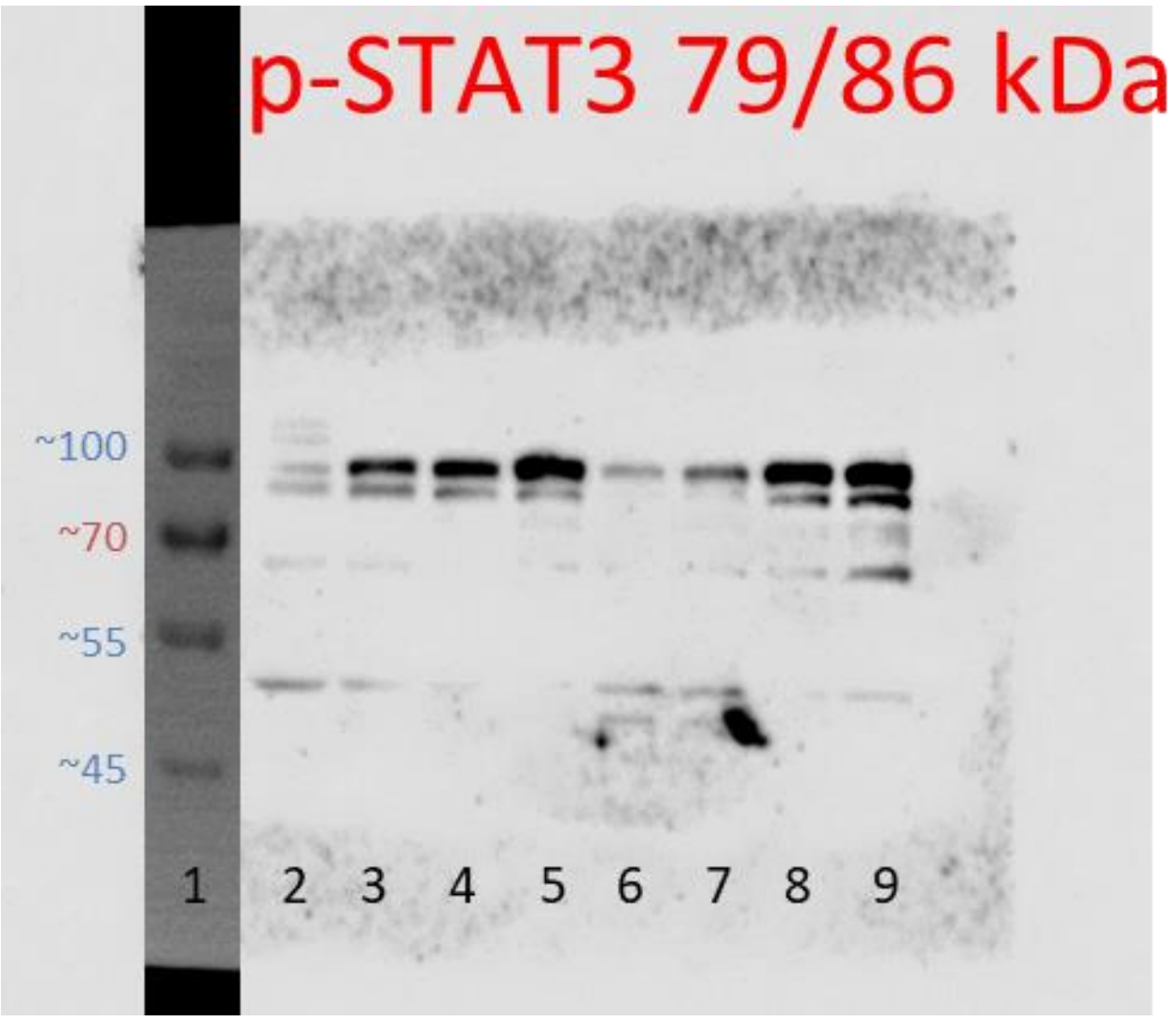

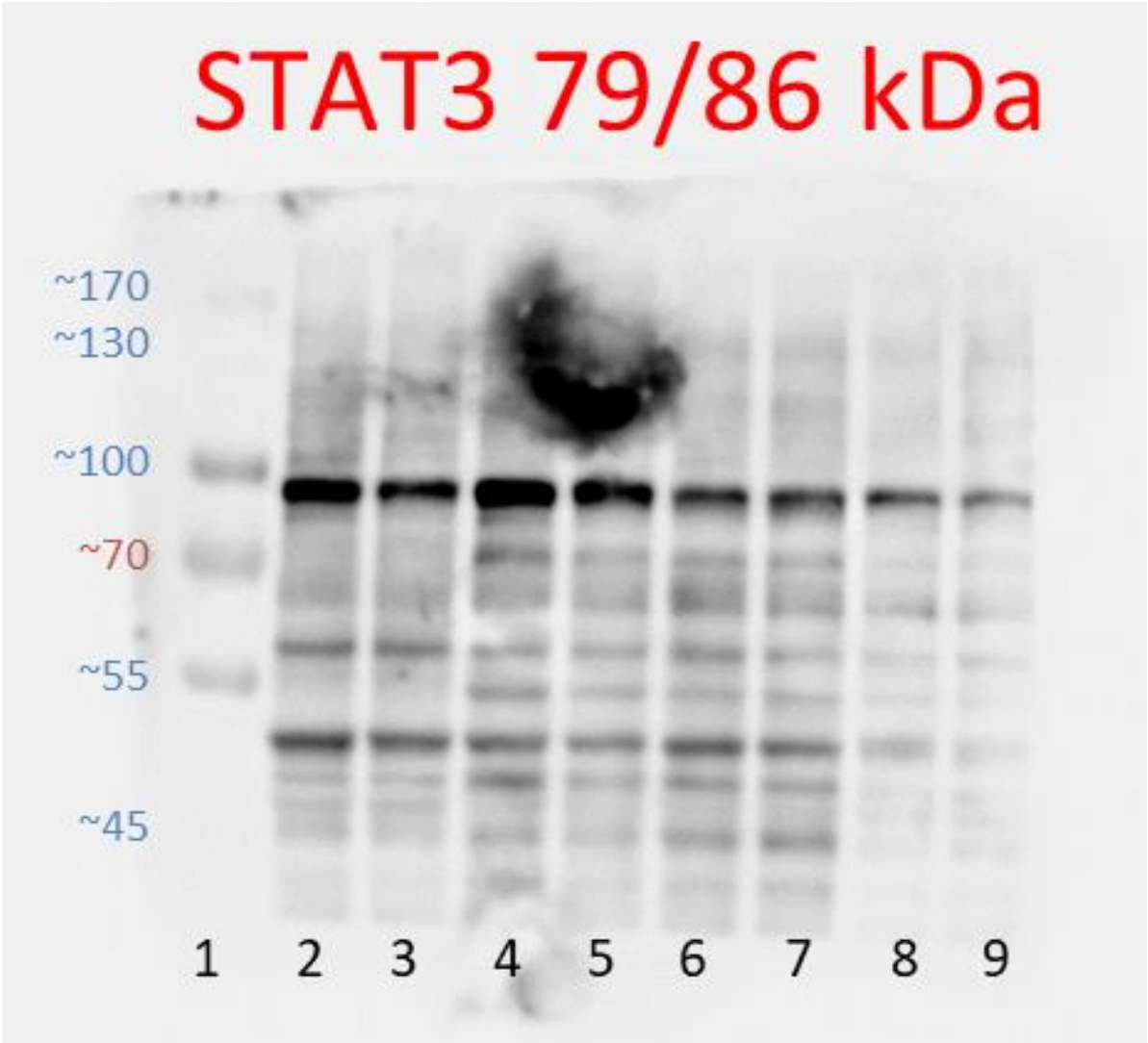

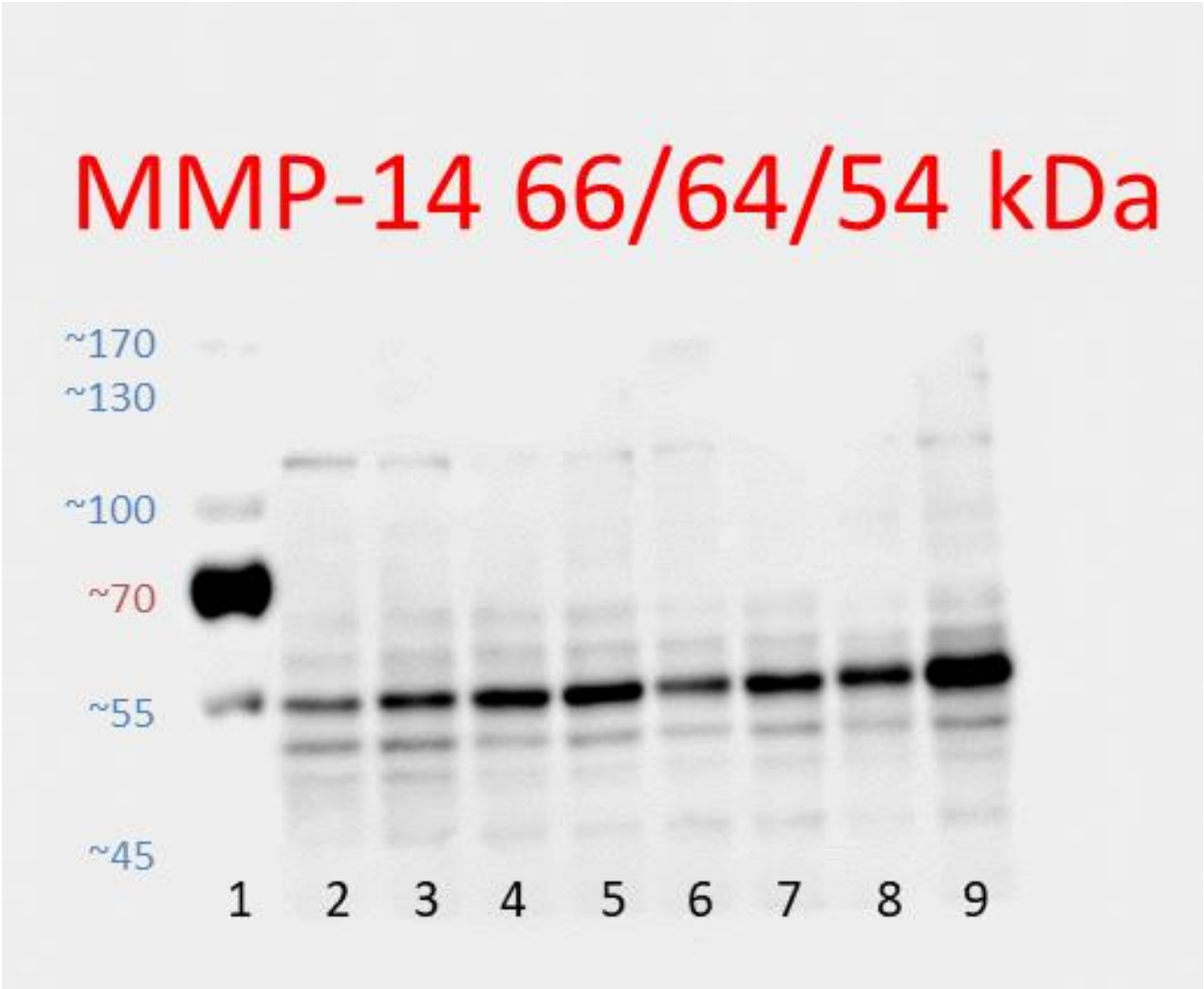

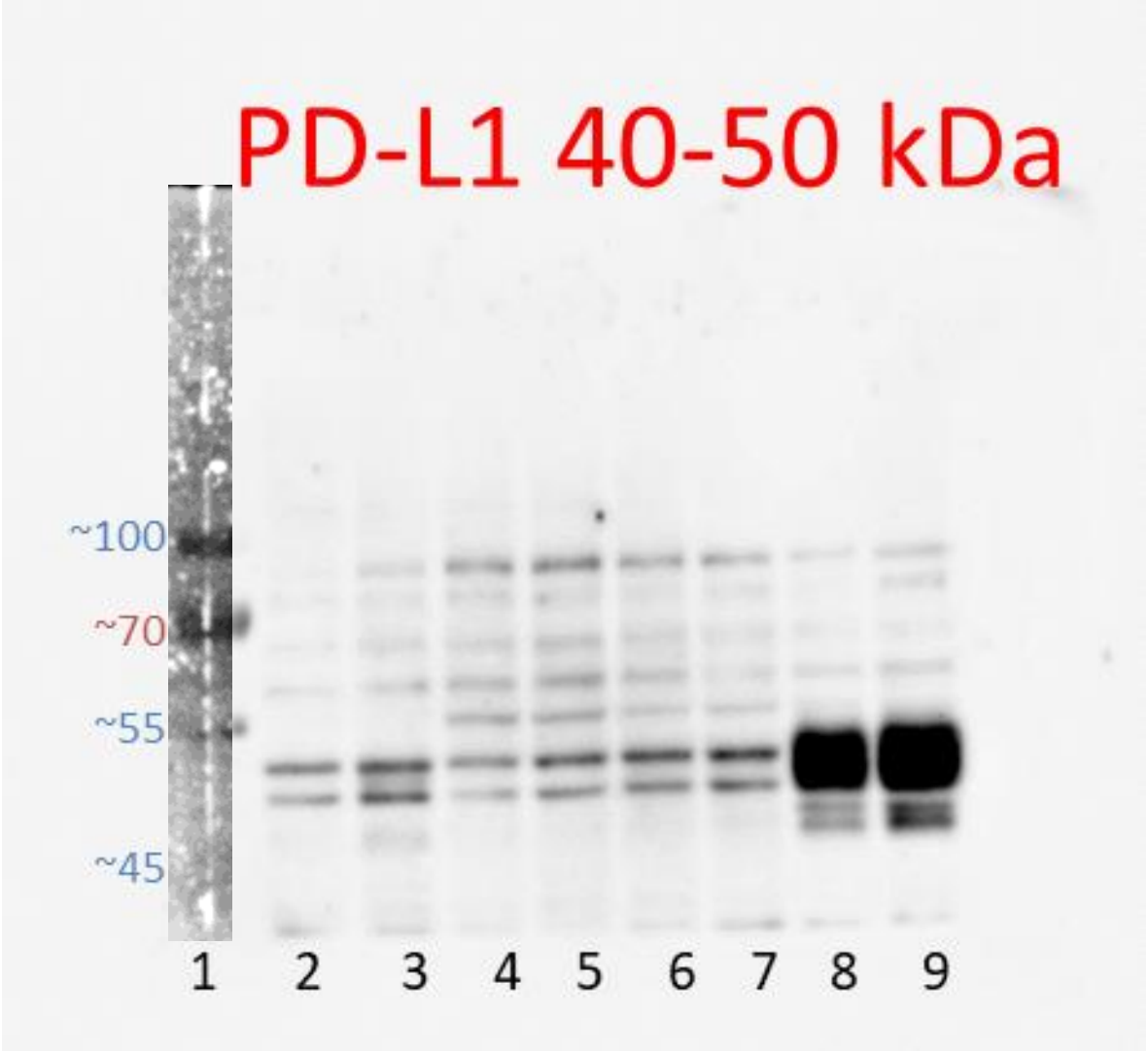

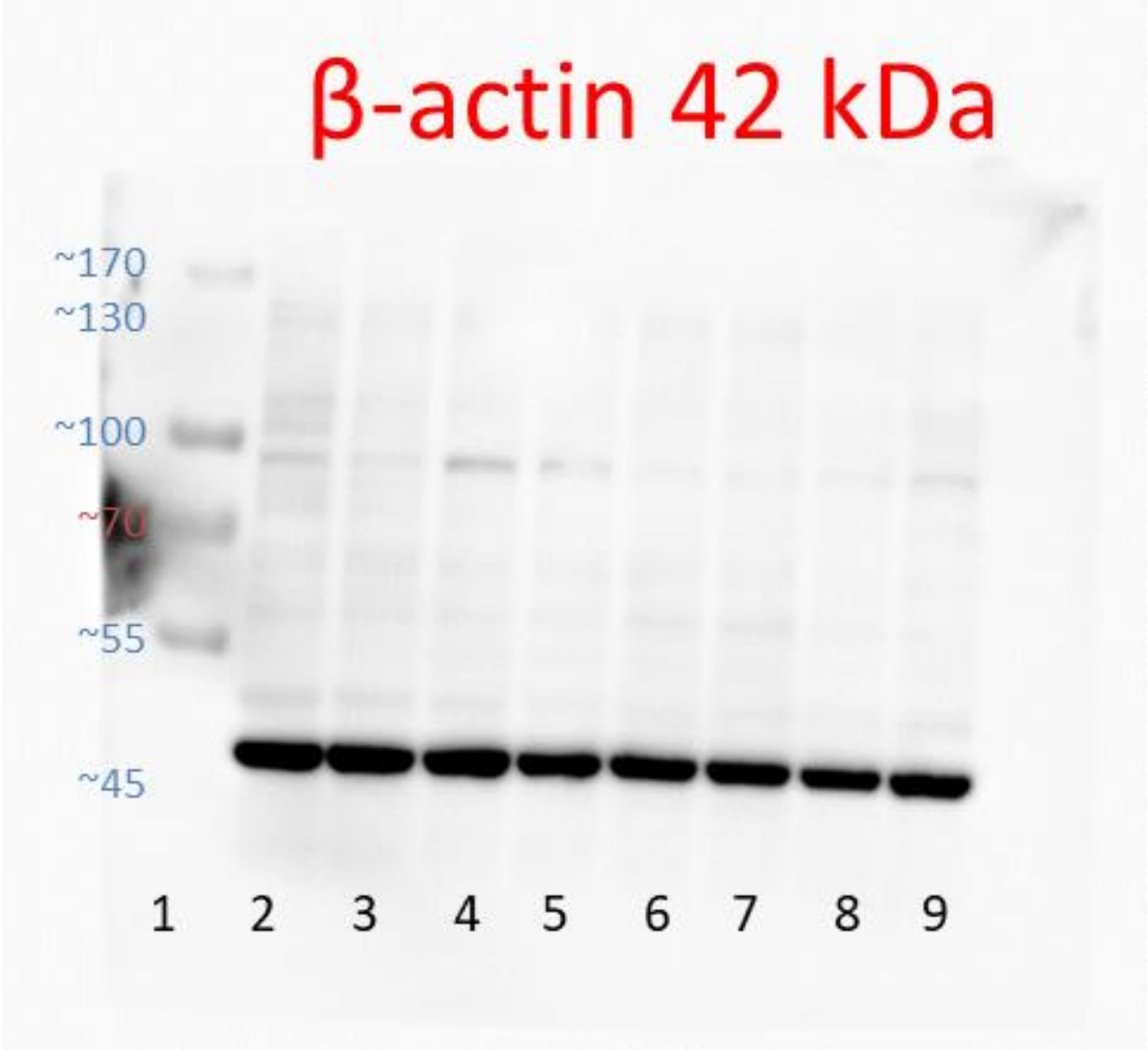

Original Western blots for Figure 6B, cell lines -/+ NSC405020

Marker: PageRuler Prestained #26616 Thermo Scientific

1	Marker
2	UM-UC-3/48 control
3	UM-UC-3/48 NSC
4	RT-4/42 control
5	RT-4/42 NSC
6	RT-112/46 control
7	RT-112/46 NSC
8	HT-1376/40 control
9	HT-1376/40 NSC

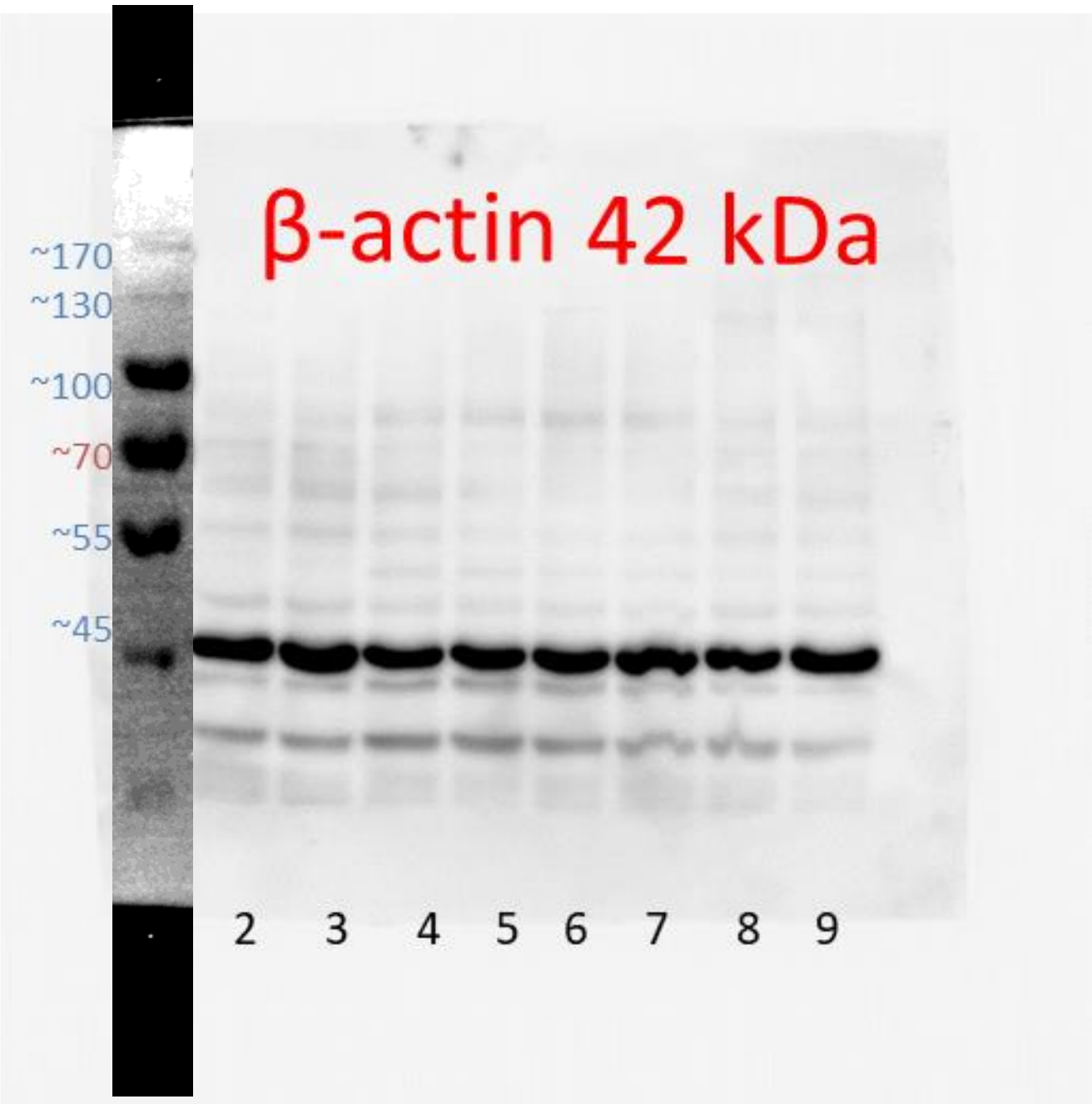

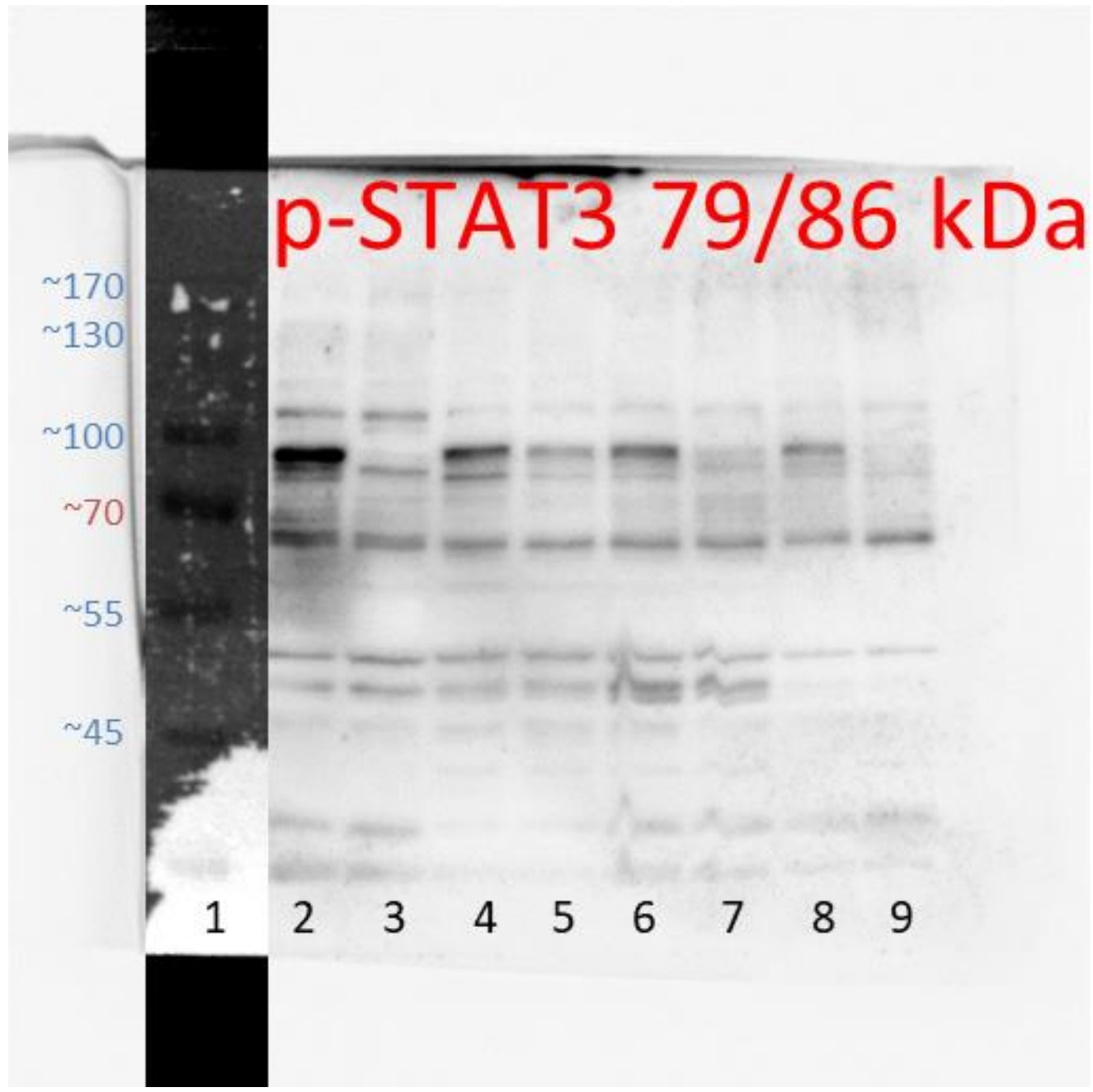

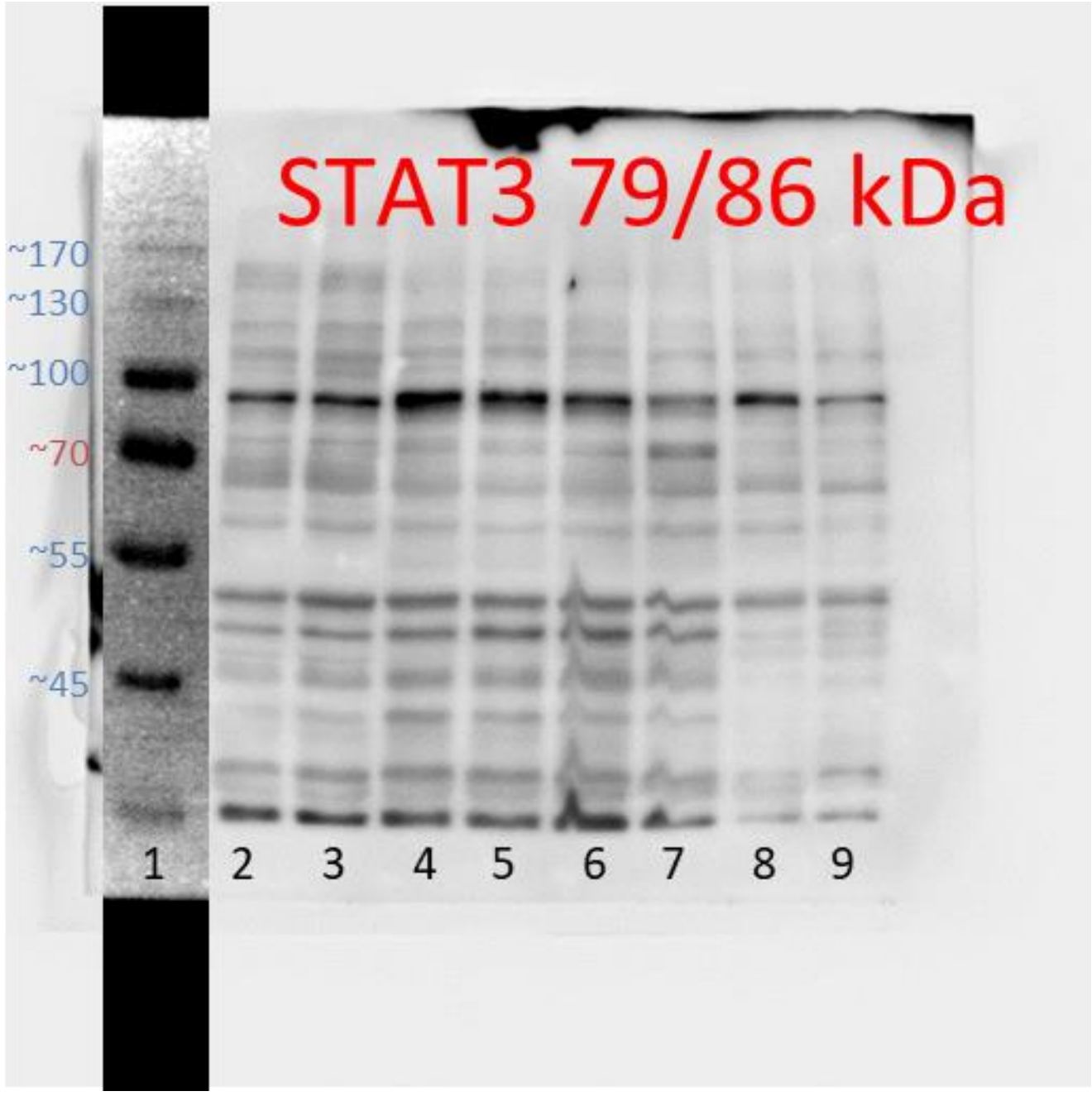

Original Western blots for Figure 6C, HT-1376 MMP-14 KD

Marker: PageRuler Prestained #26616 Thermo Scientific

1	Marker
2	HT control 1.2 2p
3	HT control 2.3 2p
4	HT knockdown 10b 1.1 2p
5	HT knockdown 10b 3.1 2p
6	HT knockdown 10b 3.2 2p
7	HT knockdown 10b 3.3 2p
8	HT knockdown 10b 3.4 2p
9	HT knockdown 10b 4.1 2p

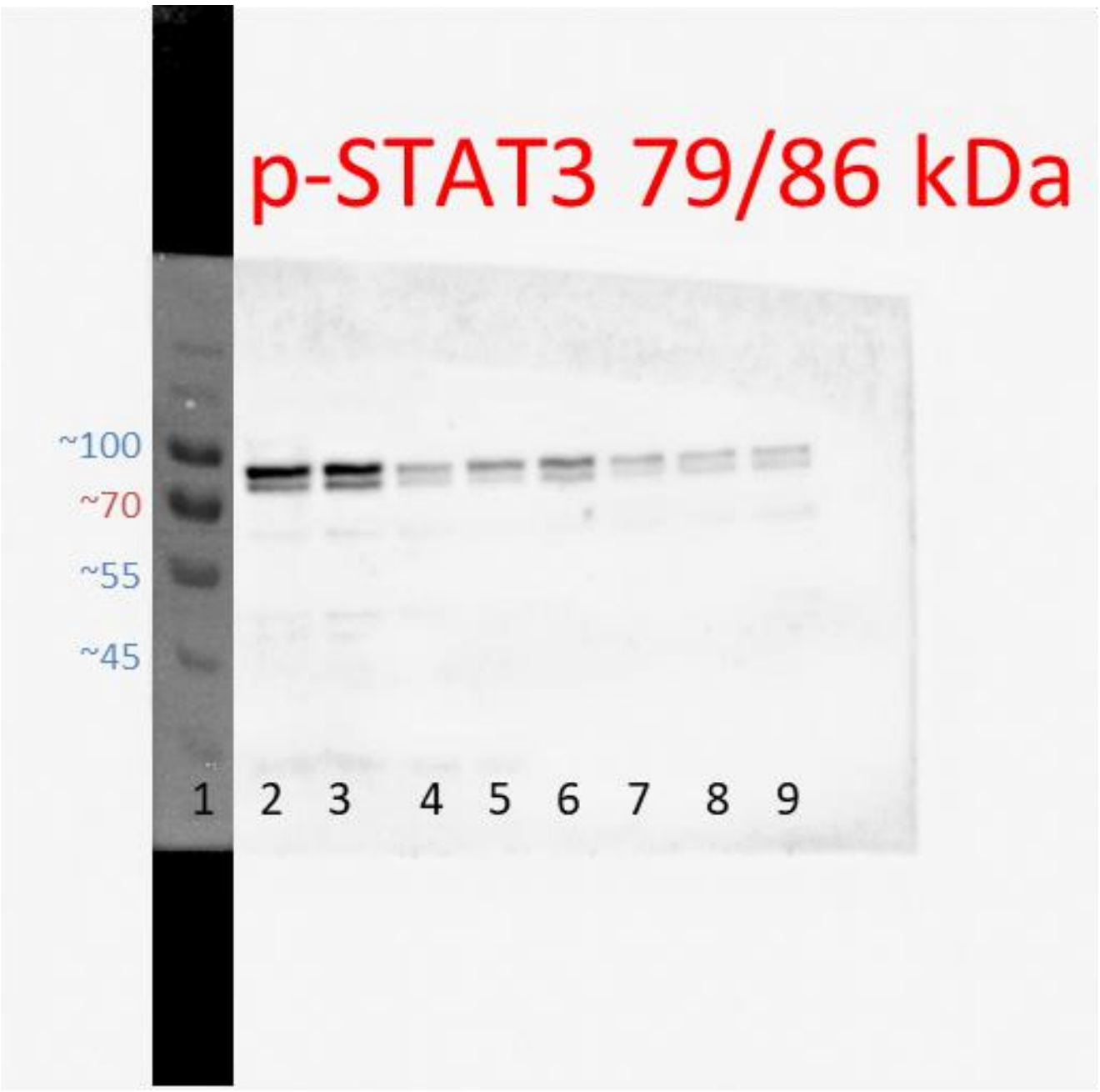

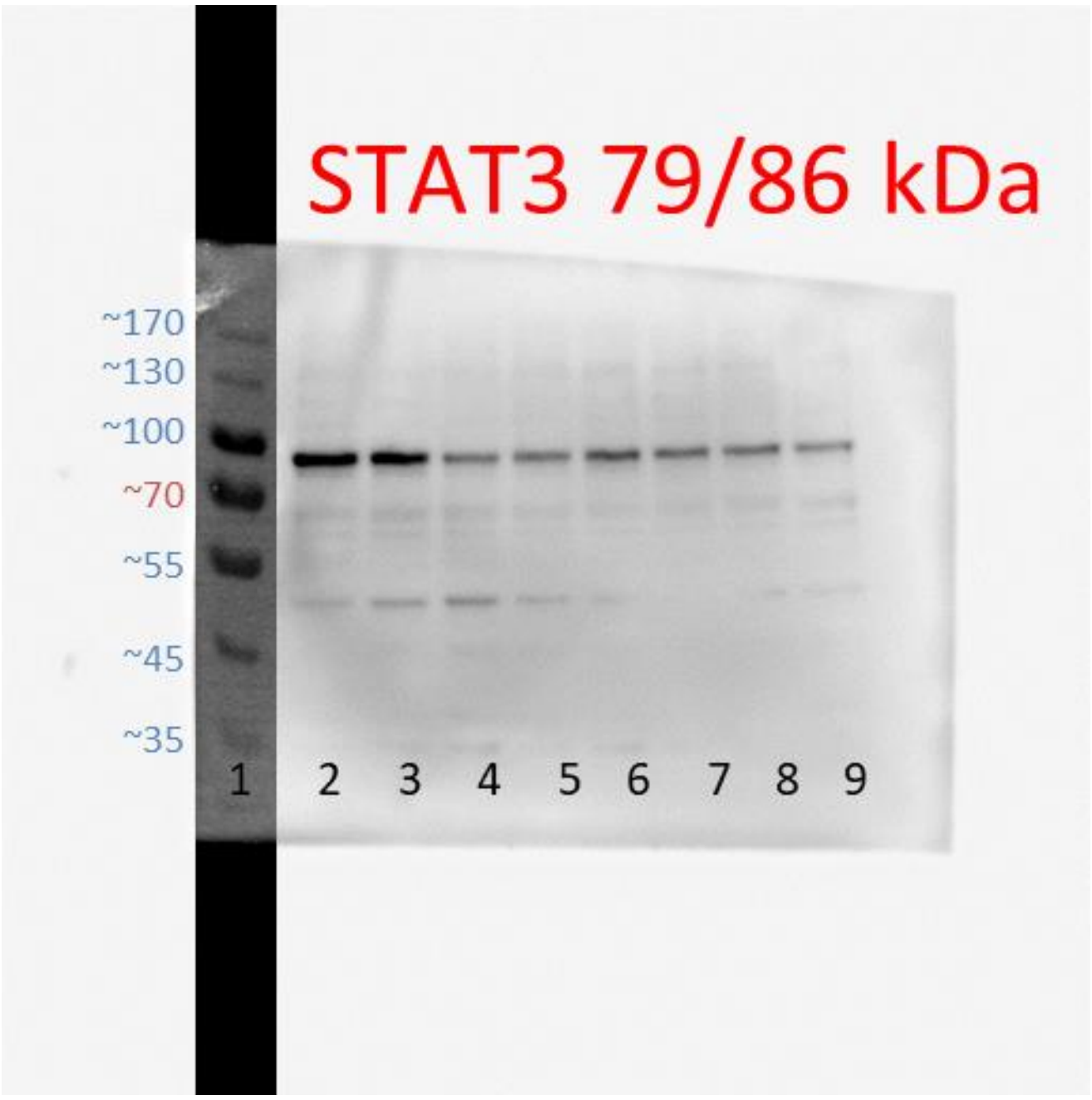

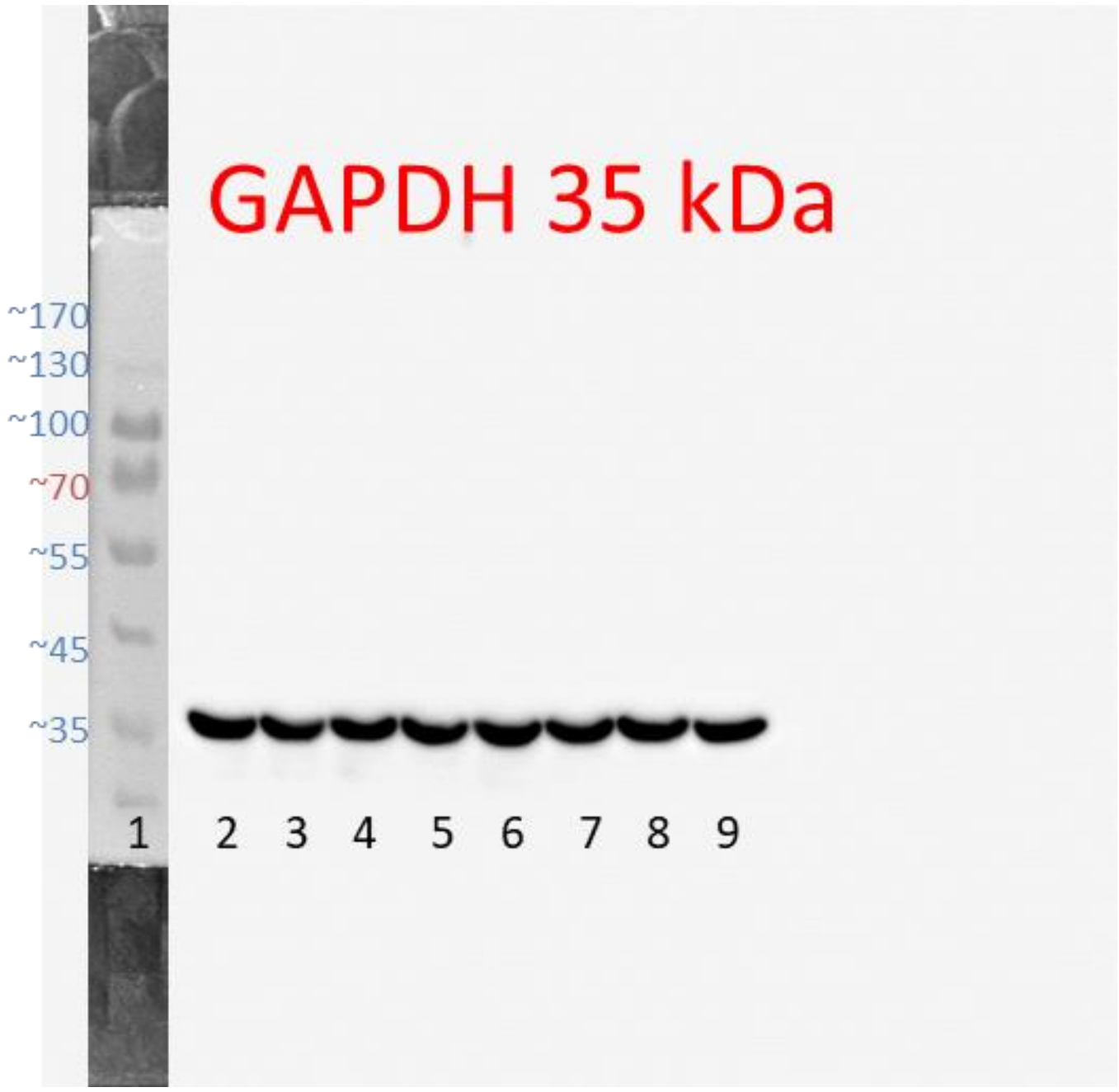

Original Western blots for Figure 6D, RT-112 MMP-14 OE

Marker: PageRuler Prestained #26616 Thermo Scientific

1	Marker	
2	RT-112/vector 1 5p	
3	RT-112/vector 2 5p	
4	RT-112/OE 1 5p	
5	RT-112/OE 2 5p	
6	RT-112/OE 3 5p	
7	RT-112/OE 4 5p	

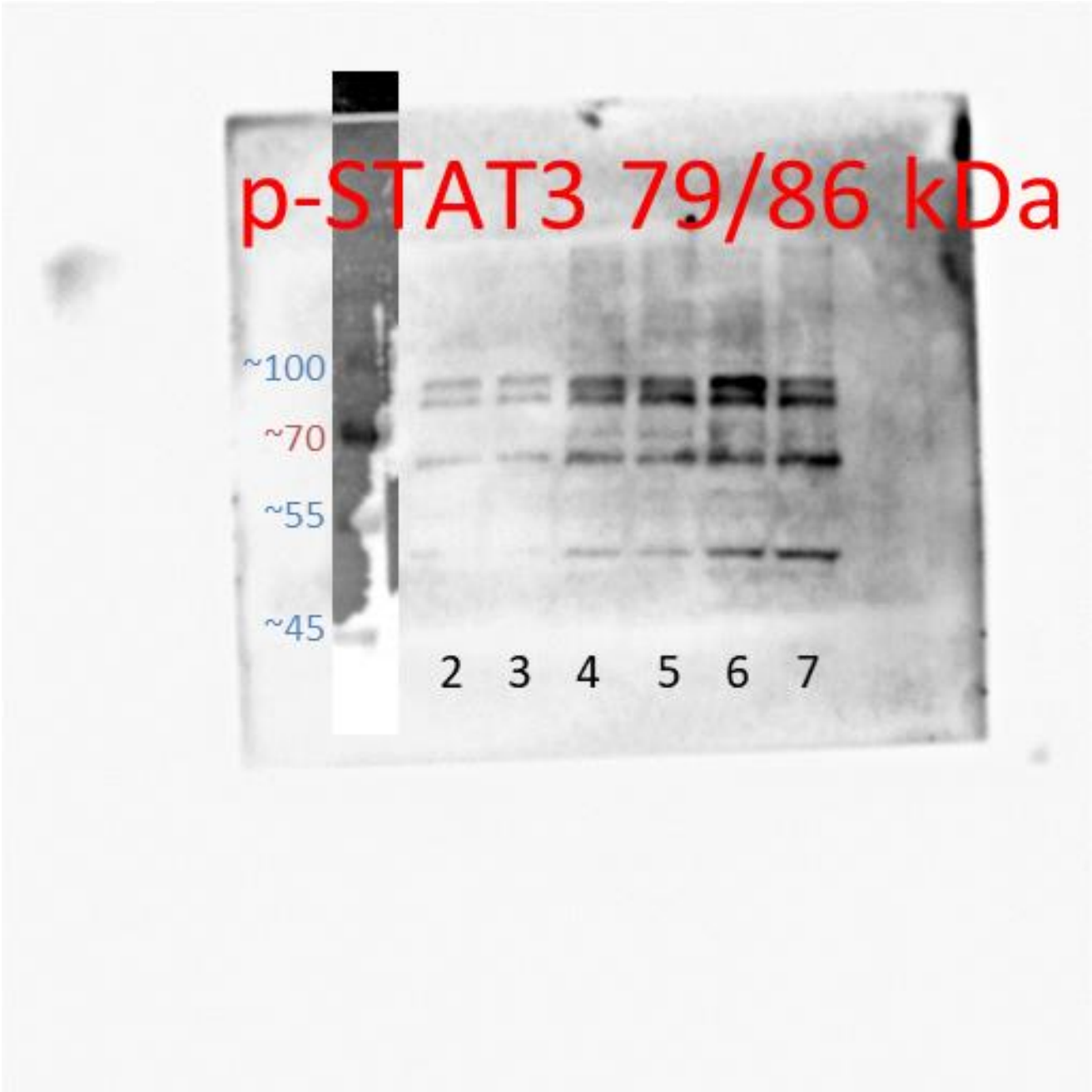

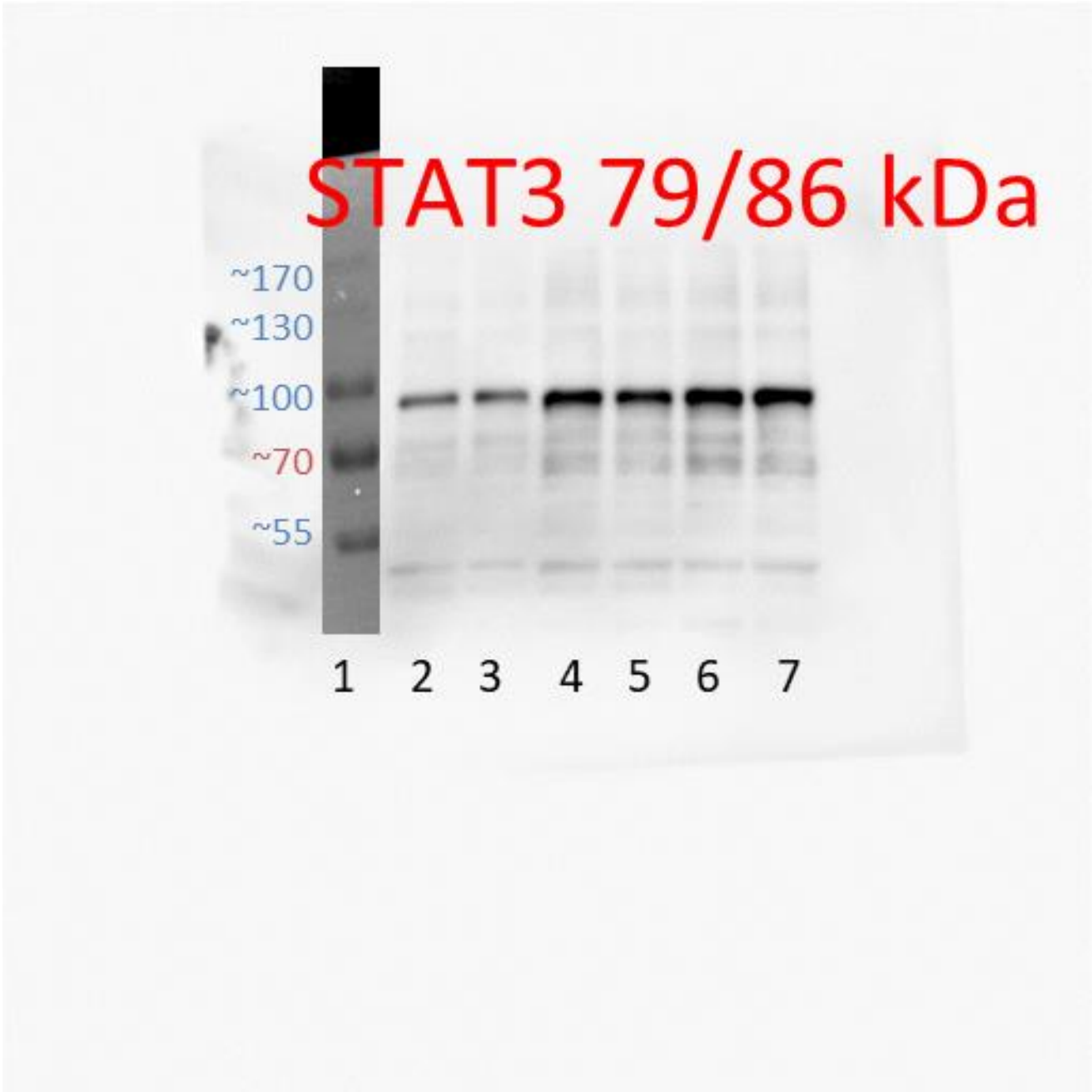

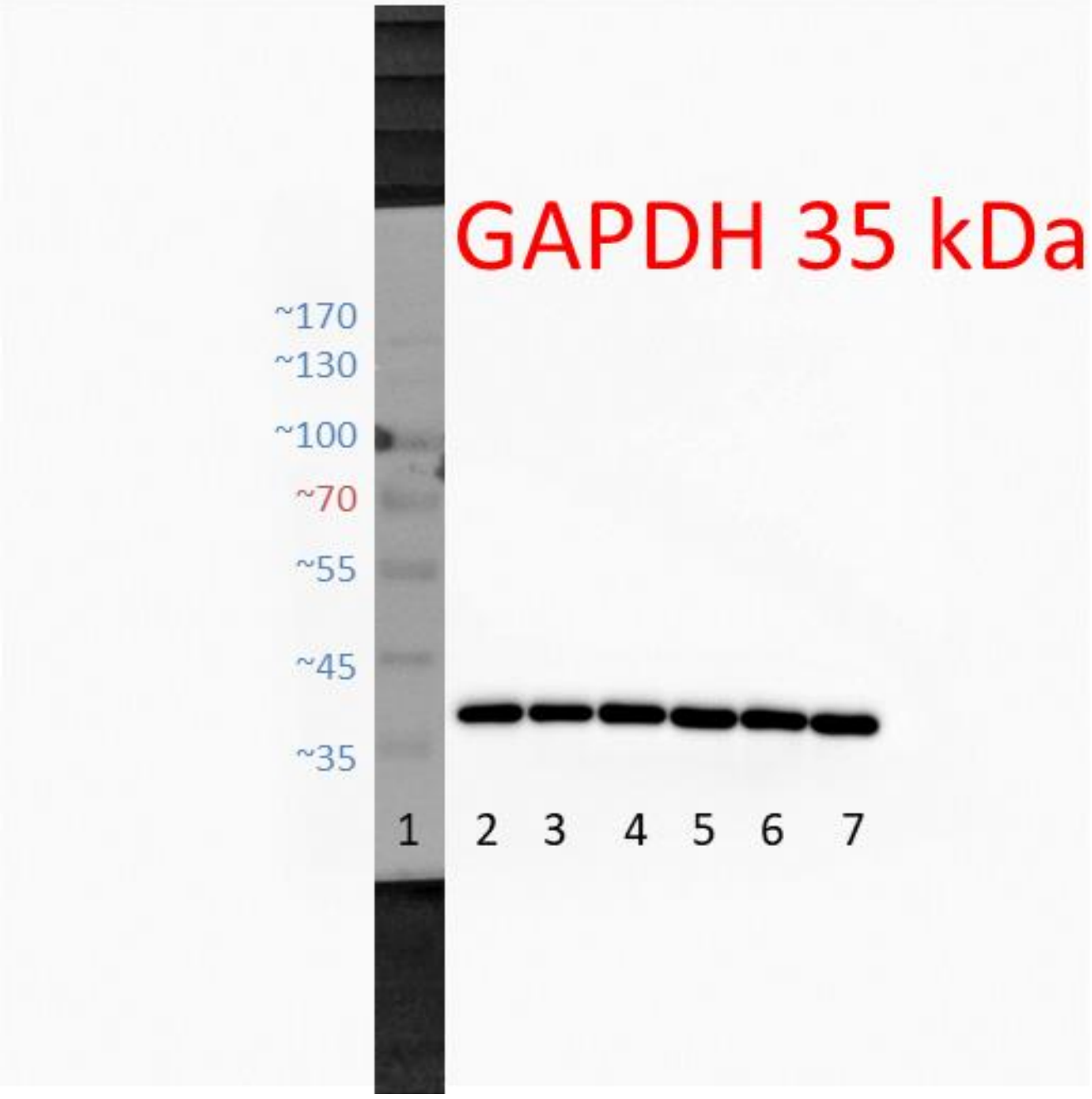

Original Western blots for Figure 6F, HT-1376 IP

Marker: PageRuler (Plus) Prestained #26619 (PD-L1, GAPDH), #26616 (MMP-14) Thermo Scientific

1	Marker
2	IP Lysis buffer Input
3	Rabbit lg G negative control
4	IP Lysis buffer IP

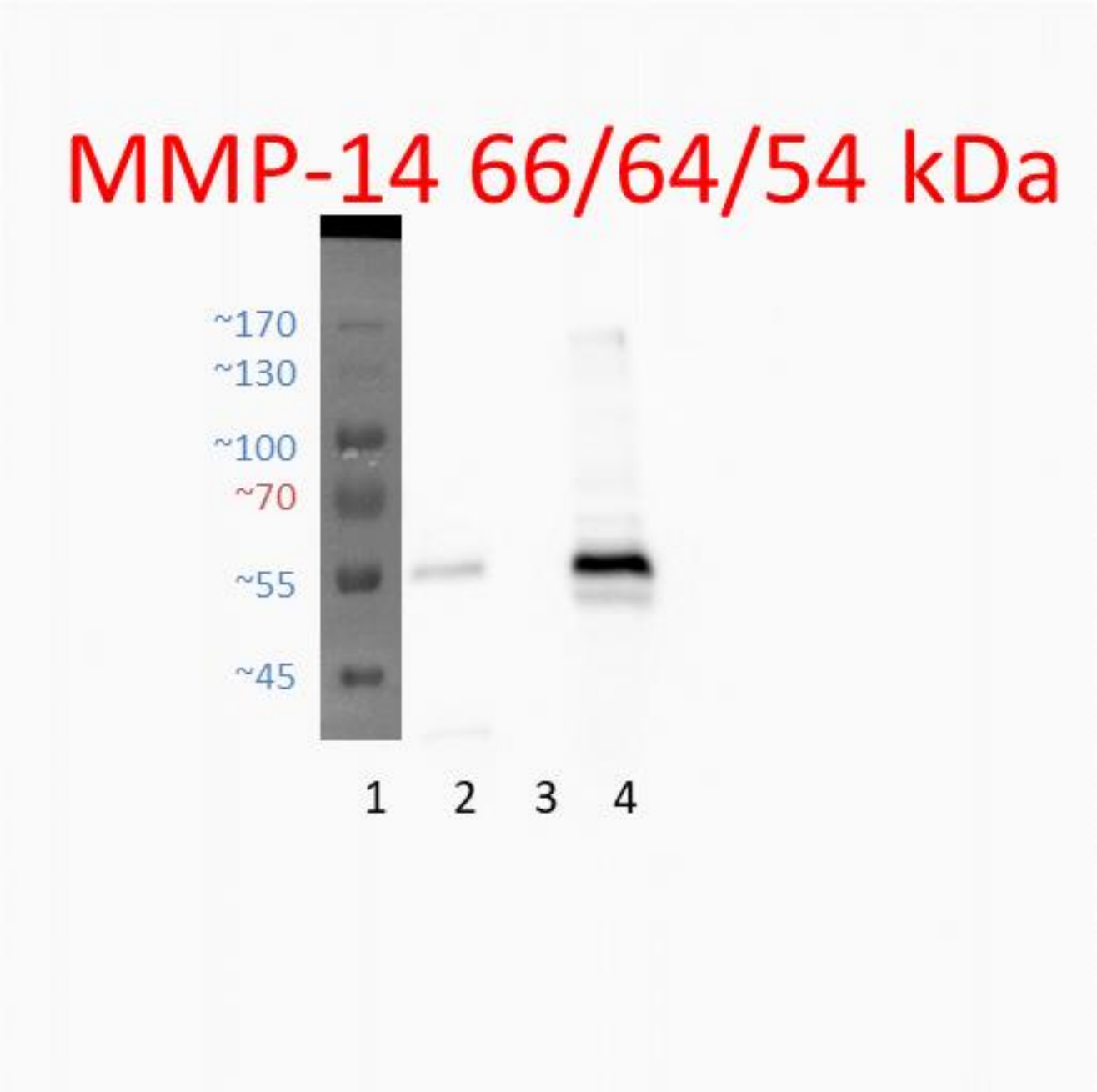

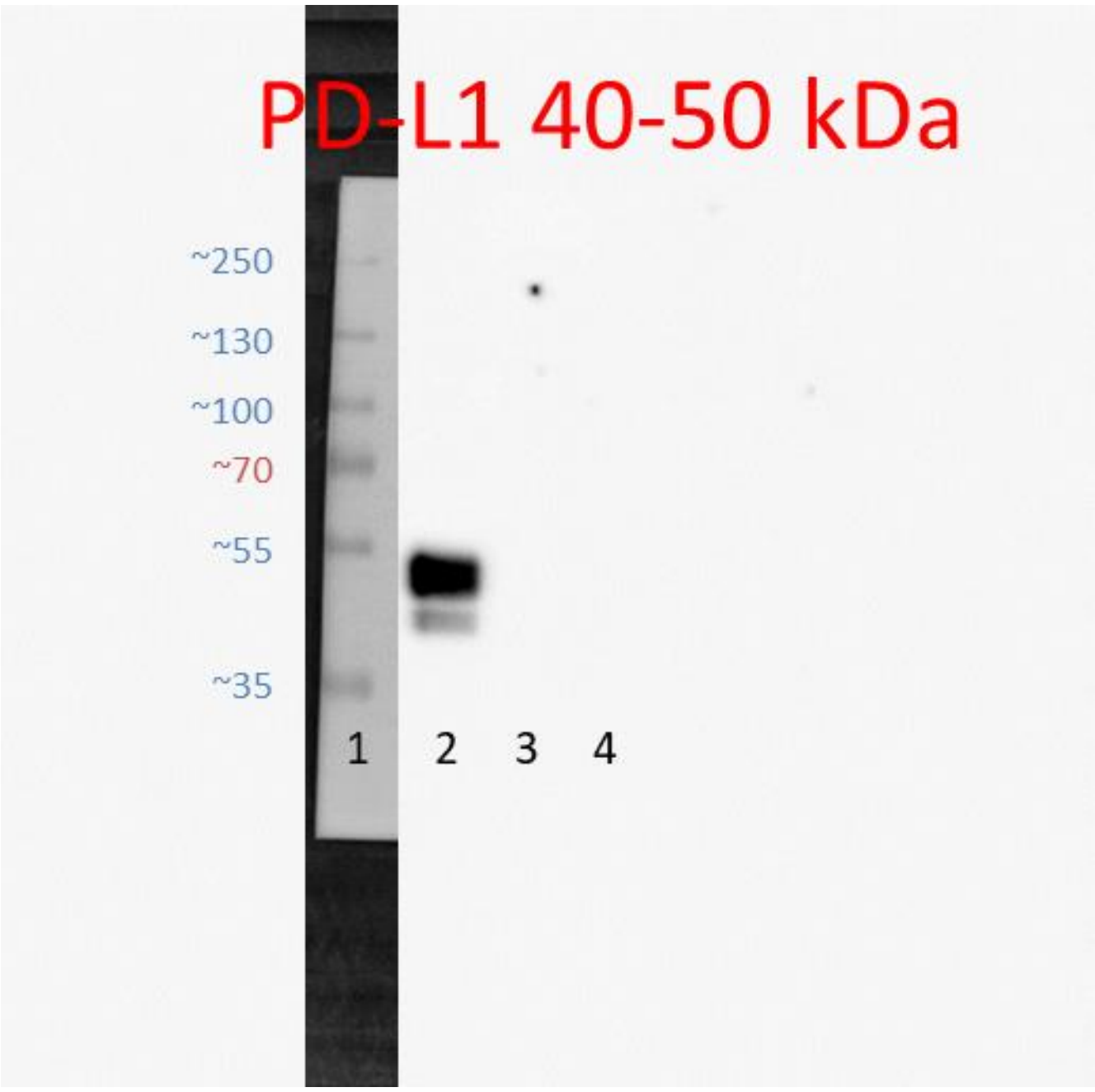

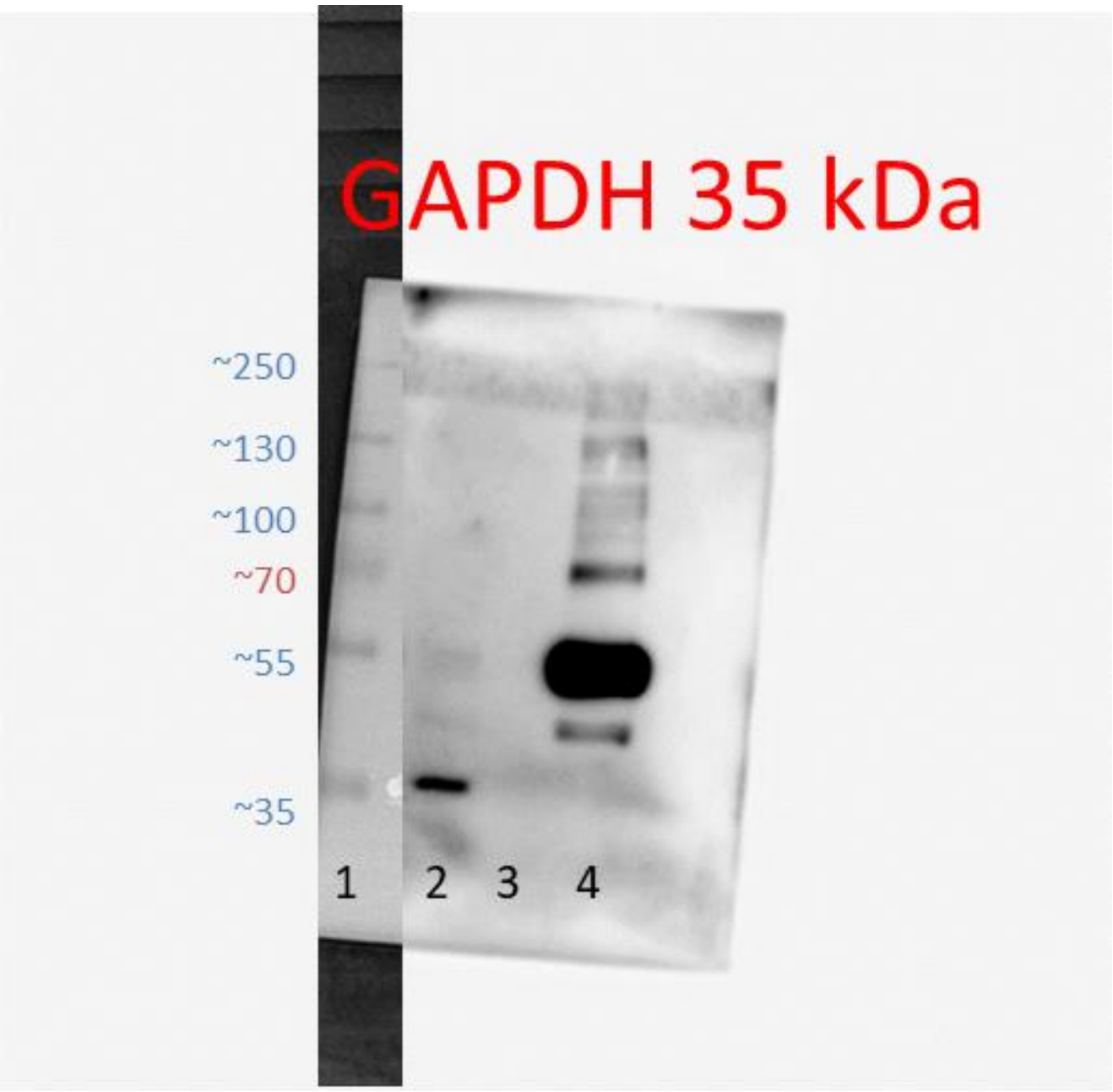

Original Western blots for Figure 6G, HT-1376 IP

Marker: PageRuler Plus Prestained #26616 Thermo Scientific

1	Marker
2	IP Lysis buffer Input
3	Rabbit Ig G negative control
4	IP Lysis buffer IP

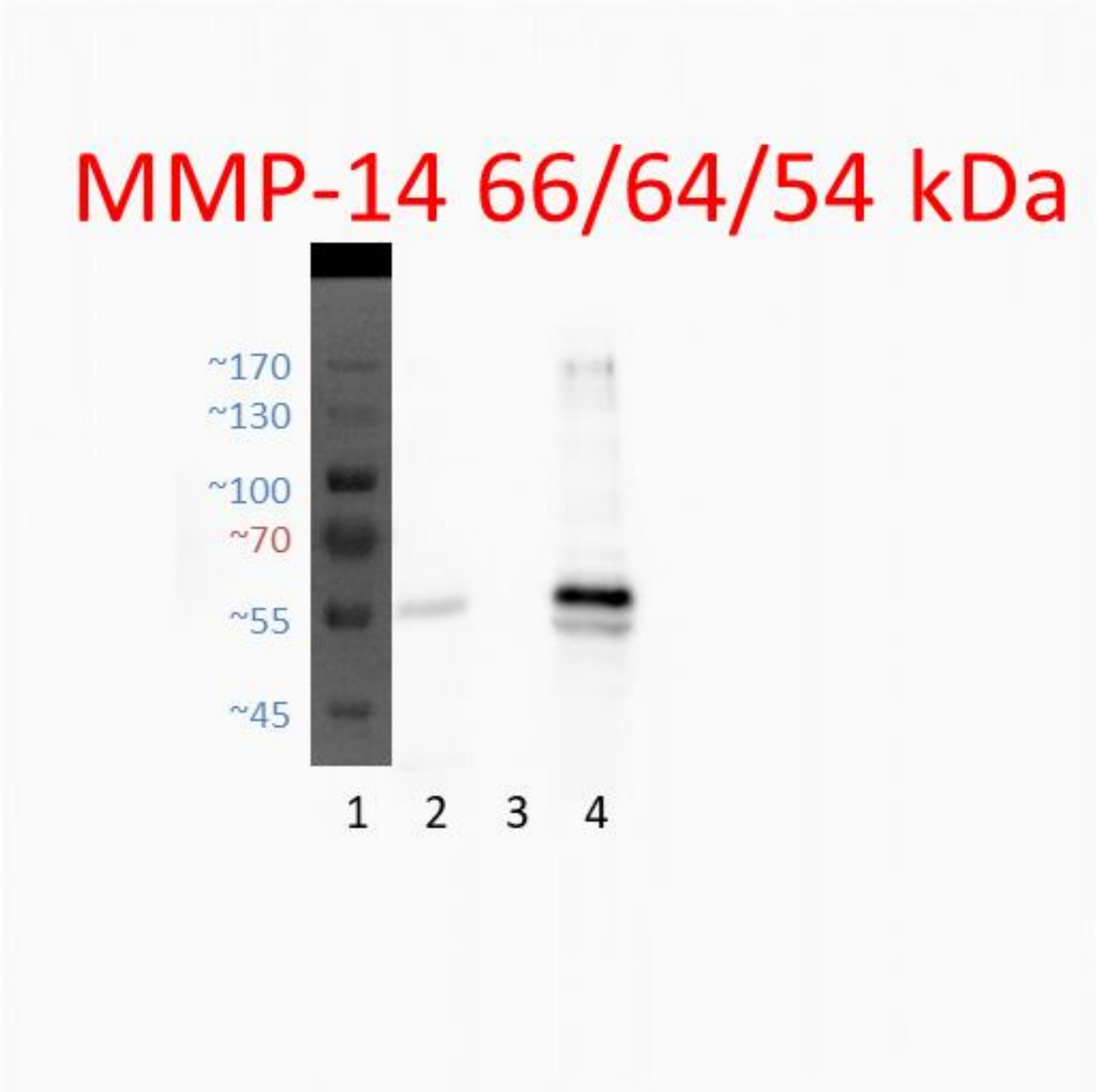

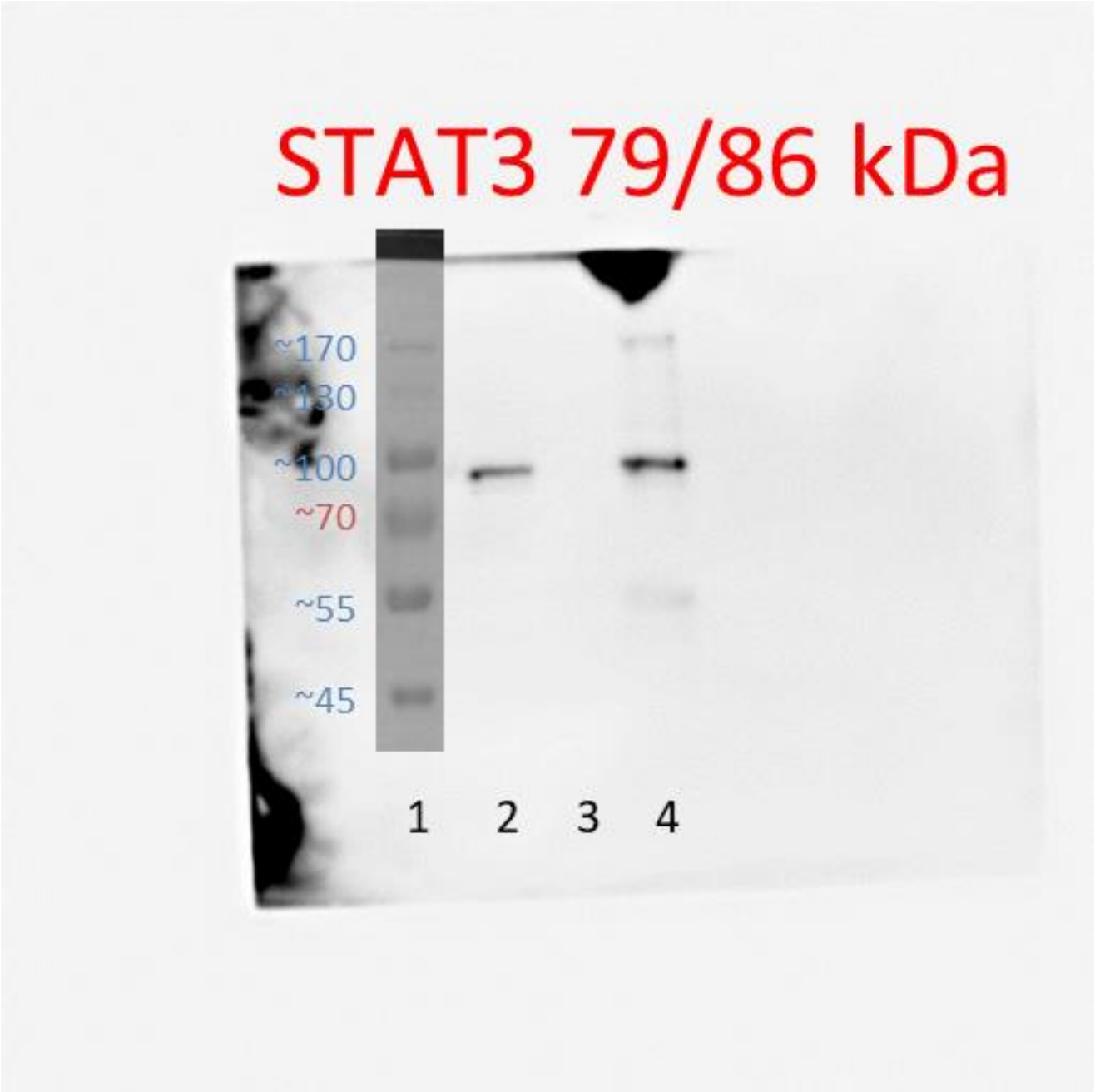

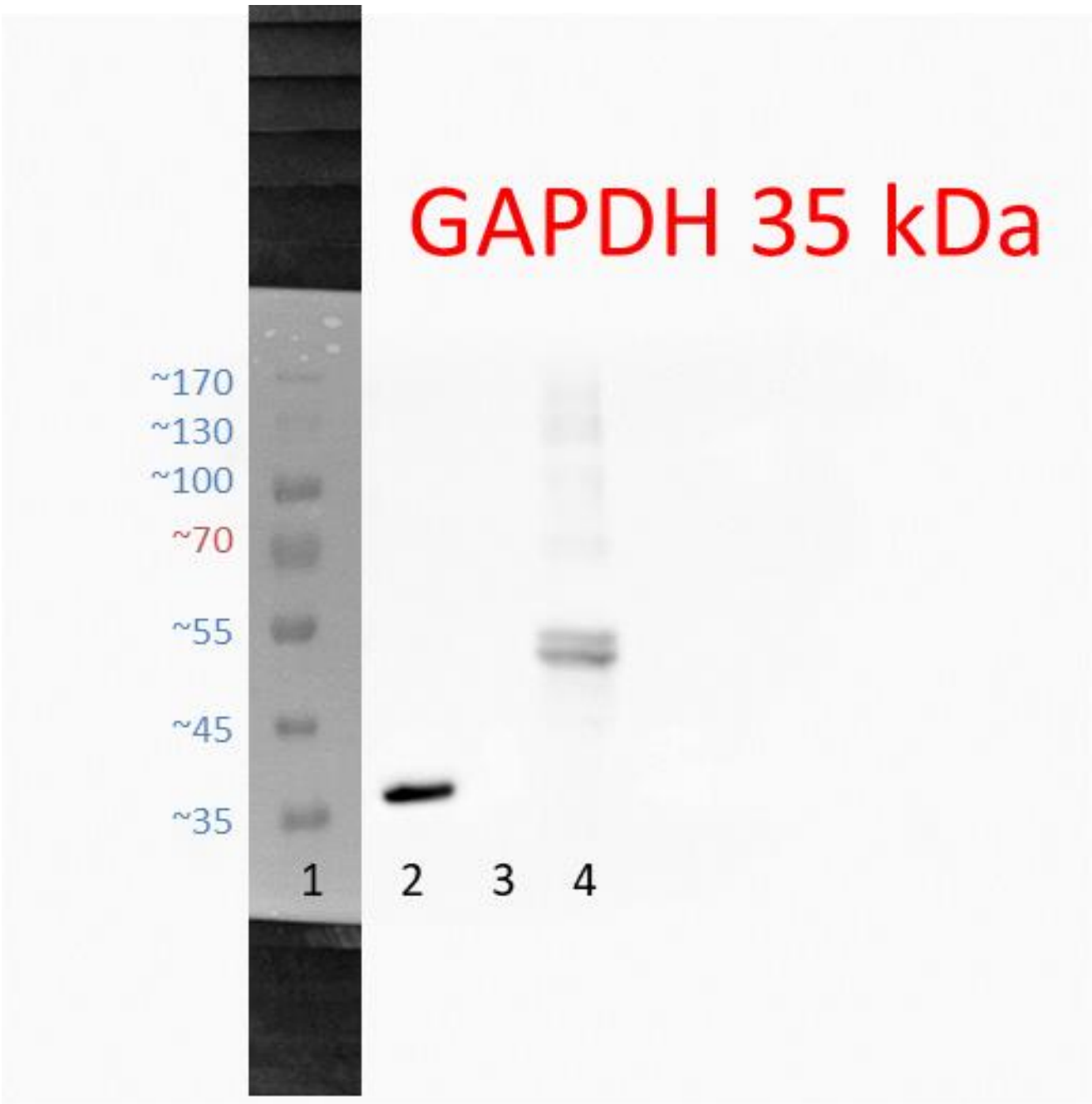

Original Western blots for Figure 6G, RT-112 IP

Marker: PageRuler Plus Prestained #26616 Thermo Scientific

1	Marker
2	IP Lysis buffer Input
3	Rabbit lg G negative control
4	IP Lysis buffer IP

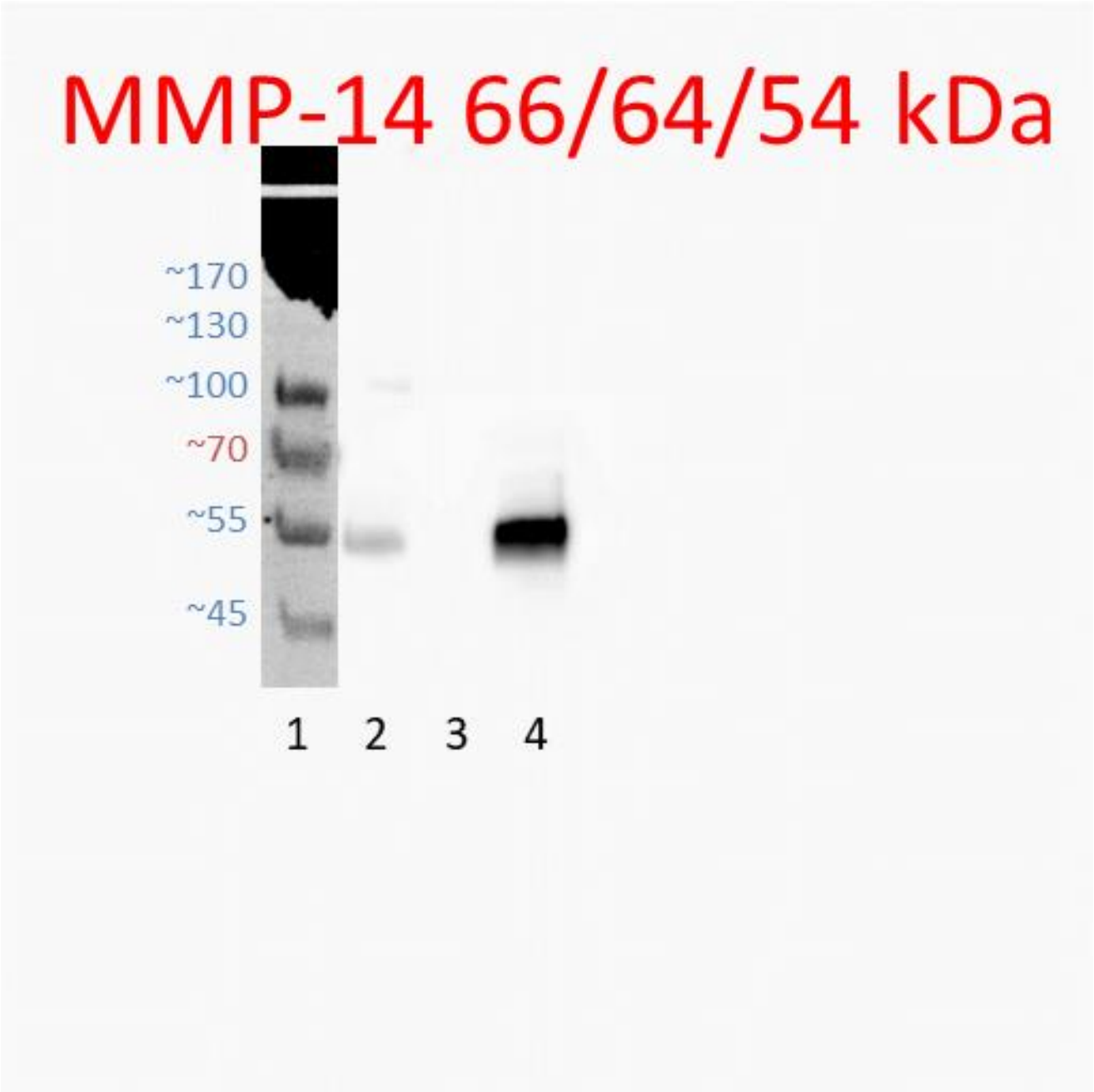

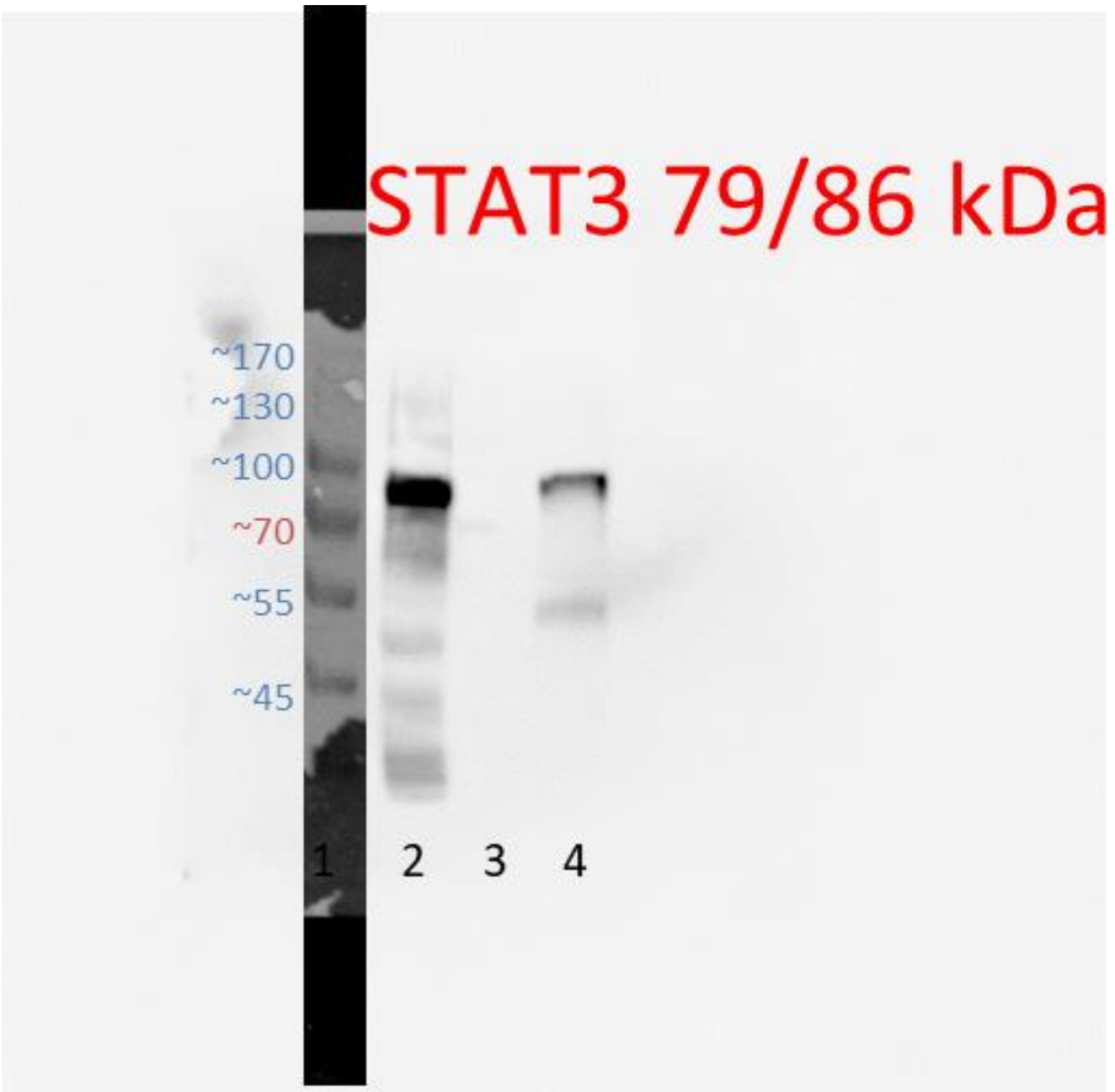

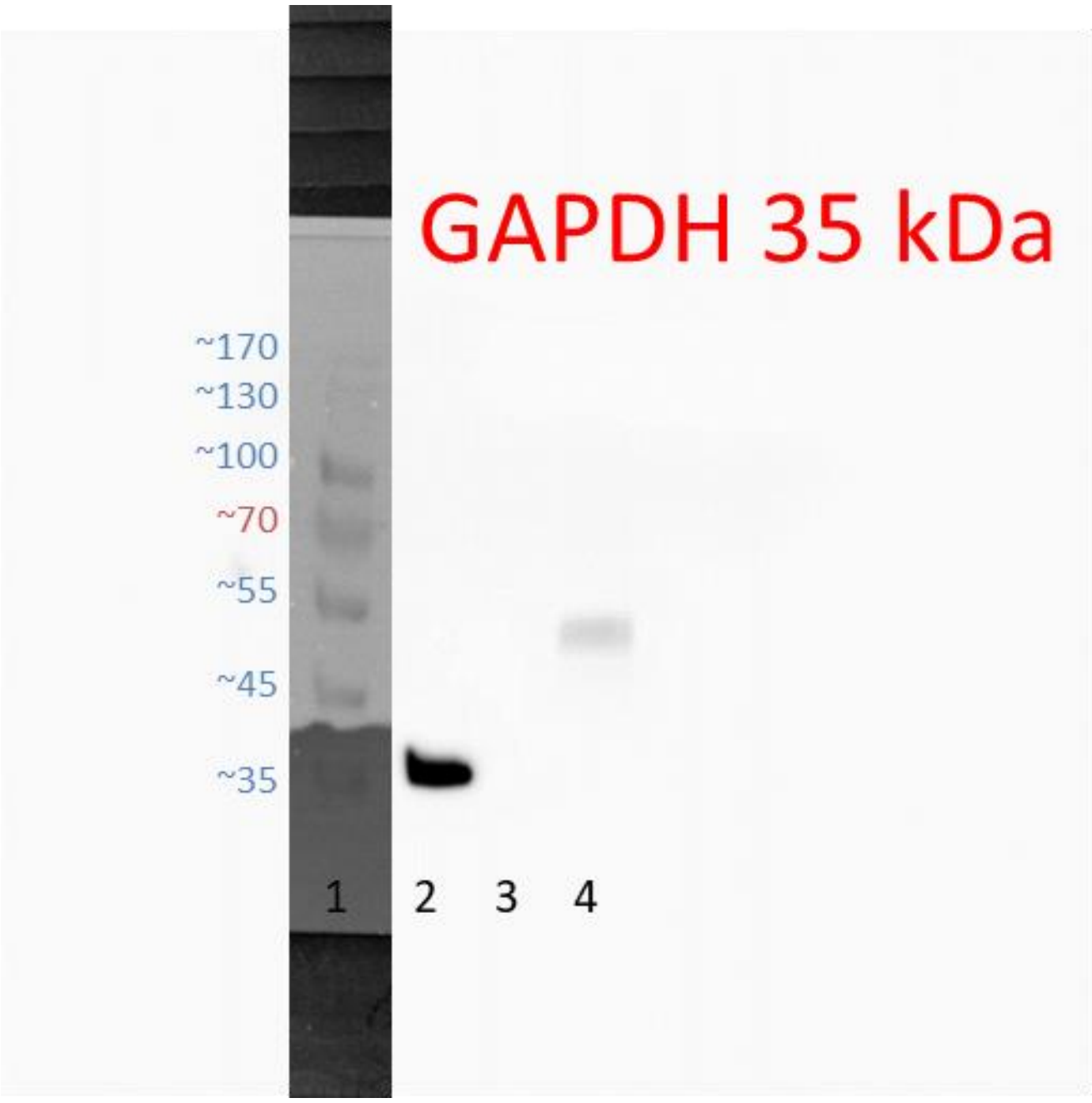

Original Western blots for Figure 6H, HT-1376 IP

Marker: PageRuler Plus Prestained #26616 Thermo Scientific

1	Marker
2	IP Lysis buffer Input
3	Rabbit lg G negative control
4	IP Lysis buffer IP

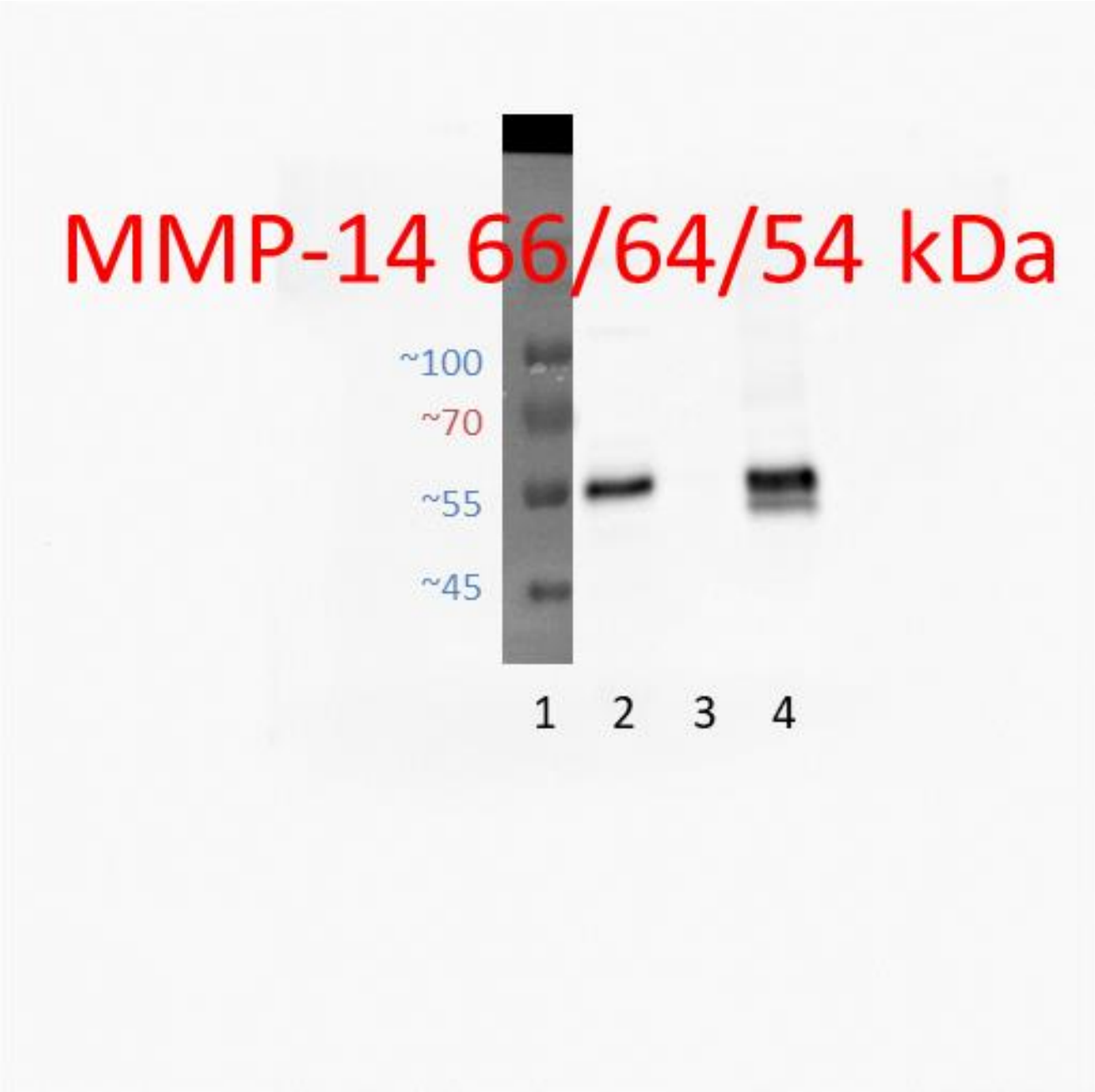

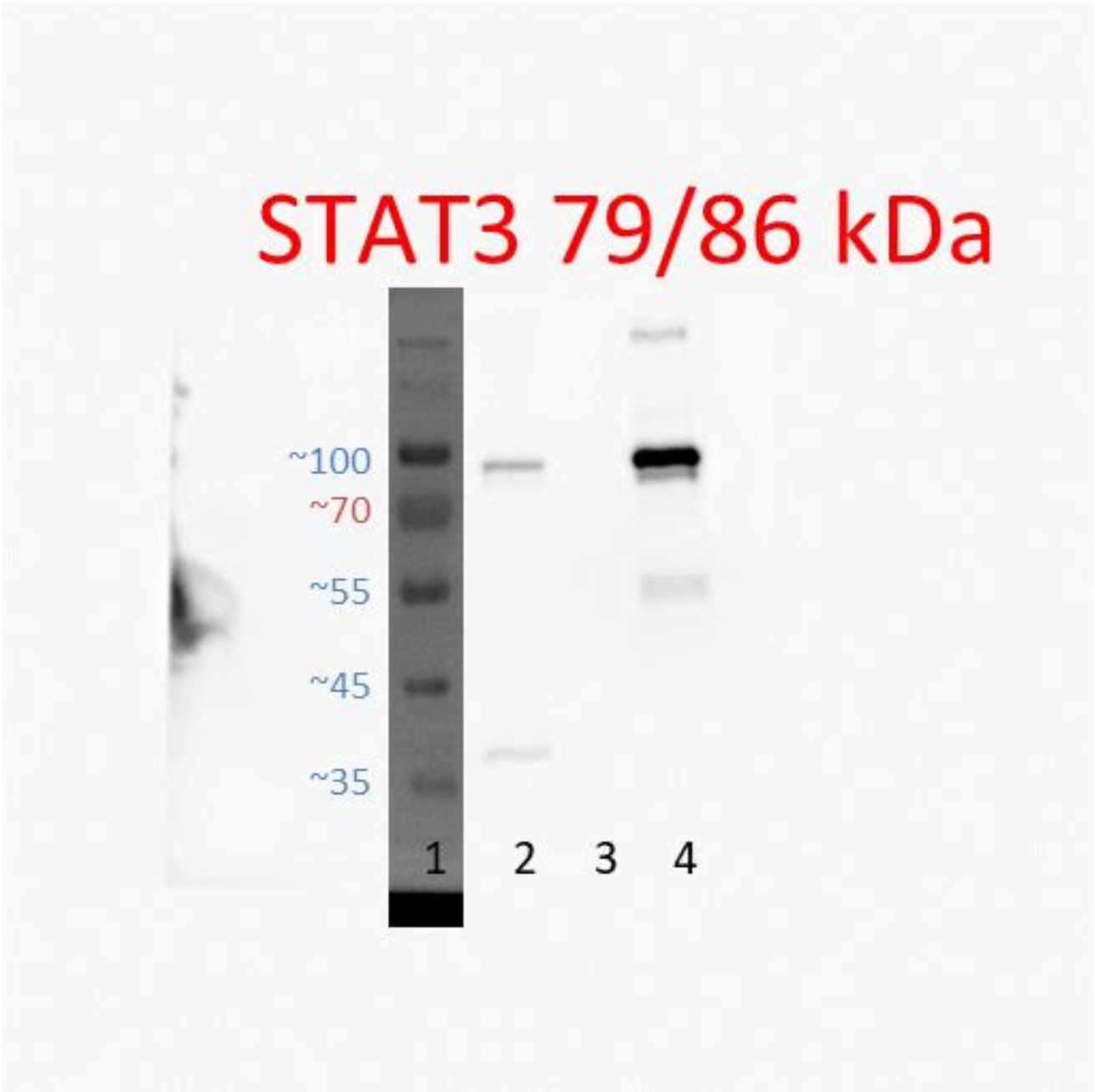

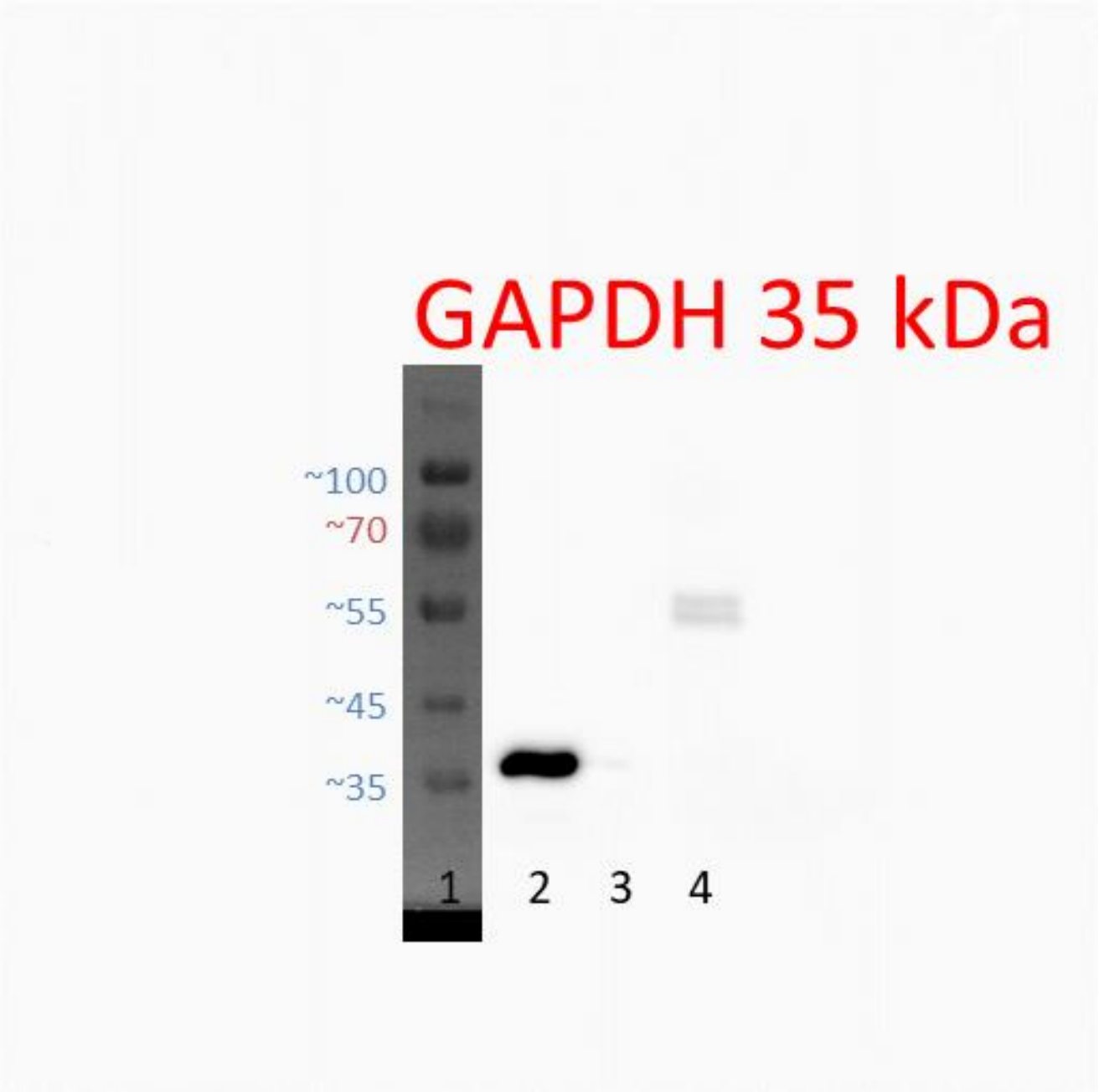

Original Western blots for Figure 6H, RT-112 IP

Marker: PageRuler Plus Prestained #26616 Thermo Scientific

1	Marker
2	IP Lysis buffer Input
3	Rabbit Ig G negative control
4	IP Lysis buffer IP

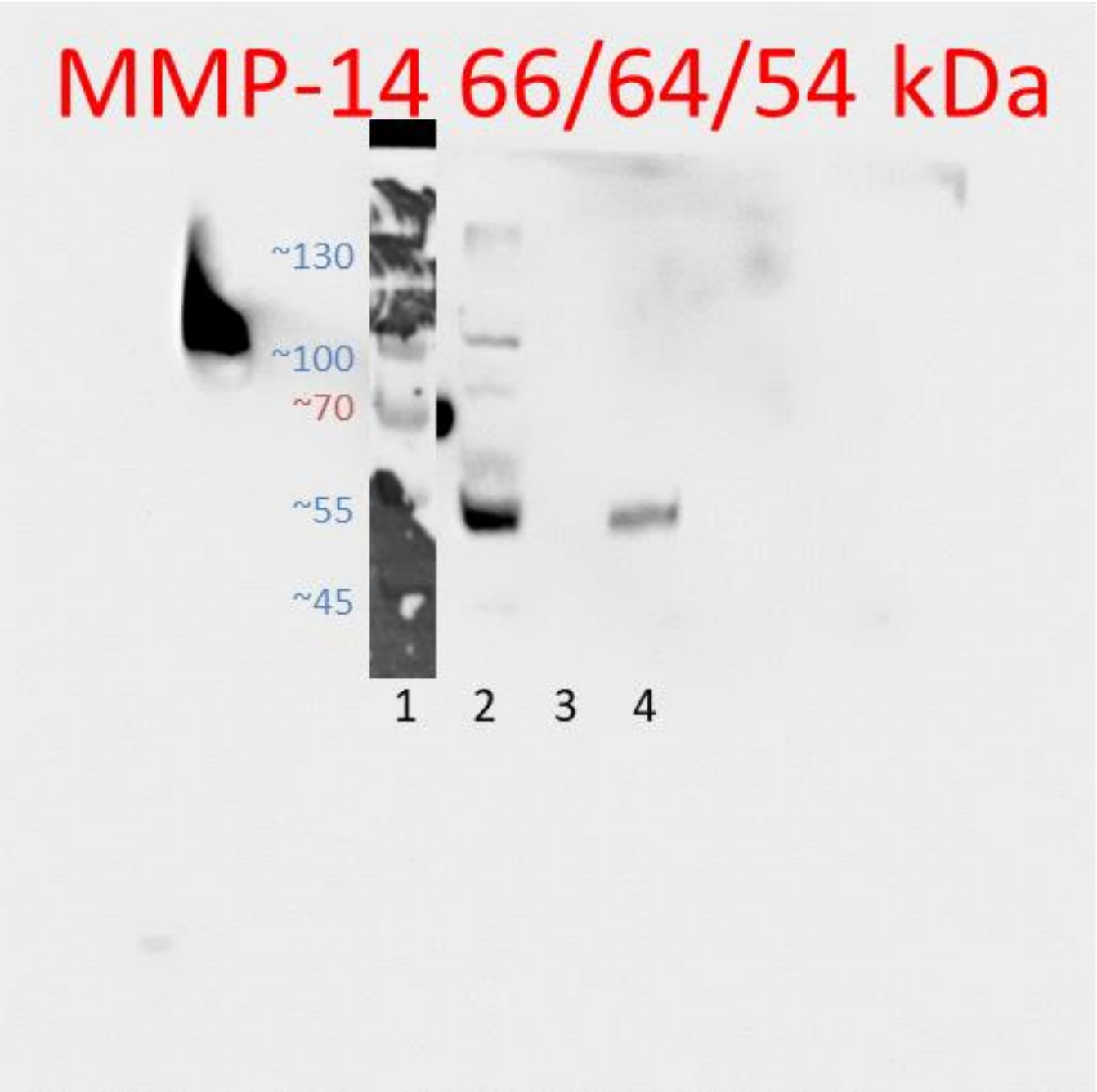

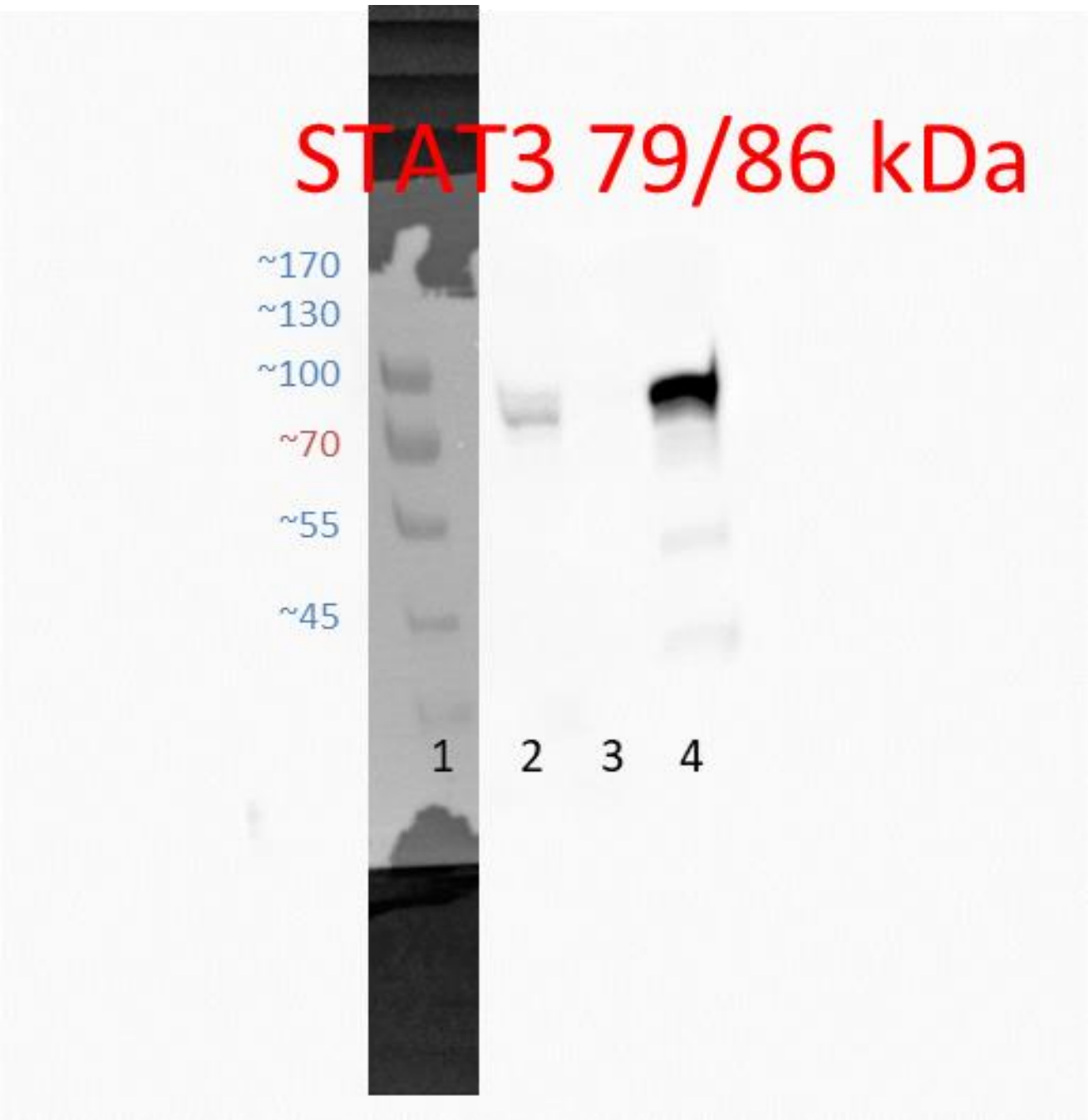

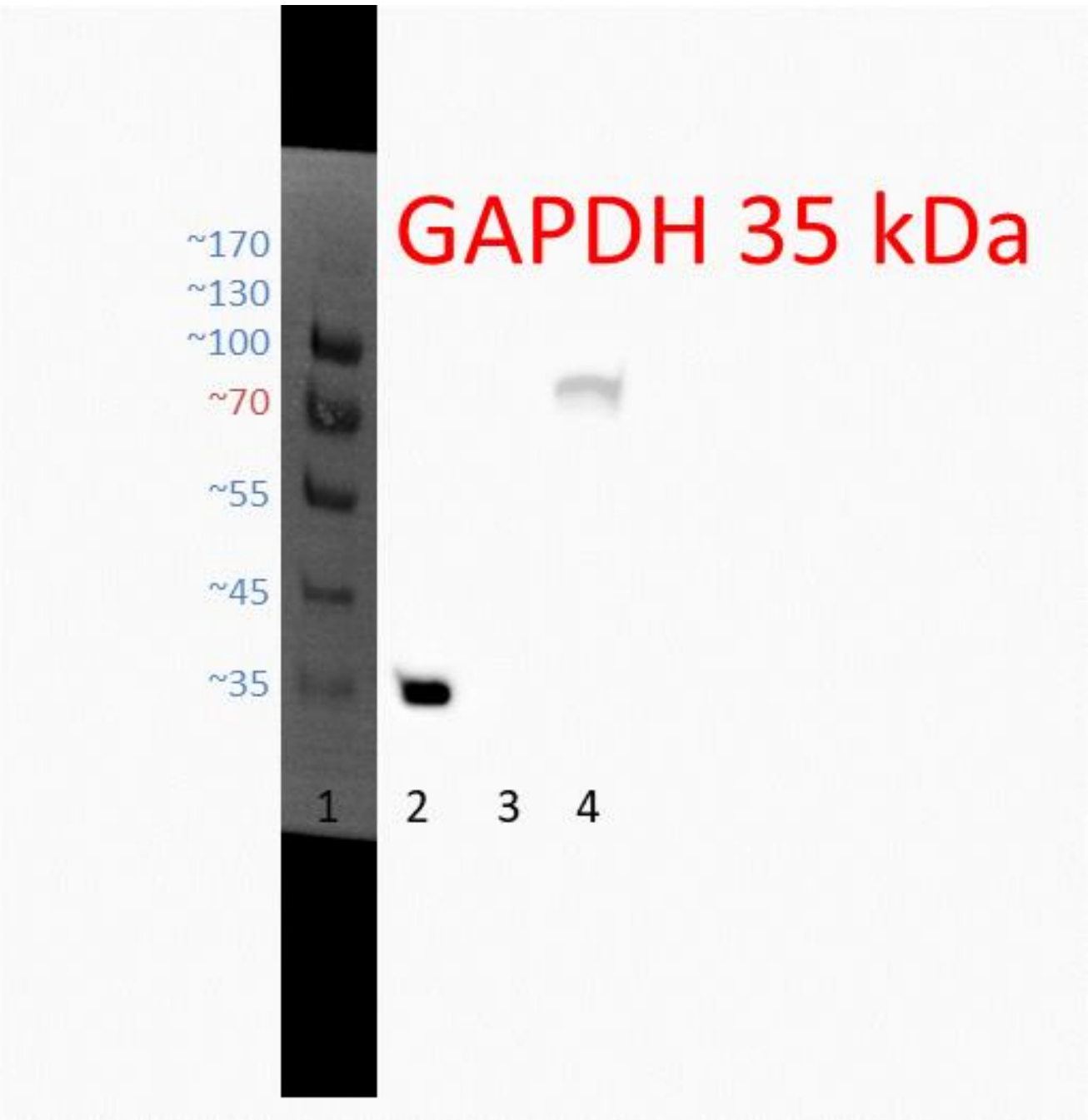

Original Western blots for Figure 7A, HT-1376 MMP-14 KD -/+ colivelin

Marker: PageRuler Prestained #26616 Thermo Scientific

1	Marker
2	HT control 1.2 5p
3	HT control 1.2 5p+colivelin
4	HT MMP14 KO 1.1 5p
5	HT MMP14 KO 1.1 5p+colivelin

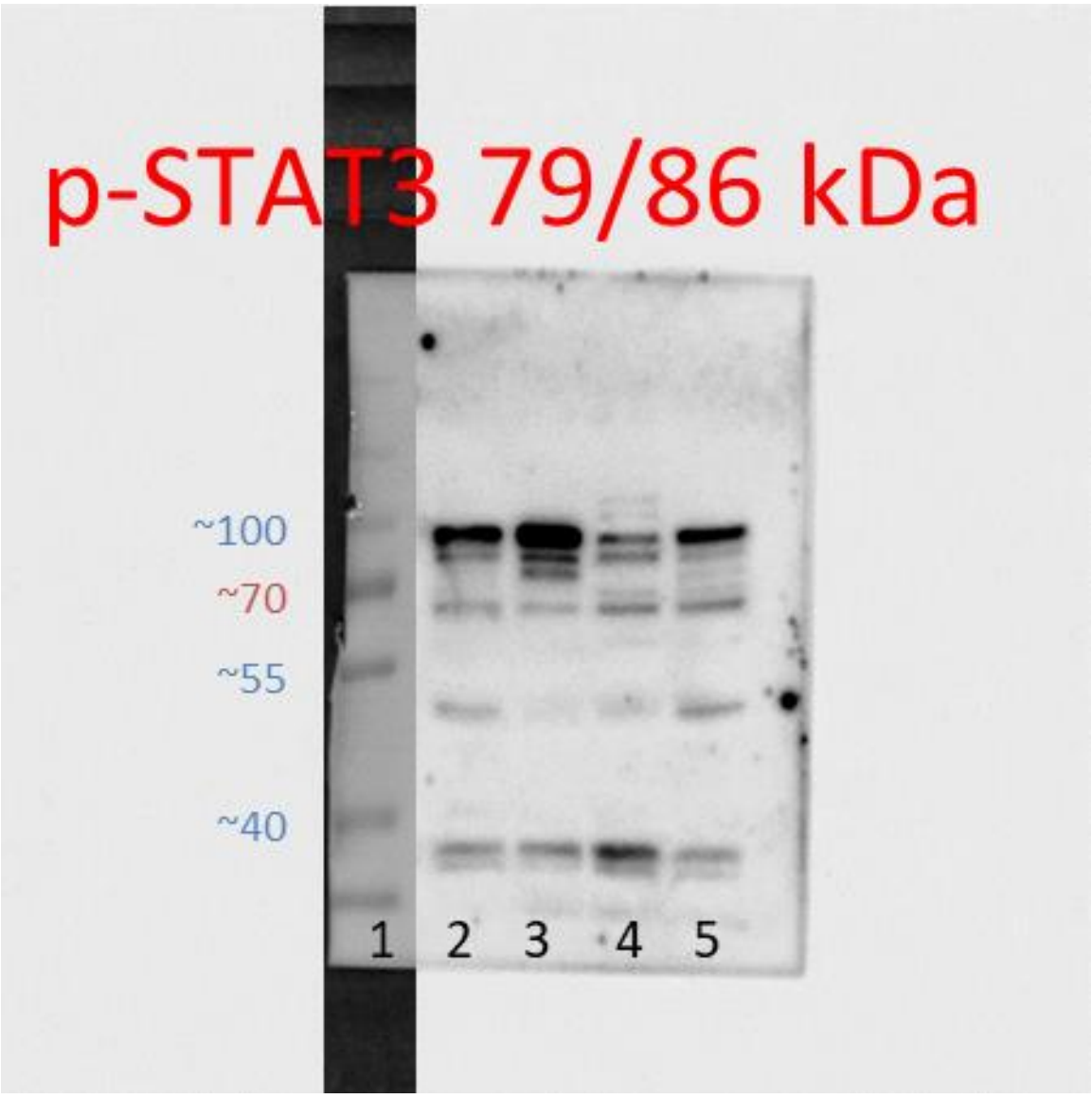

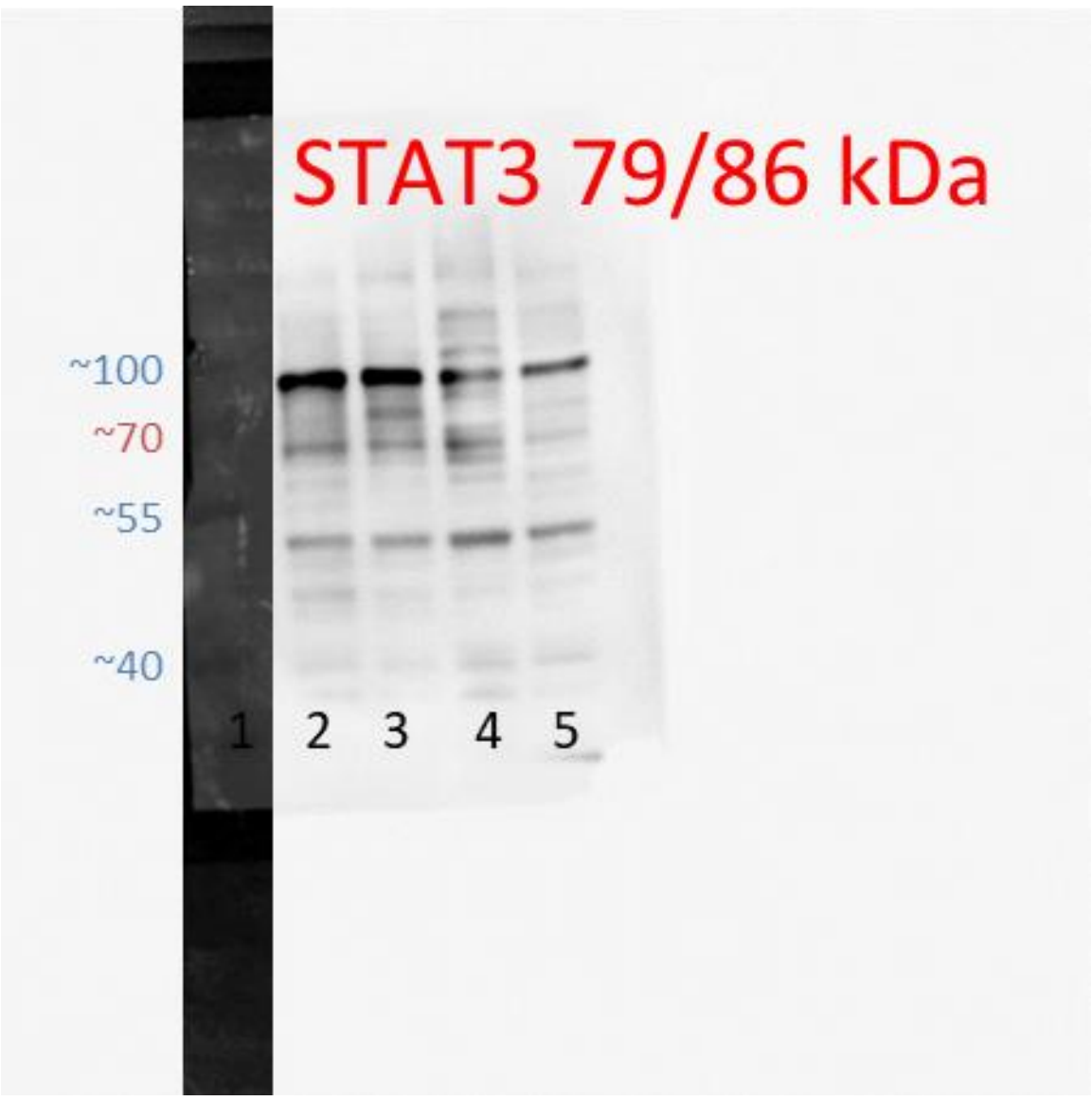

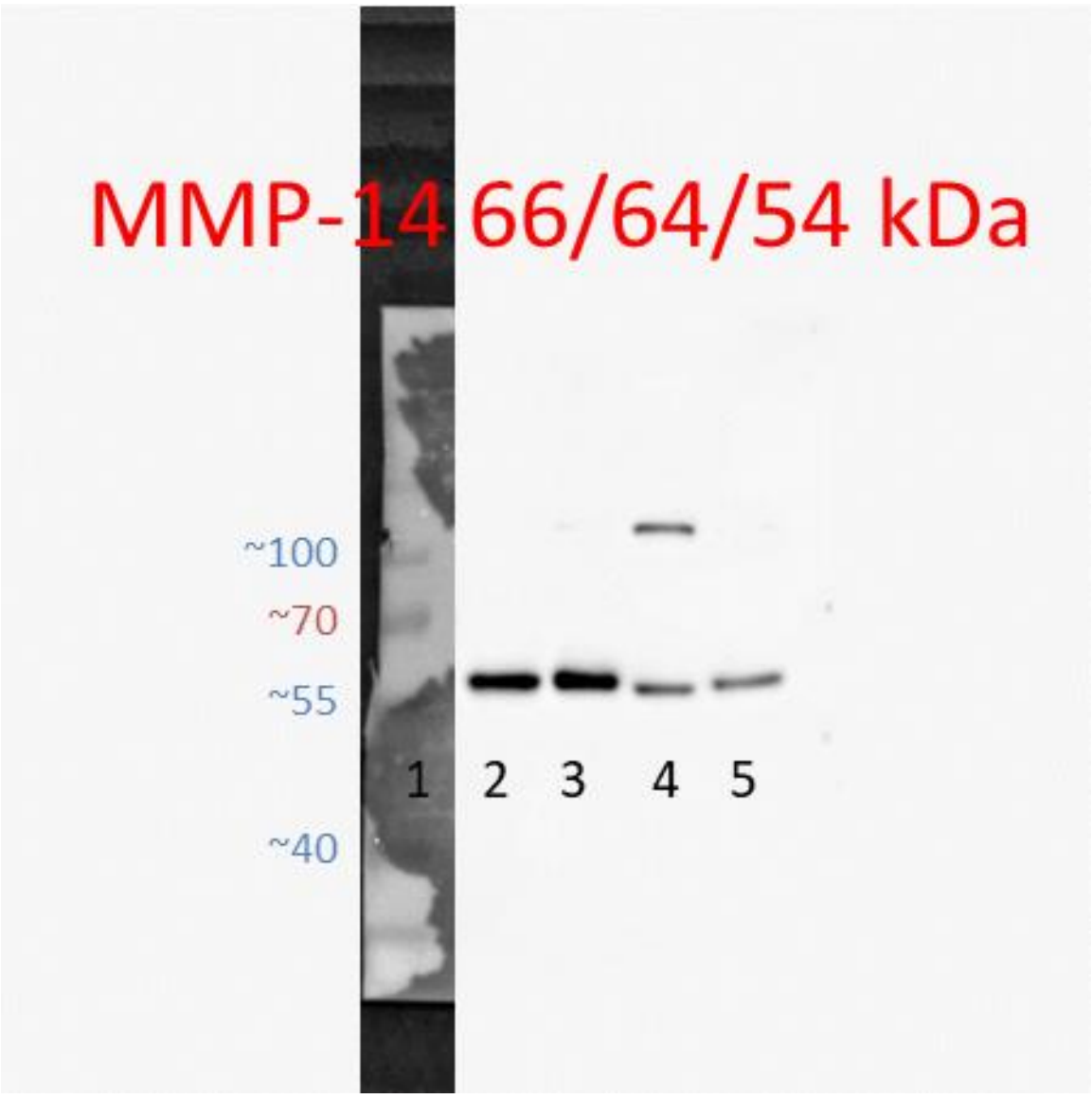

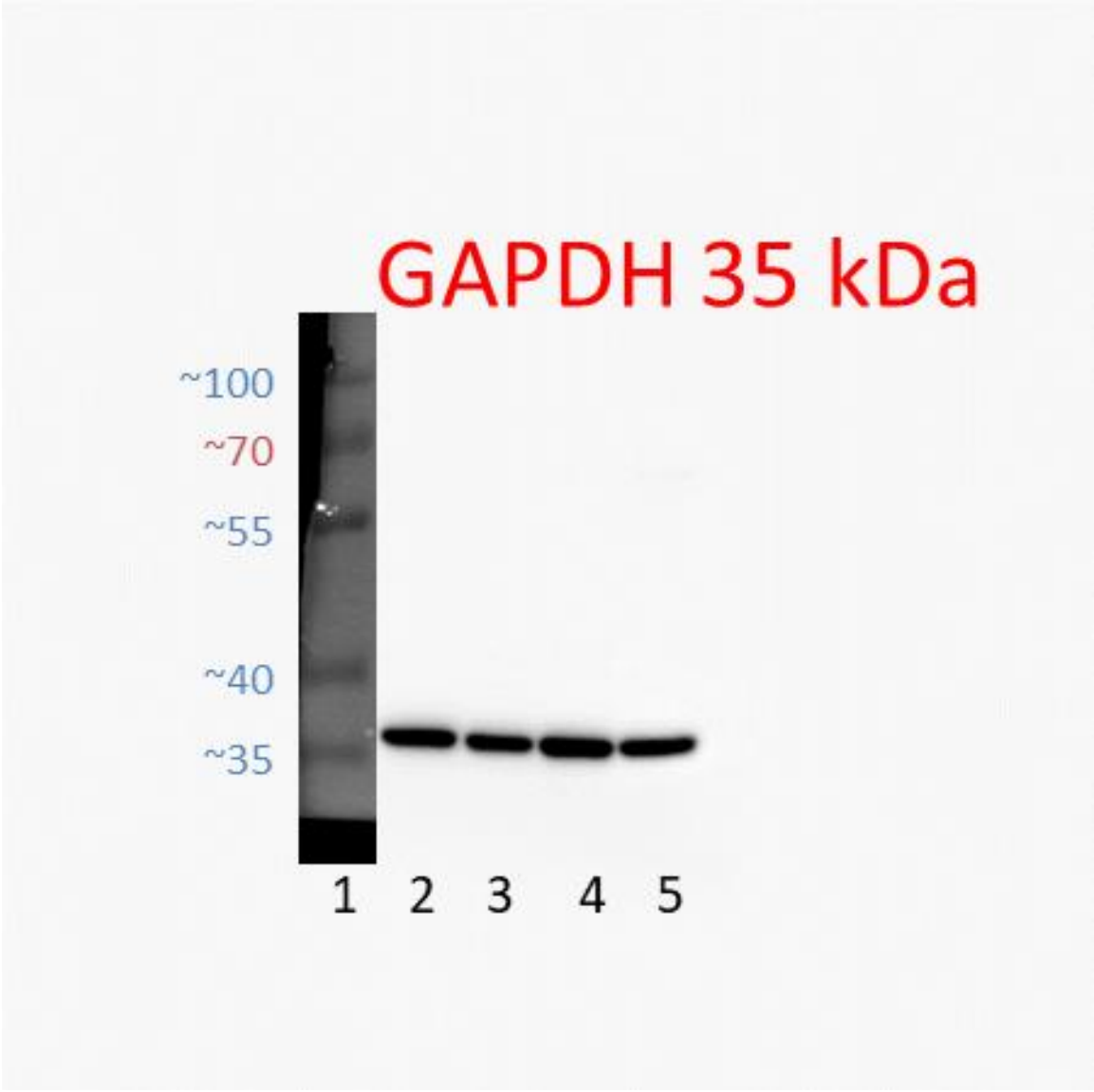

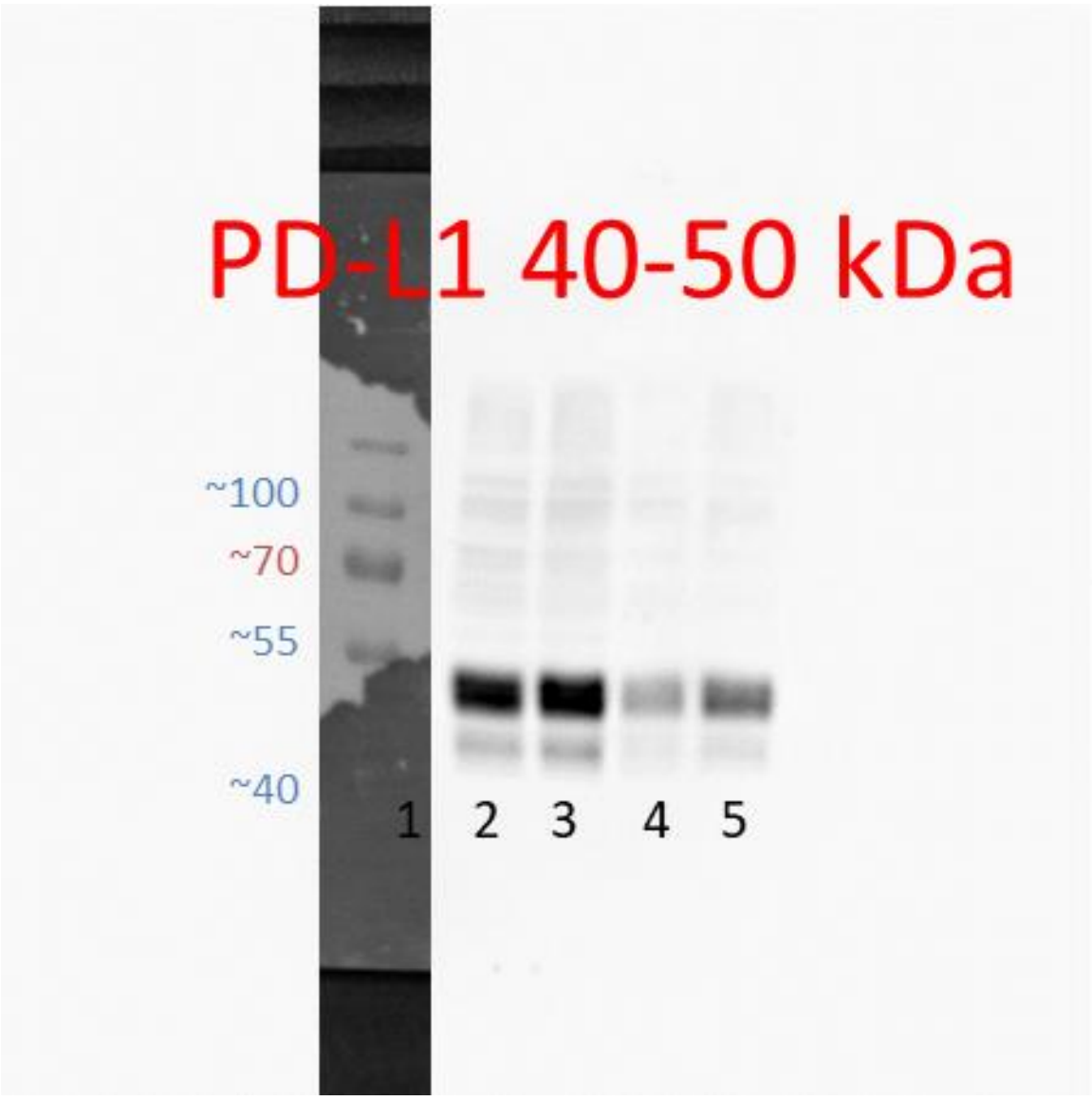

Original Western blots for Figure 7B, RT-112 MMP-14 OE -/+ HO-3867

Marker: PageRuler Prestained #26616 Thermo Scientific

1	Marker
2	RT112 vector2 4p
3	RT112 vector2 4p+HO-3867
4	RT112 OE4 4p
5	RT112 OE4 4p+HO-3867

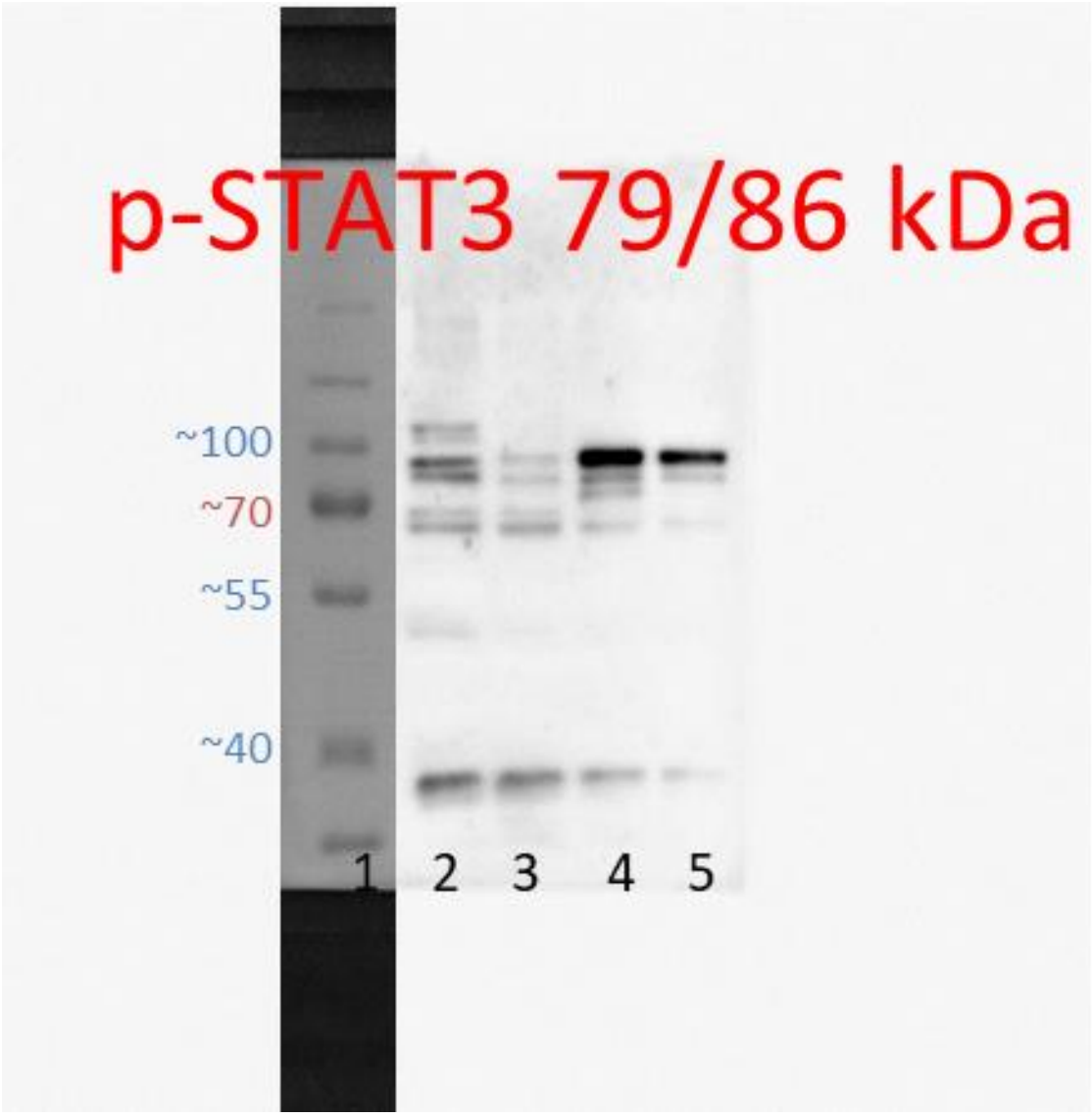

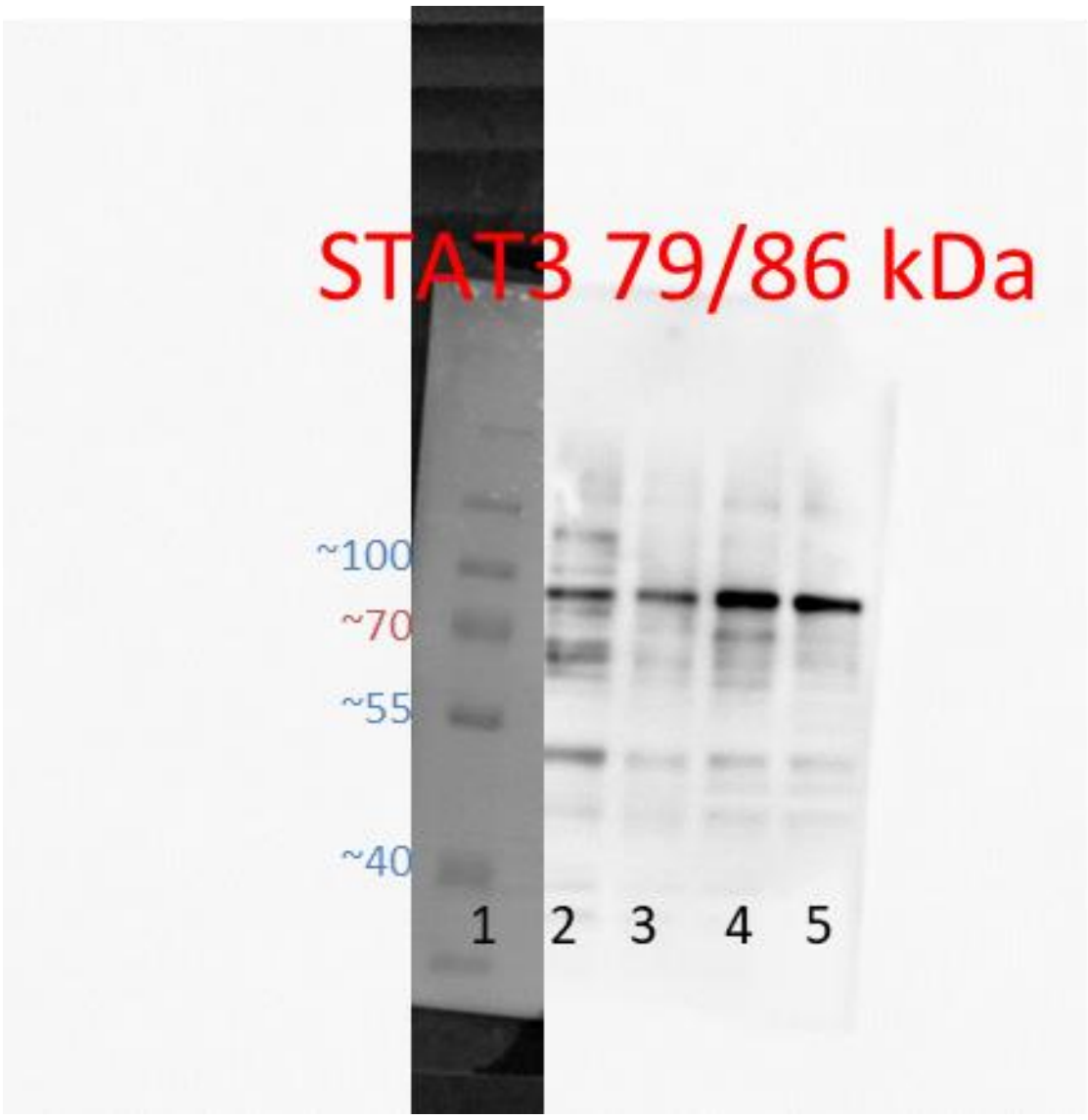

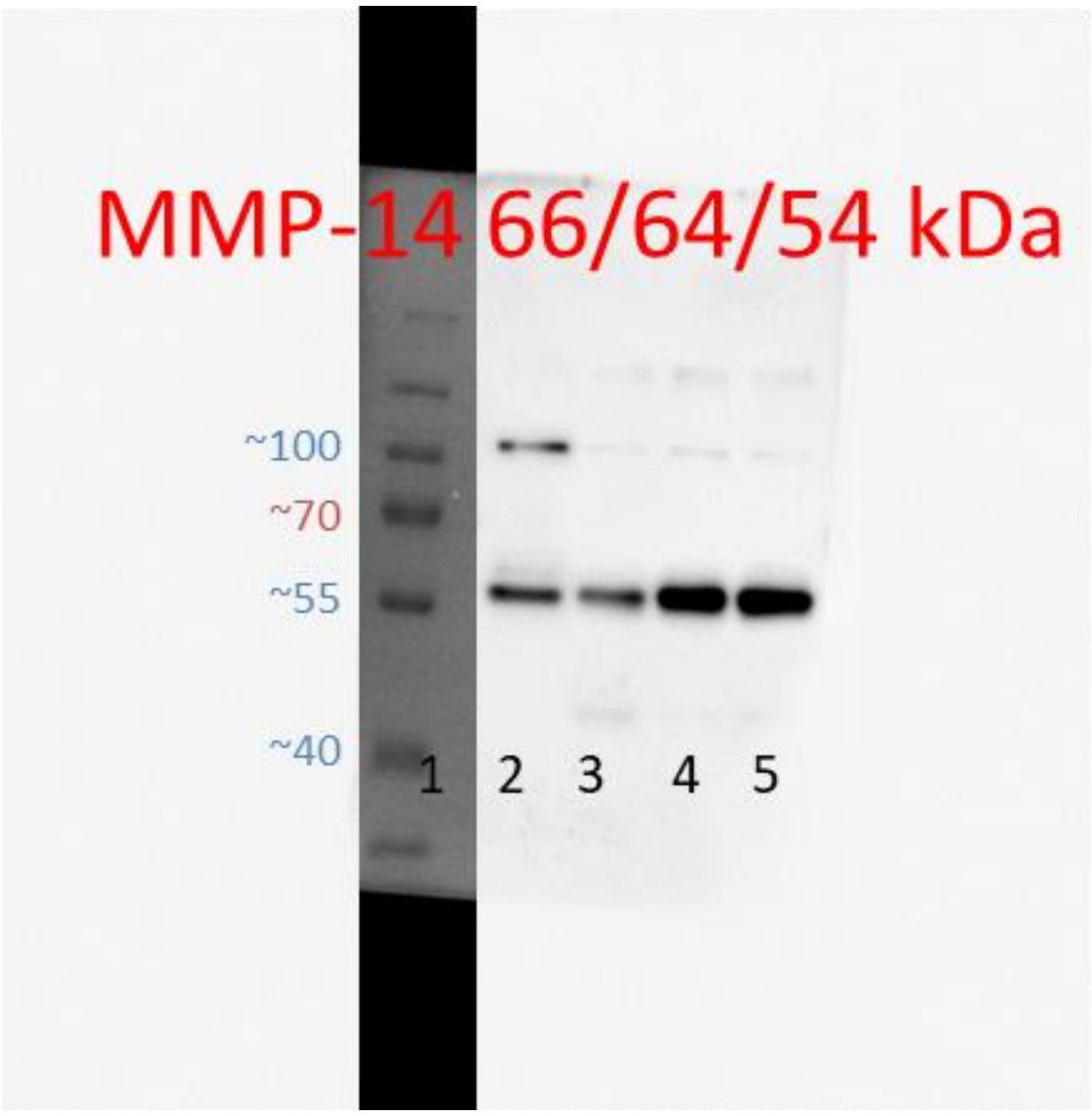

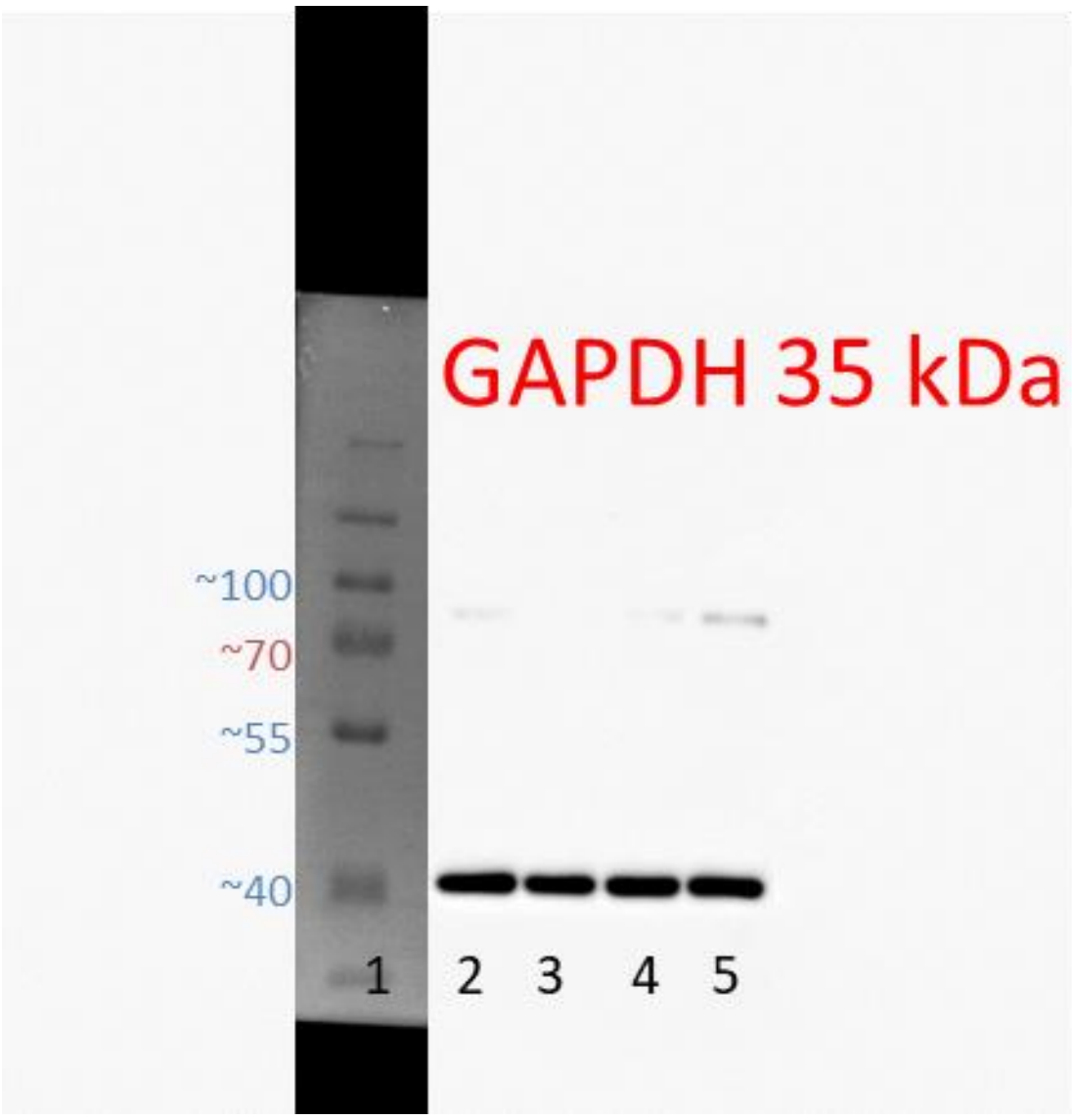

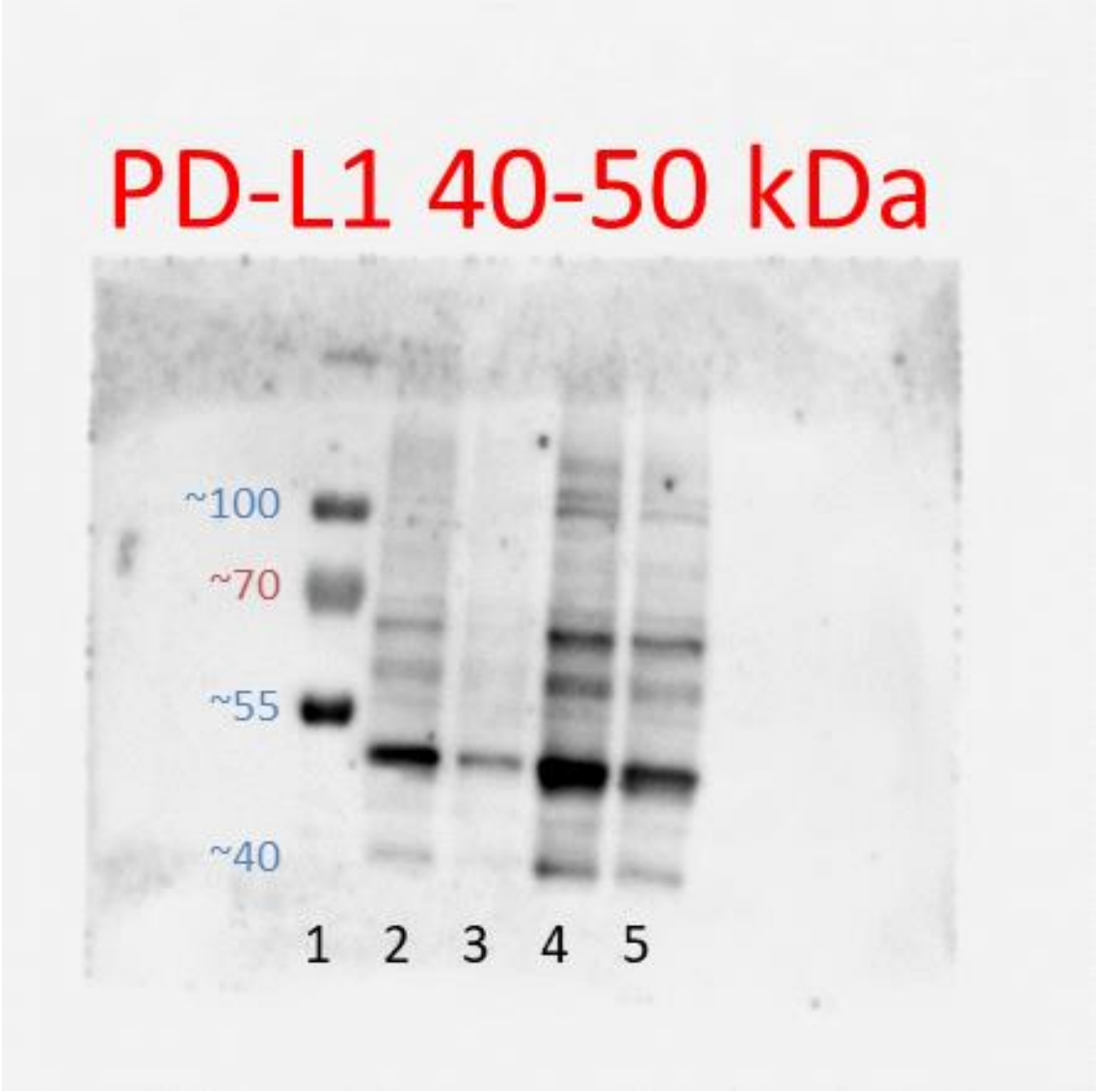

Original Western blots for Figure S5, HT-1376 IP

Marker: PageRuler (Plus) Prestained #26619 (#26616 MMP-14, p-STAT3) Thermo Scientific

1	Marker
2	IP Lysis buffer Input
3	Rabbit lg G negative control
4	IP Lysis buffer IP

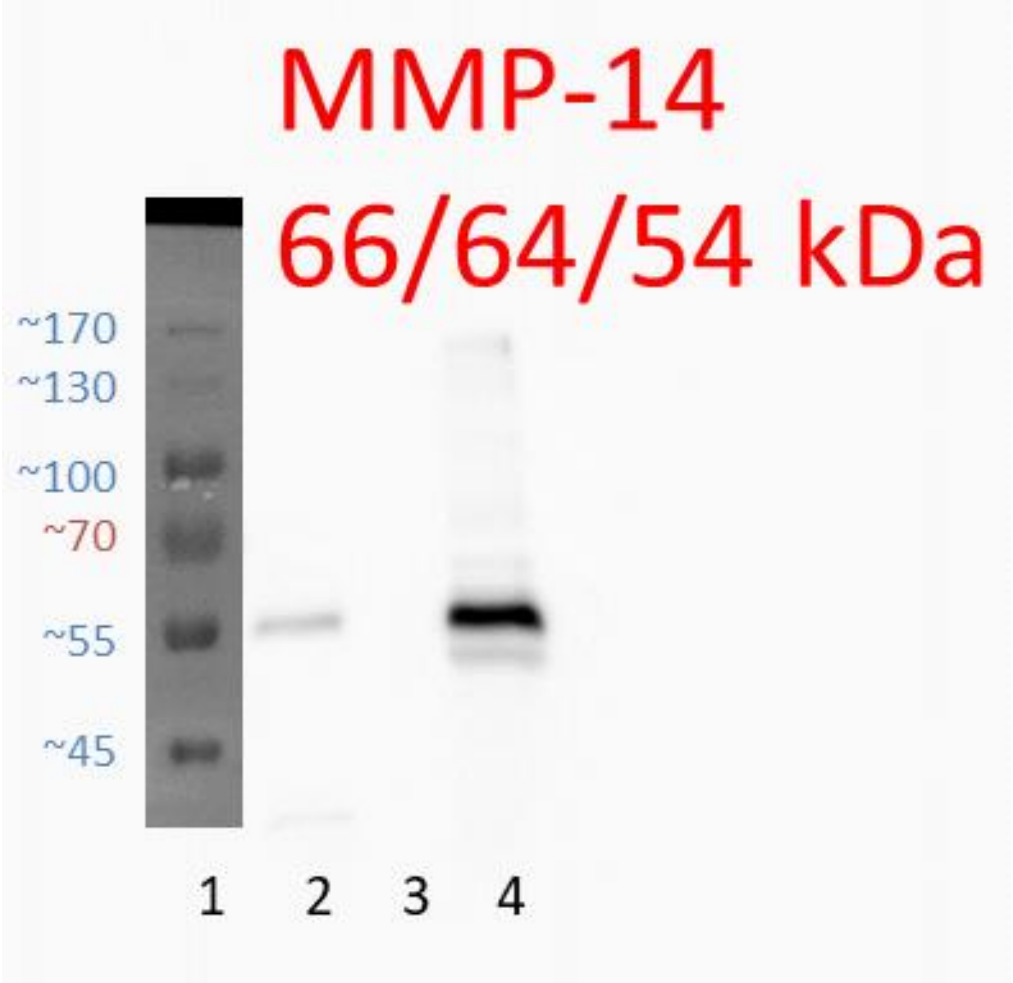

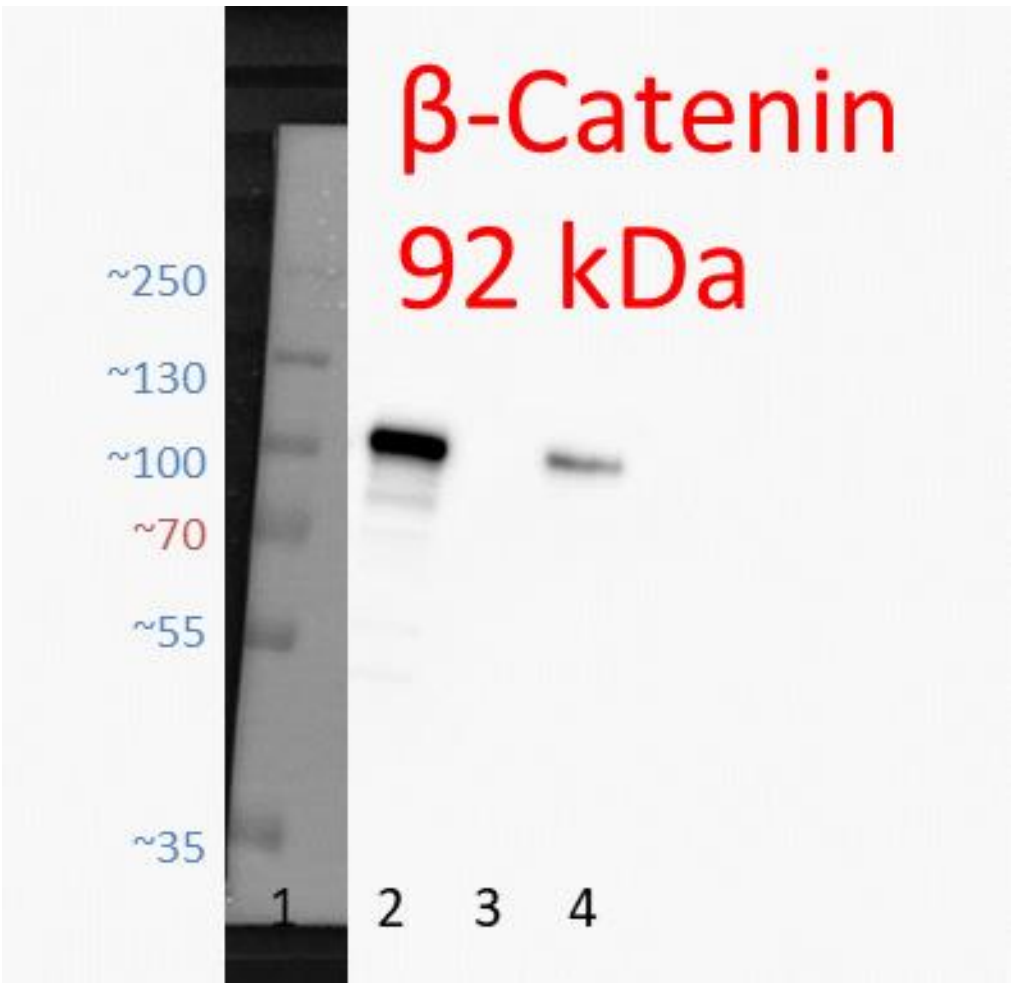

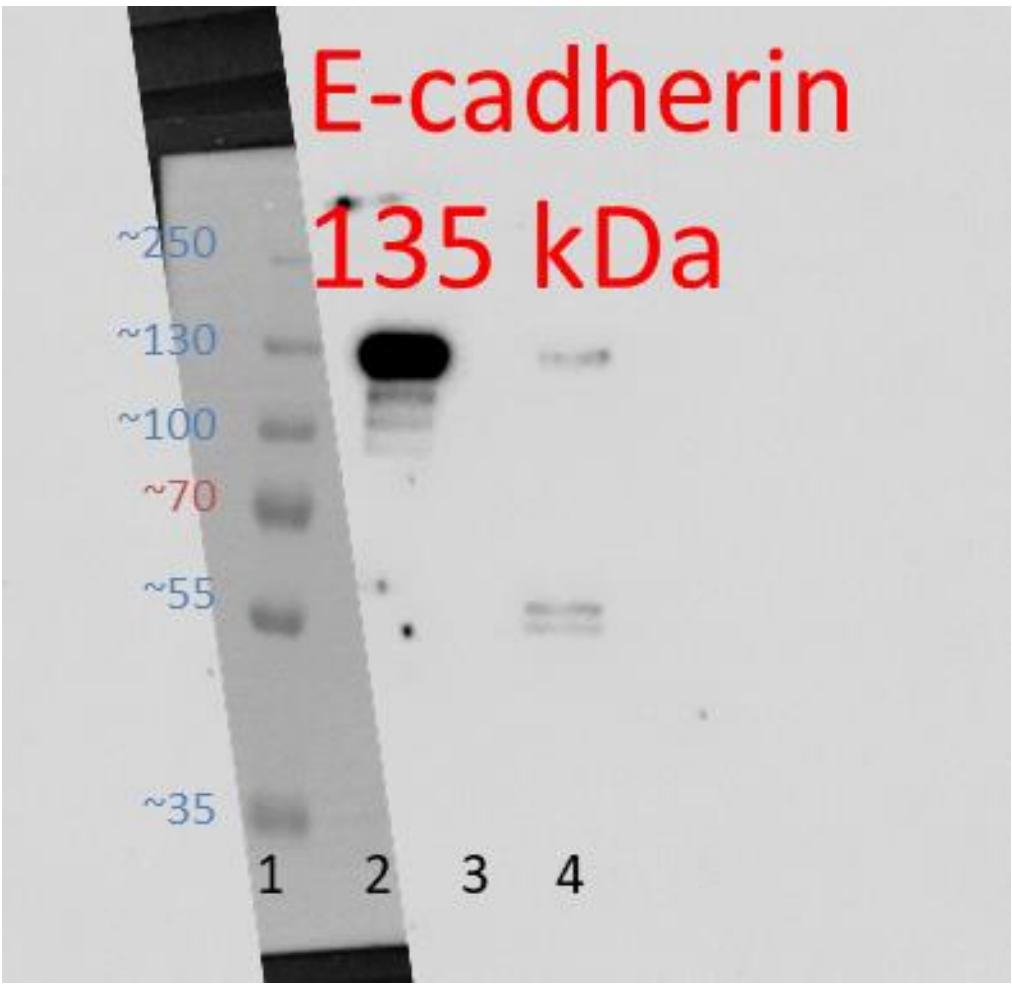

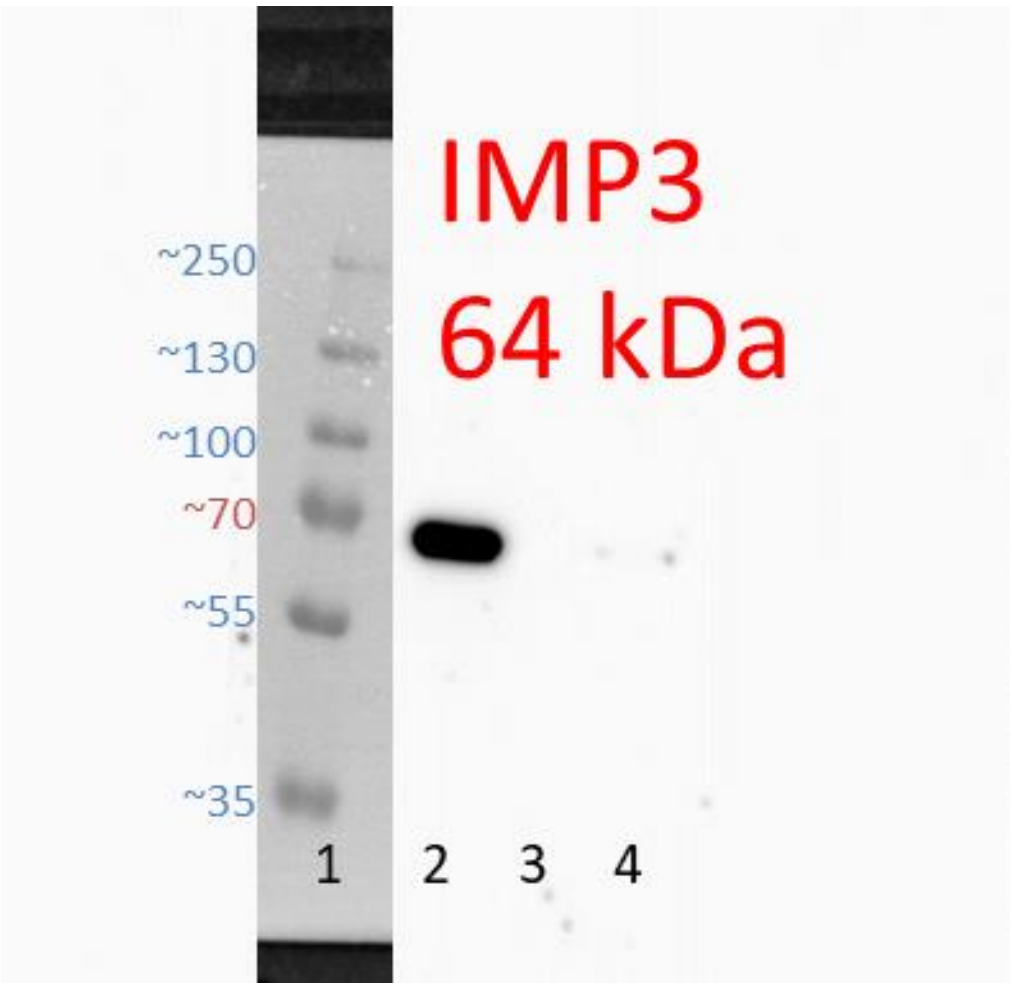

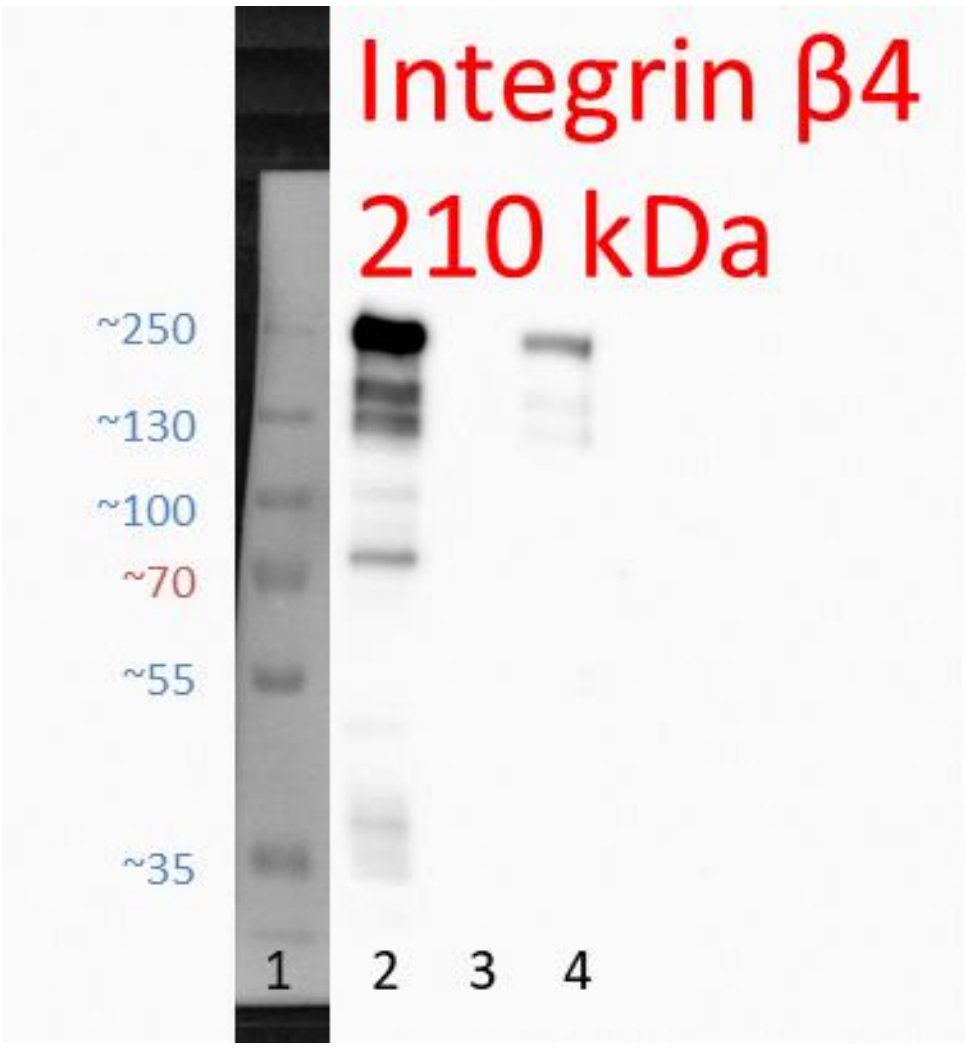

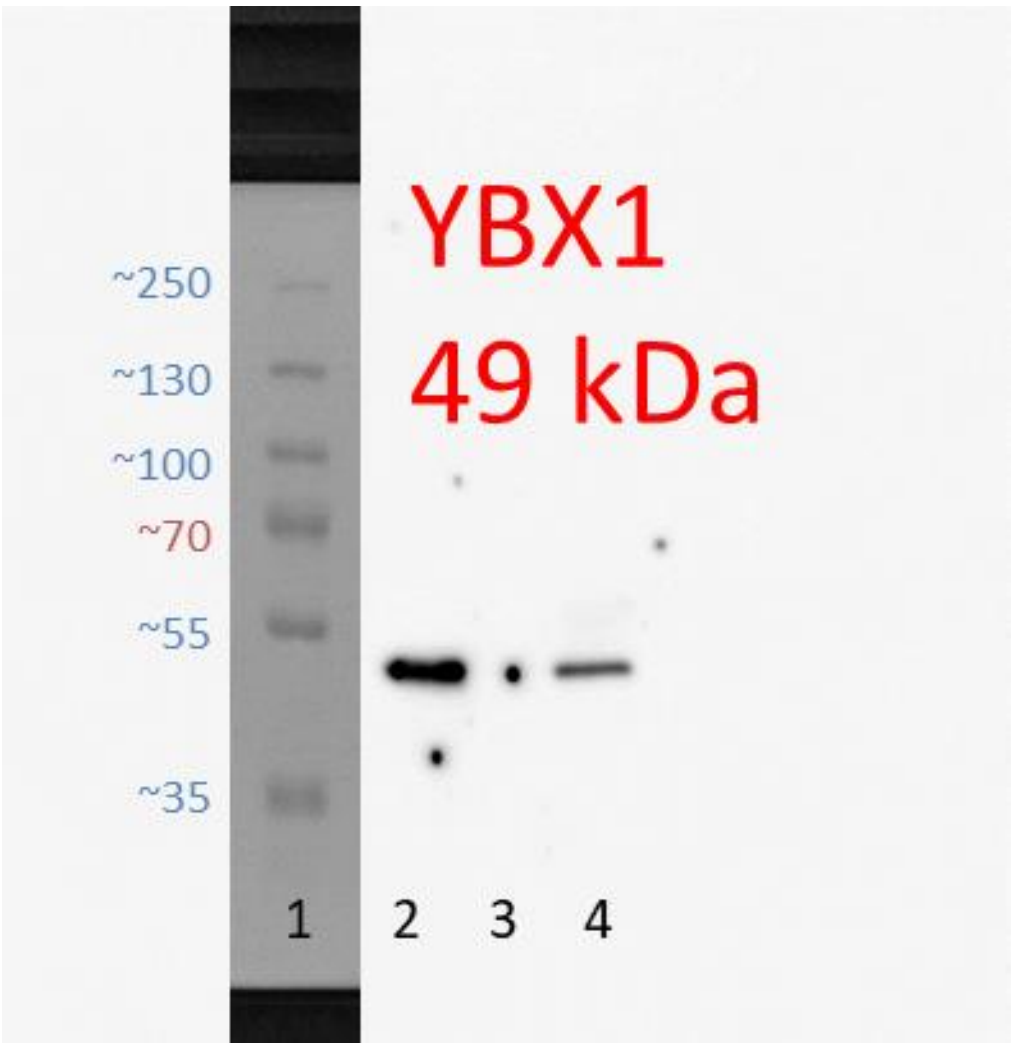

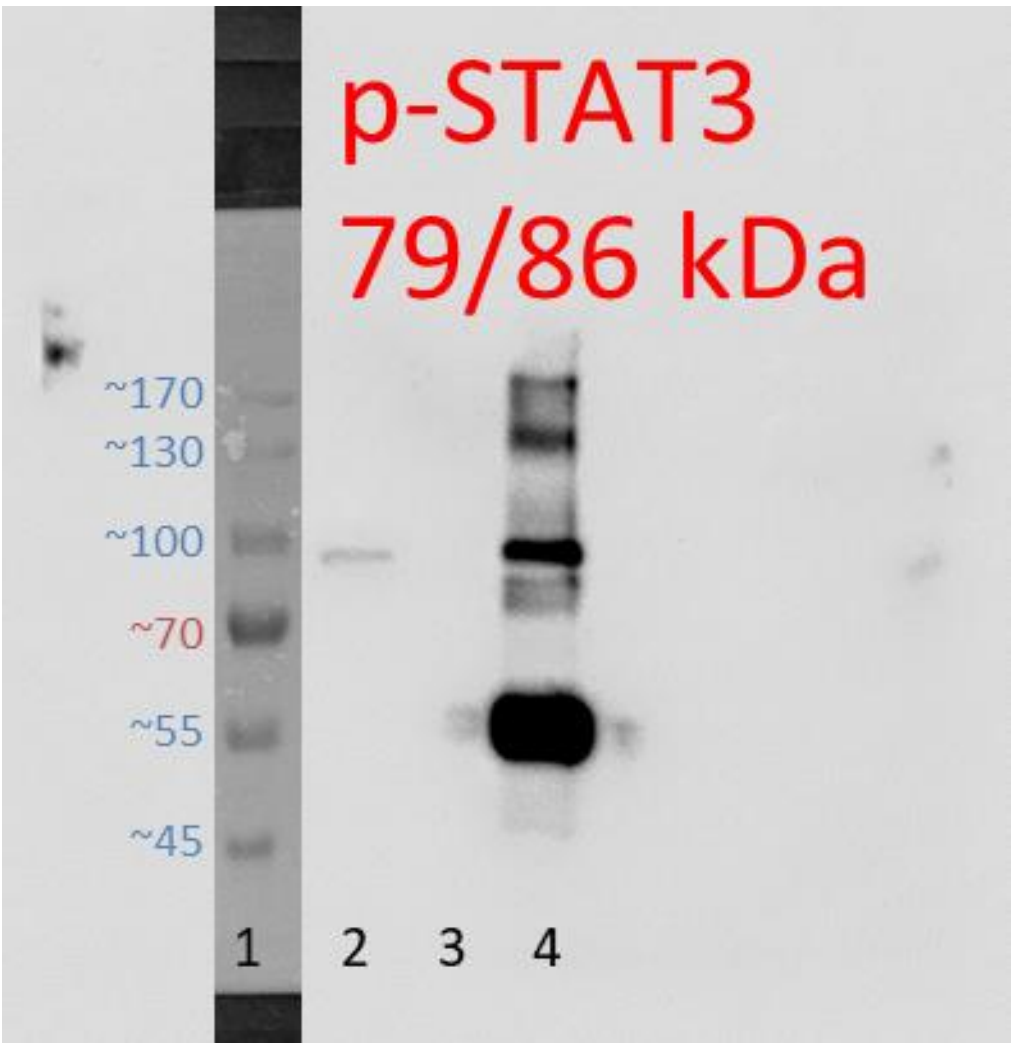

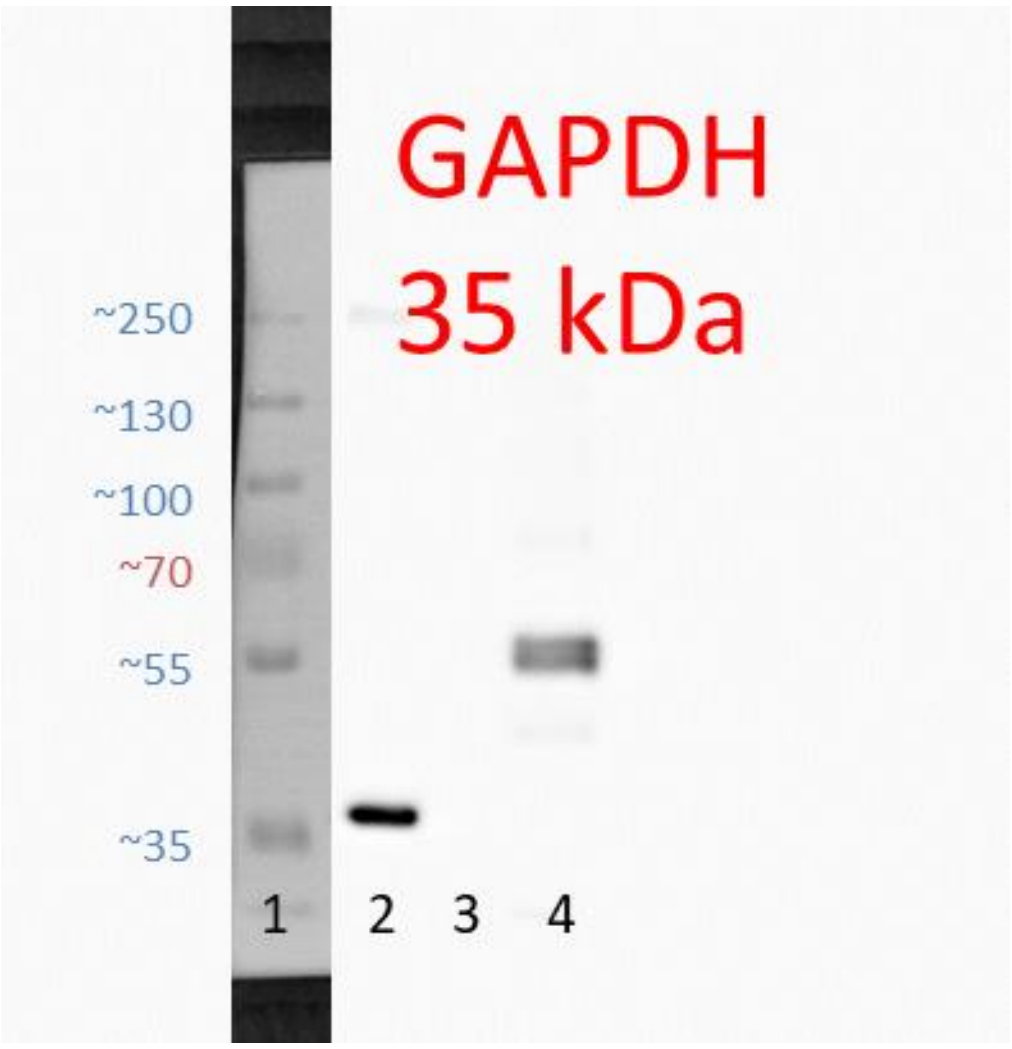

Table S1 Correlations between MMP-14 expression and clinicopathological characteristics of patients with bladder cancer (TCGA data)

Characteristic	Levels	Number (n)	Low expression of MMP-14	High expression of MMP-14	P value
			(N=207)	(N=207)	
Age, n (%)	<=70	234	122 (29.5%)	112 (27.1%)	0.372
	>70	180	85 (20.5%)	95 (22.9%)	
Gender, n (%)	Female	109	49 (11.8%)	60 (14.5%)	0.264
	Male	305	158 (38.2%)	147 (35.5%)	
Race, n (%)	Asian	44	30 (7.6%)	14 (3.5%)	0.030
	Black or African American	23	11 (2.8%)	12 (3%)	
	White	330	155 (39%)	175 (44.1%)	
Smoker, n (%)	No	109	57 (14.2%)	52 (13%)	0.676
	Yes	292	144 (35.9%)	148 (36.9%)	
T stage, n (%)	T1	5	2 (0.5%)	3 (0.8%)	0.049
	T2	119	66 (17.4%)	53 (13.9%)	
	T3	196	82 (21.6%)	114 (30%)	
	T4	60	34 (8.9%)	26 (6.8%)	
N stage, n (%)	N0	239	124 (33.5%)	115 (31.1%)	0.092
	N1	46	15 (4.1%)	31 (8.4%)	
	N2	77	38 (10.3%)	39 (10.5%)	
	N3	8	5 (1.4%)	3 (0.8%)	
M stage, n (%)	M0	202	121 (56.8%)	81 (38%)	0.362
	M1	11	5 (2.3%)	6 (2.8%)	
Pathologic stage, n (%)	Stage I	4	1 (0.2%)	3 (0.7%)	0.048
	Stage II	130	77 (18.7%)	53 (12.9%)	
	Stage III	142	68 (16.5%)	74 (18%)	
	Stage IV	136	60 (14.6%)	76 (18.4%)	
Histologic grade, n (%)	High grade	390	190 (46.2%)	200 (48.7%)	0.071
	Low grade	21	15 (3.6%)	6 (1.5%)	
Lymphovascular invasion, n (%)	No	130	66 (23.3%)	64 (22.6%)	0.892
	Yes	153	80 (28.3%)	73 (25.8%)	
Subtype, n (%)	Nonpapillary	275	124 (30.3%)	151 (36.9%)	0.005
	Papillary	134	81 (19.8%)	53 (13%)	

Characteristic	Levels	Number (n)	Low expression of MMP-14 (N=207)	High expression of MMP-14 (N=207)	P value
Radiation therapy, n (%)	No	367	177 (45.6%)	190 (49%)	0.002
	Yes	21	18 (4.6%)	3 (0.8%)	
